# Sam68 Exacerbates Pathologic Cardiac Hypertrophy by Suppressing Cardiomyocyte Glucose Oxidation

**DOI:** 10.1161/CIRCULATIONAHA.125.077533

**Published:** 2026-05-22

**Authors:** Junqing An, Chaoshan Han, Ying Jiang, Jiawei Shi, Huadong Li, Chenqi Wang, Jianrong Huang, Shiyue Xu, Jie Ni, Yangpo Cao, Yuliang Feng, Qing Lyu, Nianguo Dong, Gangjian Qin

**Affiliations:** 1 Department of Pharmacology, Homeostatic Medicine Institute, School of Medicine, Southern University of Science and Technology, Shenzhen, Guangdong, China (J.A., C.H., C.W., J.H., J.N., Y.C., Y.F., G.Q.).; 2 Departments of Cardiology (Y.J.), Union Hospital, Tongji Medical College, Huazhong University of Science and Technology, Wuhan, Hubei, China.; 3 Cardiovascular Surgery (J.S., H.L., N.D.), Union Hospital, Tongji Medical College, Huazhong University of Science and Technology, Wuhan, Hubei, China.; 4 Department of Hypertension and Vascular Disease, The First Affiliated Hospital, Sun Yat-sen University, Guangzhou, China (S.X.).; 5 Medical Research Center, Chongqing General Hospital, Chongqing University, China (Q.L.).

**Keywords:** cardiomegaly, heart failure, pyruvate dehydrogenase kinase 4, ventricular remodeling

## Abstract

**BACKGROUND:**

Metabolic remodeling, marked by maladaptive shifts in substrate use and energy production, is a hallmark of pathologic cardiac hypertrophy. Yet the mechanisms linking stress signaling to impaired myocardial glucose oxidation remain incompletely defined. Sam68 (Src-associated in mitosis, 68 kDa; also known as *KHDRBS1* [KH domain-containing, RNA-binding, signal transduction-associated protein 1]), a STAR (signal transduction and activation of RNA) family RNA-binding protein, has not previously been implicated in cardiac metabolic control.

**METHODS:**

SAM68 expression was examined in failing human hearts and transcriptomic data sets. Cardiomyocyte-specific Sam68 knockout mice (Sam68cKO) and AAV9 (adeno-associated virus serotype 9)–cTnT (cardiac troponin T)–mediated cardiomyocyte Sam68 overexpression (Sam68OE) were studied in transverse aortic constriction and angiotensin II models. Mechanistic studies included RNA sequencing, targeted metabolomics, in vivo [U-^13^C]-glucose tracing, coimmunoprecipitation, and protein–protein docking. Therapeutic relevance was tested with a PDK4 (pyruvate dehydrogenase kinase 4) inhibitor and the Sam68–Src interface blocker YB-0158, including pharmacokinetics, target engagement, and validation in Sam68cKO mice.

**RESULTS:**

Sam68 was increased in failing human cardiomyocytes and in murine hypertrophic hearts. Sam68cKO markedly attenuated angiotensin II– and transverse aortic constriction–induced hypertrophy, whereas Sam68OE aggravated remodeling and dysfunction. In vivo [U-^13^C]-glucose flux analysis showed that transverse aortic constriction caused sustained uncoupling of glycolysis from glucose oxidation, with increased glycolytic labeling but reduced ^13^C incorporation into tricarboxylic acid cycle intermediates at 3 days and 4 weeks. Sam68 deletion restored glucose-derived carbon entry into the tricarboxylic acid cycle, enhanced PDH (pyruvate dehydrogenase)–dependent M+2 labeling, and improved oxidative–anaplerotic balance during pressure overload. Mechanistically, Sam68 served as a stress-activated scaffold that promoted Src-dependent STAT3 (signal transducer and activator of transcription 3) Tyr705 phosphorylation, nuclear accumulation, and transcriptional induction of PDK4, leading to PDH Ser293 phosphorylation and suppression of PDH activity. The PDK4 inhibitor blunted Sam68OE-driven remodeling while preserving PDH activity and mitochondrial respiratory programs. YB-0158 achieved cardiac exposure, disrupted Sam68–Src engagement in vivo, suppressed STAT3–PDK4–PDH signaling, and improved transverse aortic constriction remodeling; these effects were lost in Sam68cKO mice, supporting on-target dependence. In failing human hearts, the Src–SAM68–STAT3–PDK4 axis was activated, and SAM68 abundance increased in parallel with PDK4 and reduced left ventricular ejection fraction.

**CONCLUSIONS:**

Sam68 is a stress-activated cardiomyocyte scaffold that drives pathologic hypertrophy through a Src–STAT3–PDK4 program that inhibits PDH and suppresses glucose oxidation. Genetic or pharmacologic disruption of this axis restores PDH-dependent pyruvate oxidation and limits pressure-overload remodeling, identifying Sam68 as a druggable metabolic control node in heart failure.

**Clinical Perspective**

**What Is New?**

- Sam68 (Src-associated in mitosis, 68 kDa) is a cardiomyocyte-enriched, stress-inducible scaffold that links Src to STAT3 (signal transducer and activator of transcription 3), promoting STAT3 Tyr705 phosphorylation, nuclear signaling, and PDK4 (pyruvate dehydrogenase kinase 4) induction during pressure overload.
- Natural abundance–corrected in vivo [U-13C]-glucose tracing reveals that *Sam68* deletion restores PDH (pyruvate dehydrogenase)–dependent glucose carbon entry into the tricarboxylic acid cycle and improves the oxidative/anaplerotic balance during transverse aortic constriction.
- The Src–Sam68–STAT3–PDK4 axis is therapeutically targetable: selective PDK4 inhibition and pharmacologic disruption of the Sam68–Src interface (YB-0158) attenuate pressure overload–induced hypertrophy and dysfunction.

**What Are the Clinical Implications?**

- The Src–Sam68–STAT3–PDK4 pathway provides a mechanistic link between stress signaling and PDH inhibition with impaired glucose oxidation, defining an actionable metabolic checkpoint in pathologic hypertrophy and heart failure.
- Therapeutic targeting of Sam68 or downstream blockade of STAT3/PDK4 may restore PDH activity and glucose oxidation, thereby limiting maladaptive remodeling and potentially slowing progression from hypertrophy to heart failure.

The heart sustains extraordinarily high adenosine triphosphate (ATP) demand to support continuous contraction.^1,2^ Healthy cardiomyocytes meet this demand through metabolic flexibility, switching among fatty acids, glucose, lactate, ketone bodies, and amino acids.^3^ This flexibility is disrupted in pathologic hypertrophy: fatty acid oxidation (FAO) declines, glycolysis increases, and the 2 pathways become uncoupled, contributing to energy deficit and progression to heart failure (HF).^4,5^ Under physiologic conditions, ≈60% to 90% of cardiac ATP is derived from long-chain FAO, whereas glucose oxidation contributes ≈10% to 30%.^2^ During hypertrophic stress, the myocardium becomes increasingly glycolytic,^6^ yet the additional pyruvate generated is not efficiently oxidized in mitochondria.^7^ This failure of glucose oxidation is driven largely by persistent phosphorylation and inactivation of PDH (pyruvate dehydrogenase).^8^ When glycolysis is uncoupled from oxidation, glucose-derived carbon is diverted into bypass pathways (eg, the hexosamine biosynthetic pathway), whose metabolites fuel prohypertrophic signaling and structural remodeling.^9^ Thus, restoring glucose oxidation is a compelling therapeutic strategy.

PDH is the enzymatic gate that links glycolysis to the tricarboxylic acid (TCA) cycle by catalyzing the irreversible conversion of pyruvate to acetyl-CoA.^1^ In adult cardiomyocytes, PDH activity is regulated primarily by reversible phosphorylation of the E1α subunit (S293, S232, and S300): PDH is phosphorylated and inhibited by PDKs (pyruvate dehydrogenase kinases; PDK1 [pyruvate dehydrogenase kinase 1], PDK2 [pyruvate dehydrogenase kinase 2], and PDK4 [pyruvate dehydrogenase kinase 4]) and dephosphorylated and activated by PDPs (pyruvate dehydrogenase phosphatases; PDP1 [pyruvate dehydrogenase phosphatase 1] and PDP2 [pyruvate dehydrogenase phosphatase 2]).^10^ Among PDK isoforms, PDK4 is a principal mediator of PDH inhibition in response to hypertrophic stimuli, such as phenylephrine and angiotensin II (Ang II).^11,12^ Sustained PDK4 upregulation is a hallmark of pressure-overload hypertrophy and HF with preserved ejection fraction, tracking with mitochondrial dysfunction and poor prognosis.^13,14^ Cardiac PDK4 overexpression erodes metabolic flexibility and worsens disease,^14,15^ whereas genetic or pharmacologic PDK4 inhibition reactivates PDH, restores coupling of glycolysis to oxidation, and improves left ventricular (LV) performance.^13,16^ Despite this rationale, the upstream signaling mechanisms driving pathologic PDK4 induction during pressure overload remain incompletely defined.

Sam68 (Src-associated in mitosis, 68 kDa; also known as KHDRBS1 [KH domain-containing, RNA-binding, signal transduction-associated protein 1]) is a member of the STAR (signal transduction and activation of RNA) family. Through its KH and QUA domains, Sam68 binds RNA and regulates transcription, alternative splicing, and nuclear export.^17,18^ Sam68 also contains proline-rich motifs that bind SH3 domains of Src family kinases, enabling tyrosine phosphorylation and recruitment of SH2-containing partners; thus, Sam68 can function as a scaffold coupling tyrosine kinase signaling to downstream gene-regulatory programs.^18^ Although best studied in cancer and neuronal contexts,^19,20^ accumulating evidence—including our own—supports metabolic roles for Sam68, including regulation of aerobic glycolysis in tumors,^21^ hepatic gluconeogenesis,^22^ adipocyte differentiation,^23^ and adaptive thermogenesis.^24^ Whether Sam68 controls mitochondrial pyruvate utilization and contributes to cardiac hypertrophy has remained unknown.

Here we demonstrate that Sam68 is a stress-activated metabolic rheostat in the pressure- overloaded heart. Sam68 scaffolds Src and STAT3 (signal transducer and activator of transcription 3), enabling Src-dependent STAT3 Tyr705 phosphorylation, nuclear accumulation, and transcriptional induction of PDK4. PDK4 upregulation then sustains PDH inhibition, suppresses pyruvate oxidation, and accelerates hypertrophic remodeling. Genetic cardiomyocyte Sam68 deletion, selective PDK4 inhibition, or pharmacologic blockade of the Src–Sam68 interface restores PDH activity, improves oxidative metabolism, and attenuates pressure overload–induced hypertrophy and dysfunction. These findings establish Sam68 as a previously unrecognized regulator of cardiac energetics and a druggable target for metabolic rescue in HF.

## Methods

Supplemental Methods are provided in the Supplemental Material.

### Human Heart Samples

LV tissue was obtained from patients with HF due to restrictive cardiomyopathy or hypertrophic cardiomyopathy undergoing transplantation and from nonfailing organ donors whose hearts were declined for transplantation (Table S9). The study conformed to the Declaration of Helsinki and was approved by the ethics committee of Union Hospital, Tongji Medical College, Huazhong University of Science and Technology (approval UHCT-IEC-SOP-016-03-01). Written informed consent was obtained from transplant patients and donors’ next of kin.

### Data Availability

Raw RNA sequencing (RNA-seq) FASTQ files have been deposited in the National Center for Biotechnology Information Sequence Read Archive under BioProject PRJNA1393250. Targeted metabolomics data have been deposited in MetaboLights under accession numbers MTBLS13836 and MTBLS13840. Raw densitometry tables and metabolomics peak-area tables are provided as Supplemental Material. Additional information is available from the corresponding author upon reasonable request.

### Statistical Analysis

Data are presented as mean±SEM. Normality was assessed using the Shapiro-Wilk test and homogeneity of variance was evaluated using the Brown-Forsythe test (or *F* test, where appropriate). For 2-group comparisons, unpaired 2-tailed Student *t* tests were used for normally distributed data with equal variances, and Welch *t* tests were used when variances were unequal. For >2 groups with 1 factor, 1-way ANOVA was followed by the Tukey multiple comparison test (all pairwise comparisons) or Dunnett test (comparisons to a single control), as specified. For experiments with 2 or 3 independent factors, 2-way or 3-way ANOVA was used, followed by the Tukey multiple comparison test for all pairwise comparisons or the Sidak test for prespecified comparisons, as indicated. When parametric assumptions were not met and could not be adequately addressed by data transformation, factorial analyses were performed using Aligned Rank Transform ANOVA. Aligned Rank Transform ANOVA was implemented using R (version 4.5.0) with the ARTool package (version 0.11.2). Holm-adjusted Mann-Whitney *U* tests were used for post hoc pairwise comparisons after Aligned Rank Transform ANOVA. For analyses assessing associations while accounting for group status, simple linear regression models including group (HF versus nonfailing) as a covariate were used; this approach is equivalent to partial correlation controlling for group status. Statistical analyses were performed using GraphPad Prism (version 10.0) and R (version 4.5.0). A 2-sided *P*<0.05 was considered statistically significant.

## Results

### Sam68 Is Selectively Upregulated in Cardiomyocytes During Hypertrophy and HF

**Figure 1.**
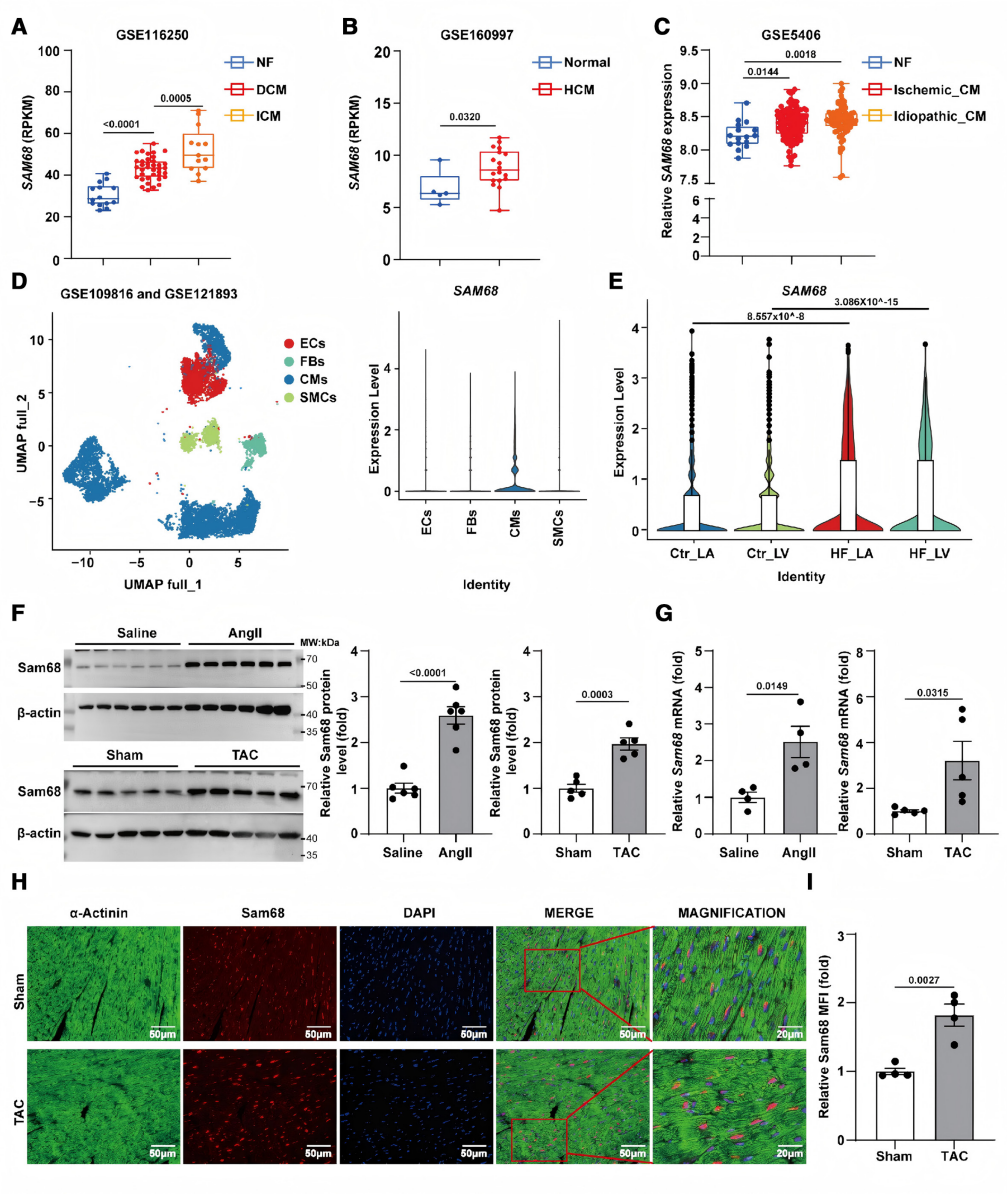
**Sam68 is upregulated in myopathic and failing human and mouse hearts. A** through **C**, Bulk RNA sequencing or microarray quantification of *SAM68* (Src-associated in mitosis, 68 kDa; also known as *KHDRBS1* [KH domain-containing, RNA-binding, signal transduction-associated protein 1]) in left ventricular tissue. **A**, GSE116250: nonfailing (NF; n=14), dilated cardiomyopathy (DCM; n=37), and ischemic cardiomyopathy (ICM; n=13). **B**, GSE160997: normal (n=5) vs hypertrophic cardiomyopathy (HCM; n=18). **C**, GSE5406: nonfailing (n=16), DCM (n=108), and ICM (n=86). **D** and **E**, Single-cell RNA sequencing (GSE109816 and GSE121893). **D**, Major cardiac cell types (left) and *SAM68* mRNA abundance by cell type (right). **E**, *SAM68* mRNA levels in cardiomyocytes from left atrium (LA) and left ventricle (LV) of controls (Ctr) and heart failure (HF) samples. **F** through **I**, Mouse hearts after 2-week angiotensin II (Ang II) infusion or 4-week transverse aortic constriction (TAC). **F**, Representative immunoblots and quantification of Sam68. **G**, Reverse transcription polymerase chain reaction of *Sam68* mRNA (normalized to 18S). **H**, Immunofluorescence (Sam68, α-Actinin, and DAPI). **I**, Quantification of Sam68 mean fluorescence intensity (MFI) in **H** (n=4). Molecular weight markers (kDa) are indicated on immunoblots. Data are mean±SEM. One-way ANOVA with the Tukey test (**A** and **C**), Student *t* test (**B**, **F**, **G**, and **I**), Aligned Rank Transform ANOVA with Holm-adjusted post hoc tests (**E**). CM indicates cardiomyocyte; EC, endothelial cell; FB, fibroblast; and SMC, smooth muscle cell.

To determine whether *SAM68* expression tracks with human cardiac disease, we analyzed 3 independent LV bulk transcriptomic data sets (GSE116250, GSE160997, and GSE5406) encompassing nonfailing hearts and samples from dilated cardiomyopathy, ischemic cardiomyopathy, and hypertrophic cardiomyopathy.^25–27^ *KHDRBS1* (*SAM*68) transcripts were markedly elevated in all disease cohorts compared with nonfailing controls (Figure 1A–1C).

We next sought to determine which cardiac cell type accounted for this increase. Single-cell RNA-seq data sets from the left ventricle and atrium of 12 healthy donors and 8 patients with HF (GSE109816 and GSE121893) resolved major cardiac populations, including cardiomyocytes, endothelial cells, fibroblasts, and smooth muscle cells (Figure S1A and S1B). *SAM68* expression was enriched in cardiomyocytes relative to nonmyocytes (Figure 1D) and increased predominantly in cardiomyocytes in HF, with minimal changes in endothelial cells, fibroblasts, or smooth muscle cells (Figure 1E and Figure S1C).

We validated these observations in mice. Ang II infusion or transverse aortic constriction (TAC) increased *Sam68* mRNA and protein abundance in whole-heart lysates (Figure 1F and 1G). Sam68 protein was elevated at 1 week after TAC and continued to increase through 4 weeks (Figure S1D). In neonatal rat ventricular myocytes (NRVMs), Ang II or phenylephrine similarly increased Sam68 protein (Figure S1E and S1F). Immunofluorescence confirmed predominant nuclear localization of Sam68 in cardiomyocytes and showed marked enhancement of nuclear Sam68 signal after TAC (Figure 1H and 1I). These data collectively establish that Sam68 is upregulated in cardiomyocytes during hypertrophy and HF, with a pronounced accumulation in the nucleus.

### Cardiomyocyte-Specific Sam68 Deletion Protects Adult Mice From TAC-Induced Dysfunction

**Figure 2.**
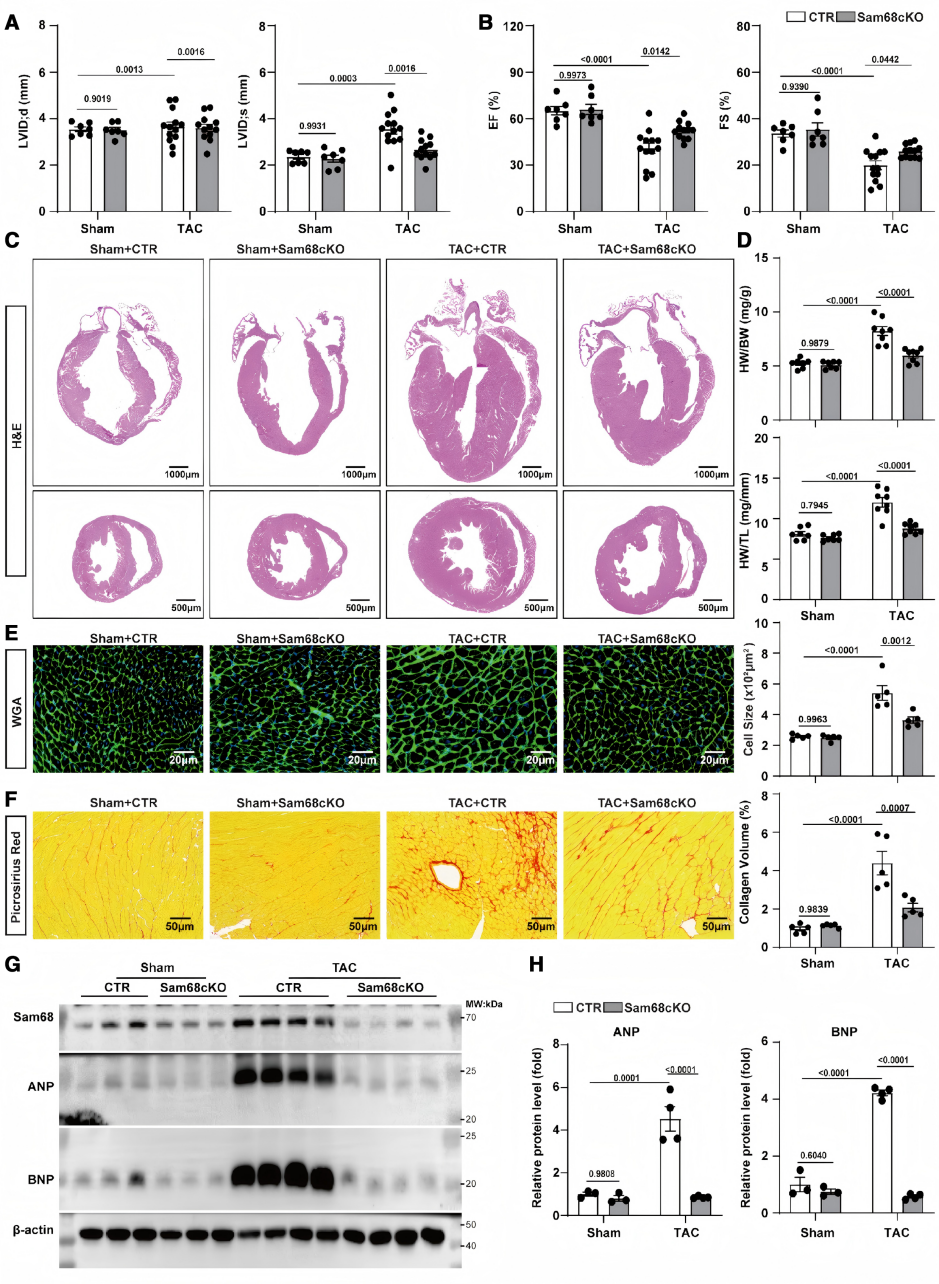
**Cardiomyocyte *Sam68* deletion prevents transverse aortic constriction–induced hypertrophy and dysfunction.** Control mice (CTR) and cardiomyocyte-specific knockout mice (Sam68^flox/flox^;Myh6-MerCreMer [Sam68cKO]) underwent a sham procedure or transverse aortic constriction (TAC) and were analyzed 4 weeks later. **A** and **B**, Echocardiography results. **A**, Left ventricular internal diameter at end-diastole (LVID;d) and end-systole (LVID;s). **B**, Ejection fraction (EF) and fractional shortening (FS) (sham+CTR, n=7; sham+Sam68cKO, n=7; TAC+CTR, n=13; TAC+Sam68cKO, n=12). **C**, Representative hematoxylin & eosin (H&E) staining (longitudinal and transverse sections). **D**, Heart weight/body weight (HW/BW) and heart weight/tibia length (HW/TL) ratios (sham+CTR, n=7; sham+Sam68cKO, n=7; TAC+CTR, n=8; TAC+Sam68cKO, n=8). **E**, Wheat germ agglutinin (WGA) staining (left 4 panels) and cardiomyocyte cross-sectional area (right panel) (n=5/group). **F**, Picrosirius red staining (left 4 panels) and collagen volume fraction (right panel ) (n=5/group). **G** and **H**, Representative immunoblots (**G**) and quantification (**H**) of ANP (atrial natriuretic peptide) and BNP (brain natriuretic peptide) (sham, n=3/group; TAC, n=4/group). Molecular weight markers (kDa) are indicated on immunoblots. Data are mean±SEM. Two-way ANOVA with the Tukey multiple comparison test (**A**, **B**, **D**, **E**, **F**, and **H**). Sam68 indicates Src-associated in mitosis, 68 kDa.

To define the cardiomyocyte-autonomous role of Sam68 in pressure-overload remodeling, we generated tamoxifen-inducible cardiomyocyte-specific knockout mice (Sam68^flox/flox^;Myh6-MerCreMer [Sam68cKO])^22^ and littermate Cre controls (CTR; Figure S2A). Tamoxifen induction efficiently reduced Sam68 protein in Sam68cKO hearts (Figure S2B). CTR and Sam68cKO mice underwent a sham procedure or TAC and were followed for 4 weeks. Survival did not differ between genotypes (Figure S2C). At 2 weeks after TAC, Sam68cKO hearts already exhibited reduced interventricular septal thickness in diastole and systole (Figure S2D and S2E); ejection fraction (EF) and fractional shortening (FS) were similar (Figure S2F and Table S1). By 4 weeks, the protective phenotype was more pronounced: Sam68cKO hearts showed smaller LV internal diameter and improved systolic performance (higher EF and FS) compared with CTR-TAC hearts (Figure 2A and 2B and Table S2). Consistent with these functional improvements, Sam68cKO mice exhibited reduced hypertrophy indices (heart weight/body weight [HW/BW] and heart weight/tibia length [HW/TL]), decreased cardiomyocyte cross-sectional area, and reduced interstitial fibrosis (Figure 2C–2F). ANP (atrial natriuretic peptide) and BNP (brain natriuretic peptide) protein induction was also blunted (Figure 2G and 2H). Sam68cKO mice were similarly protected in an Ang II infusion model (Figure S3 and Table S3), supporting a consistent protective role for cardiomyocyte Sam68 loss across hypertrophic stimuli.

### Cardiomyocyte-Specific Sam68 Overexpression Exacerbates TAC-Induced Hypertrophy and Dysfunction

**Figure 3.**
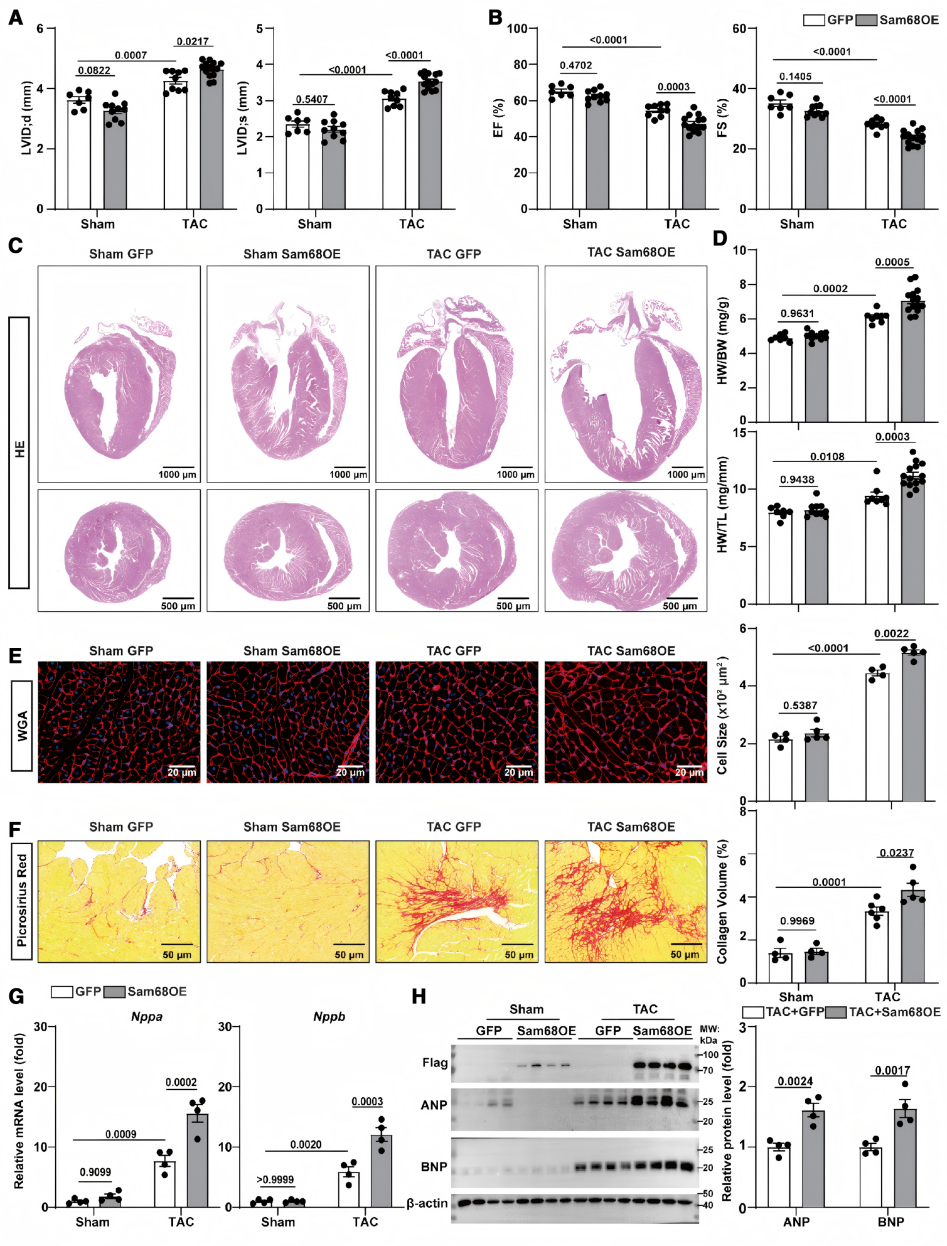
**Cardiomyocyte Sam68 overexpression exacerbates transverse aortic constriction–induced hypertrophy and dysfunction.** Mice received tail vein AAV9 (adeno-associated virus serotype 9)–cTnT (cardiac troponin T)–GFP (GFP) or AAV9–cTnT–Sam68–3×Flag (Sam68 [Src-associated in mitosis, 68 kDa] overexpression [Sam68OE]; 5×10^11^ vg/mouse) and underwent a sham procedure or transverse aortic constriction (TAC) 4 weeks later. Analysis was performed 4 weeks after surgery. **A** and **B**, Echocardiography results. **A**, Left ventricular internal diameter at end-diastole (LVID;d) and end-systole (LVID;s). **B**, Ejection fraction (EF) and fractional shortening (FS; group n as in Table S5). **C**, Representative hematoxylin & eosin (H&E) staining (longitudinal and transverse sections). **D**, Heart weight/body weight (HW/BW) and heart weight/tibia length (HW/TL) ratios (sham+GFP, n=7; sham+Sam68OE, n=10; TAC+GFP, n=8; TAC+Sam68OE, n=14). **E**, Wheat germ agglutinin (WGA) staining (left 4 panels) and cardiomyocyte cross-sectional area (right panel; sham+GFP, n=4; sham+Sam68OE, n=5; TAC+GFP, n=4; TAC+Sam68OE, n=5). **F**, Picrosirius red staining (left 4 panels) and collagen volume fraction (right panel) (sham+GFP, n=4; sham+Sam68OE, n=4; TAC+GFP, n=6; TAC+Sam68OE, n=5). **G**, *Nppa* and *Nppb* mRNA levels (n=4/group). **H**, Representative immunoblots (left) and quantification of ANP (atrial natriuretic peptide)/BNP (brain natriuretic peptide; right). Molecular weight markers (kDa) are indicated on immunoblots (n=4/group). Data are mean±SEM. Two-way ANOVA with the Tukey multiple comparison test (**A**, **B**, **D** through **G**) or with the Sidak multiple-comparisons test (**H**).

To test whether cardiomyocyte Sam68 upregulation is sufficient to worsen remodeling, we overexpressed Sam68 using AAV9 (adeno-associated virus serotype 9) under the cTnT (cardiac troponin T) promoter. Flag-tagged Sam68 (AAV9–cTnT–Sam68–3×Flag [Sam68OE]) or control AAV9–cTnT–GFP [GFP]) was delivered through tail vein injection (Figure S4A). Four weeks after delivery, Sam68 protein was robustly elevated in Sam68OE hearts (Figure S4B). After TAC, Sam68OE mice developed exaggerated hypertrophy at 2 weeks (Figure S4C and Table S4) and progressed to more severe systolic dysfunction and chamber dilation at 4 weeks (Figure 3A and 3B and Table S5). Sam68OE hearts exhibited increased HW/BW and HW/TL ratios, enlarged cardiomyocyte size, increased fibrosis, and heightened ANP/BNP expression (Figure 3C–3H). Thus, cardiomyocyte Sam68 upregulation is sufficient to amplify pressure-overload hypertrophy and accelerate dysfunction.

### Sam68 Deletion Preserves Oxidative Programs and Reshapes Pressure-Overload Metabolism

RNA-seq of Ang II–treated hearts revealed broad suppression of hypertrophy-associated programs and enrichment of oxidative phosphorylation (OXPHOS) pathways in Sam68cKO hearts (Figure S5A–S5C), consistent with reduced fetal/hypertrophy markers and increased electron transport chain gene expression (Figure S5D and S5E). Under TAC, Sam68 deletion restored representative mitochondrial complex subunits (eg, SDHB and ATP5a), whereas cardiomyocyte Sam68 overexpression further suppressed them (Figure S6A–S6D), supporting a role for Sam68 in limiting mitochondrial oxidative capacity during remodeling.

**Figure 4.**
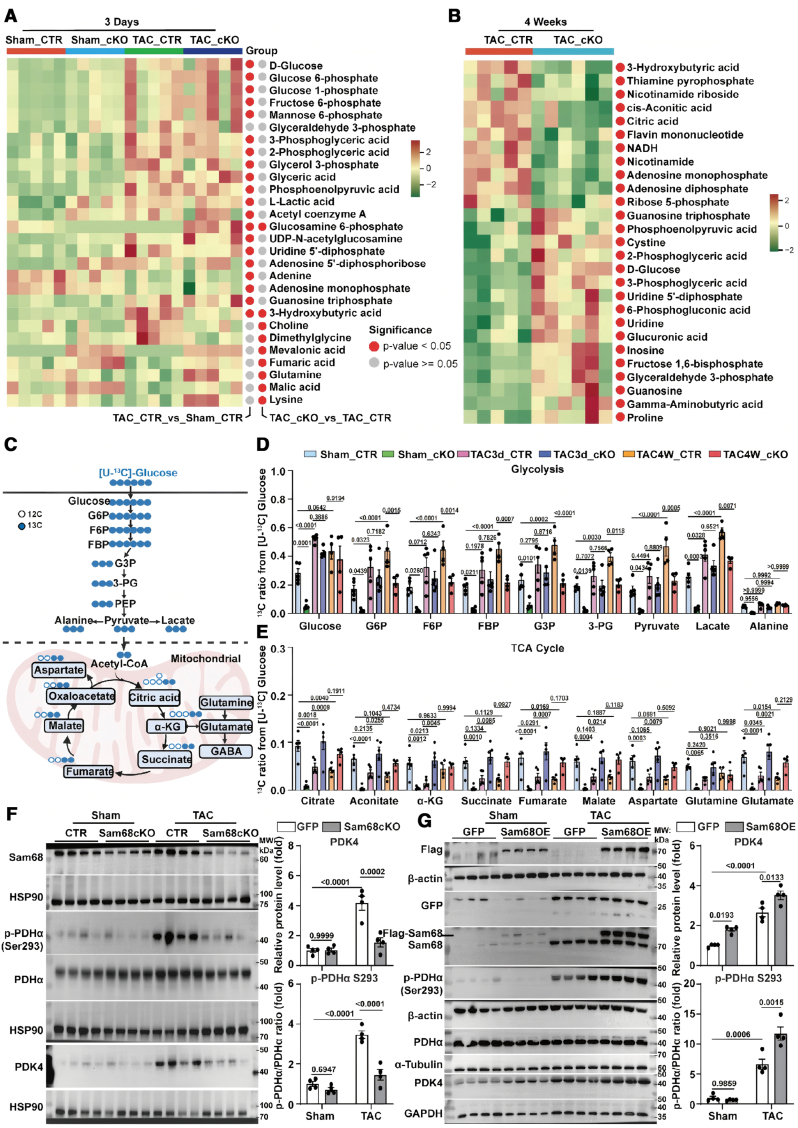
**Sam68 deletion promotes pyruvate oxidation and improves glucose-derived tricarboxylic acid entry during pressure overload. A** and **B**, Targeted energy-metabolome profiling in control (CTR) and cardiomyocyte-specific knockout (Sam68^flox/flox^;Myh6-MerCreMer [Sam68cKO]) hearts at 3 days (**A**) and 4 weeks (**B**) after a sham procedure or transverse aortic constriction (TAC). The heatmaps show differential metabolites (VIP>1 and *P*<0.05; n=5/group at day 3; at 4 weeks, n=5 CTR and n=6 Sam68cKO). **C**, Schematic of [U-^13^C]-glucose entry into glycolysis and the tricarboxylic acid (TCA) cycle and expected labeling patterns. **D** and **E**, Total ^13^C labeling of glycolysis-associated (**D**) and TCA cycle–associated (**E**) metabolites (including amino acids derived from these pathways), normalized to [U-^13^C]-glucose (sham, n=5/group; 3 days after TAC: n=5/group; 4 weeks after TAC: n=4/group). **F** and **G**, Representative immunoblots and quantification of PDK4 (pyruvate dehydrogenase kinase 4), p-PDHα(S293)/PDHα (pyruvate dehydrogenase E1 component subunit alpha), and total PDHα in CTR vs Sam68 cKO hearts (**F**) and GFP vs Sam68 (Src-associated in mitosis, 68 kDa) overexpression (Sam68OE) hearts (**G**) at 4 weeks after a sham procedure or TAC (n=4/group). Molecular weight markers (kDa) are indicated. Data are mean±SEM. Two-way ANOVA with the Tukey multiple comparison test (**D** through **G**). 3PG indicates 3-phosphoglycerate; α-KG, α-ketoglutarate; F6P, fructose-6-phosphate; FBP, fructose-1,6-bisphosphate; G3P, glyceraldehyde-3-phosphate; and G6P, glucose-6-phosphate.

To define metabolic remodeling during pressure overload, we performed targeted metabolomics in sham and TAC hearts at 3 days and 4 weeks. Profiling of 132 metabolites showed clear separation between groups at both time points, and Sam68 deletion significantly reshaped the TAC-induced metabolic response (Figure S7A–S7F). At 3 days after TAC, CTR hearts exhibited an early glycolytic/ketogenic signature, with accumulation of glycolytic intermediates, lactate, and ketone bodies (eg, 3-hydroxybutyrate). In contrast, Sam68cKO hearts showed a blunted ketogenic response and relative preservation of TCA-cycle intermediates (eg, fumarate and malate), consistent with earlier support of oxidative metabolism (Figure 4A). By 4 weeks, CTR hearts displayed features of energetic stress, including depletion of amino acids and glycolytic/gluconeogenic precursors with increased AMP (Figure S7G). Sam68cKO hearts exhibited a distinct chronic profile, with increased intermediates in upstream glucose-utilizing pathways (glycolysis and pentose phosphate pathway) and reduced levels of several downstream oxidative/redox-related metabolites (including select TCA intermediates, such as citrate), consistent with sustained reprogramming of substrate handling during chronic pressure overload (Figure 4B).

Collectively, transcriptomic profiling, ETC (electron transport chain) protein analyses, and targeted metabolomics indicate that Sam68 promotes maladaptive metabolic remodeling during hypertrophic stress, whereas cardiomyocyte-specific Sam68 deletion preserves oxidative programs and reshapes substrate utilization under pressure overload.

### Sam68 Promotes Pressure-Overload Uncoupling of Glycolysis from Glucose Oxidation

To directly assess pathway flux and test whether Sam68 enforces pressure-overload uncoupling of glycolysis from PDH-dependent mitochondrial glucose oxidation, we performed in vivo [U-^13^C]-glucose tracing to quantify the fate of glucose-derived carbon (Figure 4C). In TAC control hearts, glycolytic intermediates exhibited robust ^13^C labeling at both 3 days and 4 weeks, consistent with sustained activation of glycolysis under pressure overload (Figure 4D). In contrast, ^13^C incorporation into TCA cycle intermediates was significantly reduced at both time points, indicating persistent impairment of glucose-derived carbon entry into mitochondrial oxidation (Figure 4E). Cardiomyocyte-specific Sam68 deletion rescued this oxidative defect. At 3 days after TAC, Sam68cKO hearts showed a broad increase in ^13^C labeling across TCA cycle intermediates without further increasing glycolytic labeling (Figure 4D and 4E). By 4 weeks, Sam68 deletion maintained this improvement in TCA labeling and attenuated chronic hyperactivation of glycolysis, as reflected by reduced labeling across intermediates spanning glucose-6-phosphate through lactate (Figure 4D and 4E).

Isotopologue analysis clarified the entry routes of glucose-derived carbon. At 3 days after TAC, TAC control hearts displayed reduced M+2 labeling of TCA cycle intermediates, consistent with diminished PDH-mediated pyruvate entry. Sam68 deletion reversed this deficit, increasing M+2 labeling broadly and increasing M+3 labeling in select intermediates (citrate, glutamate, and glutamine), consistent with concurrent enhancement of oxidative entry and anaplerotic contribution during acute remodeling (Figure S7H). At 4 weeks, reduced M+2 labeling persisted in TAC control hearts, whereas Sam68cKO hearts exhibited a more selective but significant rescue (notably citrate, aconitate, and glutamate), accompanied by normalization of the M+3 labeling patterns (Figure S7H).

To integrate PDH entry versus anaplerosis, we analyzed the M+2/M+3 ratio. Under sham conditions, the high M+2/M+3 ratio confirmed PDH-dominant glucose oxidation over anaplerotic entry and was preserved in sham Sam68cKO hearts. At 3 days after TAC, Sam68 deletion increased both M+2 and M+3 labeling, yielding no major shift in the M+2/M+3 ratio. By 4 weeks, Sam68 deletion increased the M+2/M+3 ratio for glutamate (with similar upward trends in other intermediates), consistent with a relative shift toward PDH-dependent oxidative metabolism during prolonged stress (Figure S7I).

Together, these data show that Sam68 promotes pressure overload–induced uncoupling of glycolysis from mitochondrial glucose oxidation and identify Sam68 as a key regulator of PDH-dependent pyruvate oxidation during pathologic remodeling.

### Sam68 Is Required for Sustained PDK4 Induction and PDH Inhibition During Hypertrophic Stress

To define the molecular basis of impaired oxidation, we reanalyzed our 1-week Ang II RNA-seq data set. Among the most prominent changes was ≈3.5-fold downregulation of *Pdk4* (Figure S8A), encoding the kinase that phosphorylates and inhibits PDH-E1α. Single-cell data sets from failing human hearts showed that *SAM68* and *PDK4* transcripts are both increased in cardiomyocytes (Figure S8B), and a similar coexpression pattern was observed in TAC-stressed mouse cardiomyocytes (Figure S8C). RNA-seq of Ang II–infused hearts confirmed selective downregulation of *Pdk4* in Sam68cKO hearts compared with controls, with no comparable change in other *Pdk* isoforms (Figure S8D).

At the protein level, Sam68 deletion reduced PDK4 abundance and decreased the p-PDHα(S293)/PDHα ratio 4 weeks after TAC (Figure 4F), whereas Sam68 overexpression increased both measures (Figure 4G). PDH enzymatic activity paralleled these changes: TAC reduced PDH activity in controls, Sam68 deletion restored activity, and Sam68 overexpression further suppressed it (Figure S8E and S8F).

To establish temporal relationships, we performed a TAC time course of Sam68, PDK4, and p-PDHα(Ser293) (Figure S8G and S8H). Sam68 increased as early as day 1 after TAC and remained elevated (≈2-fold) through 4 weeks. PDK4 induction became evident by day 3 and persisted thereafter. p-PDHα(Ser293) showed a biphasic pattern, with an early rise at day 1, a decline by day 7, and a secondary rise by day 28. Notably, Sam68 induction precedes sustained PDK4 upregulation, supporting an upstream role for Sam68 in chronic activation of the PDK4/PDH inhibitory axis during pressure overload.

### Selective PDK4 Inhibition Reverses Sam68-Driven Remodeling

**Figure 5.**
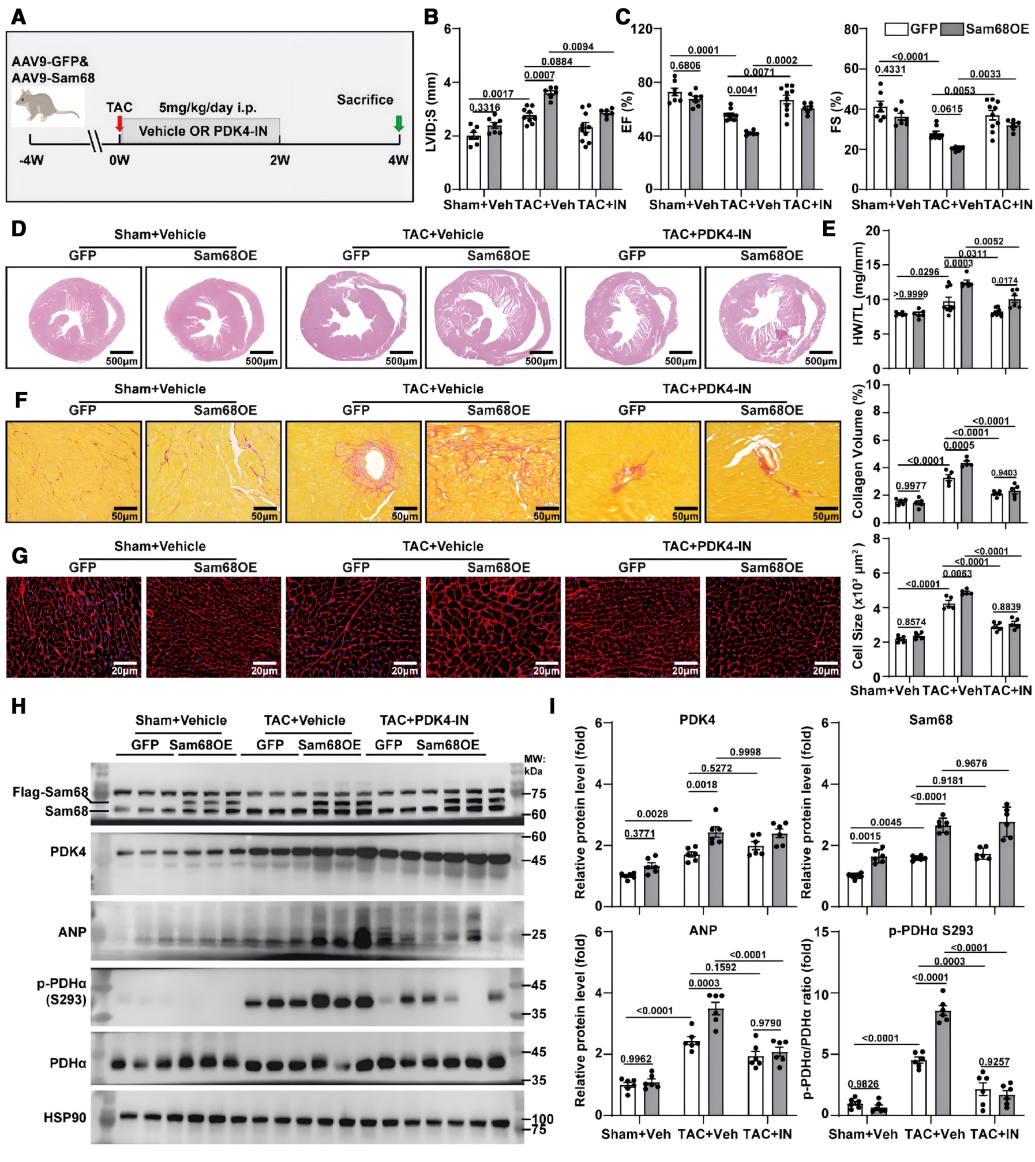
**Selective PDK4 inhibition reverses Sam68OE-driven remodeling and preserves PDH activity. A**, Study design: GFP and Sam68 (Src-associated in mitosis, 68 kDa) overexpression (Sam68OE) mice underwent a sham procedure or transverse aortic constriction (TAC) and were randomized to vehicle or PDK4 (pyruvate dehydrogenase kinase 4) inhibitor (5 mg/kg per day IP) for 2 weeks. **B** and **C**, Echocardiography results. **B**, Left ventricular internal diameter at end-systole (LVID;s). **C**, Ejection fraction (EF) and fractional shortening (FS; group n as in Table S6). **D,** Representative hematoxylin & eosin staining (cross-sections). **E**, Heart weight/tibia length (HW/TL) ratios (same n as **B**). **F**, Picrosirius red staining (left 4 panels) and collagen volume fraction (right panel; n=5/group). **G**, Wheat germ agglutinin staining (left 4 panels) and cardiomyocyte cross-sectional area (right panel; n=5/group). **H** and **I**, Representative immunoblots (**H**) and quantification (**I**) of PDK4, Sam68, ANP (atrial natriuretic peptide), and p-PDHα(S293)/PDHα (pyruvate dehydrogenase E1 component subunit alpha; n=6/group). Molecular weight markers (kDa) are indicated. Data are mean±SEM. Two-way ANOVA with the Tukey multiple comparison test (**B**, **C**, **E**, **F**, **G**, and **I**). AAV9 indicates adeno-associated virus serotype 9.

To test whether Sam68 exacerbates hypertrophy through PDK4-dependent PDH inhibition, we treated Sam68OE and GFP mice with the PDK4-selective inhibitor PDK4-IN (Compound 8C). Four weeks after AAV9 delivery, mice underwent a sham procedure or TAC and were immediately randomized to vehicle or PDK4-IN (5 mg/kg per day IP) for 2 weeks (Figure 5A). At 4 weeks after TAC, sham-operated mice showed preserved structure and function. Under TAC, Sam68 overexpression increased LV internal diameter and further reduced EF and FS in vehicle-treated mice; PDK4-IN significantly attenuated these changes in both genotypes (Figure 5B and 5C, Figure S9A, and Table S6). PDK4-IN also reduced TAC-induced increases in heart size, HW/TL and HW/BW ratios, fibrosis, and cardiomyocyte cross-sectional area, including in Sam68OE mice (Figure 5D–5G and Figure S9B). At the molecular level, PDK4-IN reduced PDHα Ser293 phosphorylation and ANP induction without altering Sam68 or PDK4 protein abundance (Figure 5H and 5I), preserved PDH activity (Figure S9C), and maintained OXPHOS complex protein expression in TAC-treated GFP and Sam68OE hearts (Figure S9D and S9E). Together, these results support PDK4-mediated PDH inhibition as a key downstream effector of Sam68 and show that selective PDK4 inhibition counteracts Sam68-driven remodeling under pressure overload.

### Sam68 Scaffolds a Src–STAT3 Module to Induce PDK4

To identify upstream mediators linking Sam68 to PDK4 induction, we screened candidate Sam68-interacting partners. STRING network analysis highlighted STAT3—a transcription factor previously implicated in PDK4 regulation^28^—as a putative interactor (Figure S10A). Protein–protein docking (HDOCK) further supported a direct Sam68–STAT3 interface (Figure S10B). We validated this interaction experimentally by coimmunoprecipitation in Ang II–stimulated NRVMs and in Sam68OE hearts (Figure S10C and S10D). Consistent with functional coupling, transcriptomic profiling revealed altered STAT3-responsive gene programs in Sam68-deficient hearts^29^ (Figure S10E).

**Figure 6.**
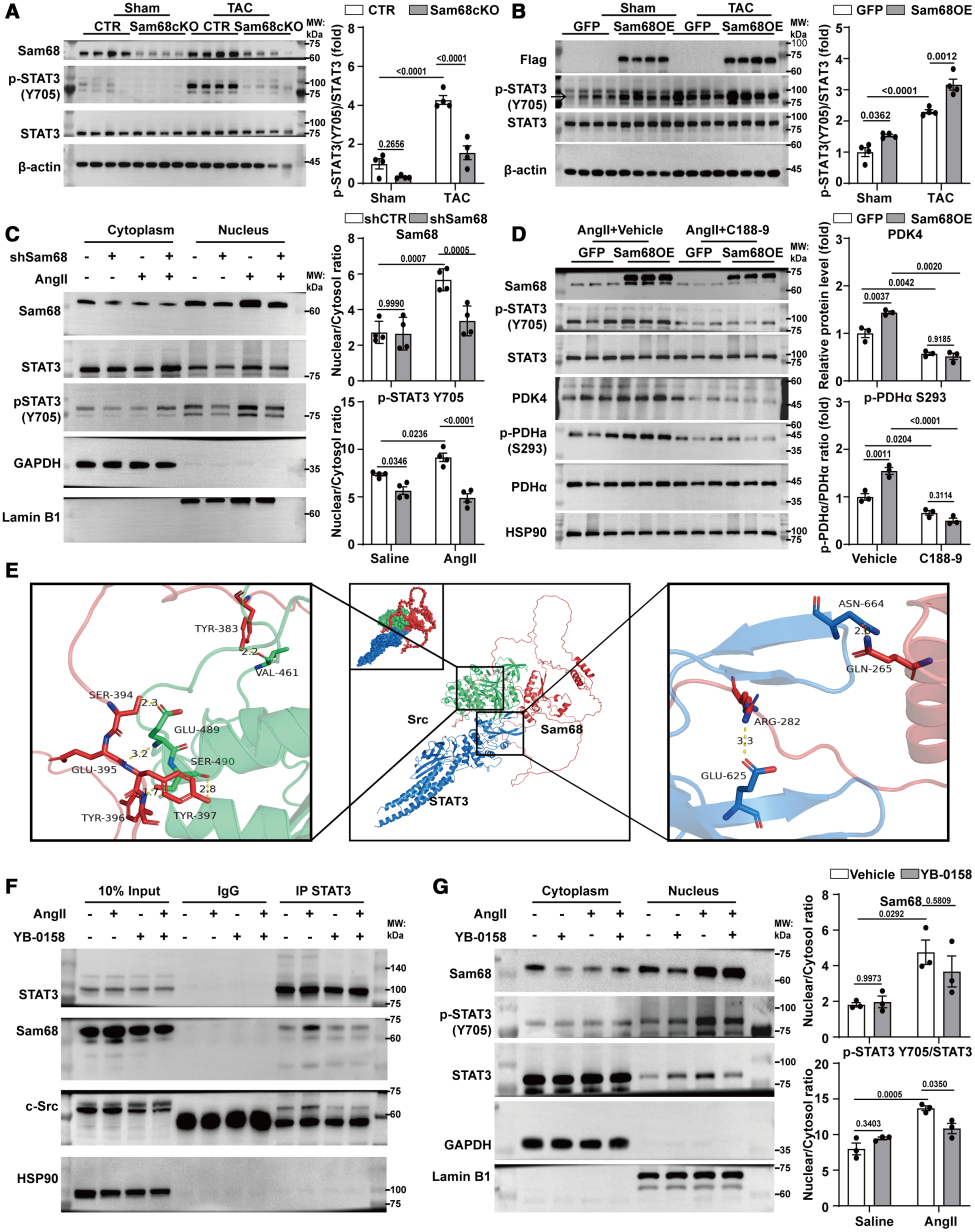
**Sam68 scaffolds Src–STAT3 signaling to drive STAT3 Tyr705 phosphorylation, nuclear accumulation, and PDK4 induction. A**, Representative immunoblots and quantification of p-STAT3(Y705) and total STAT3 (signal transducer and activator of transcription 3) in control (CTR) versus cardiomyocyte-specific knockout (Sam68^flox/flox^;Myh6-MerCreMer [Sam68cKO]) mouse hearts 4 weeks after a sham procedure or transverse aortic constriction (TAC). **B**, The same phosphoproteins in GFP versus Sam68 (Src-associated in mitosis, 68 kDa) overexpression (Sam68OE) hearts 4 weeks after a sham procedure or TAC (n=4/group). **C**, Neonatal rat ventricular myocytes transduced with lentiviral Sam68-shRNA or control were stimulated with angiotensin II (Ang II; 1 μM, 24 hours); cytoplasmic and nuclear fractions were immunoblotted and nuclear:cytoplasmic ratios quantified. **D**, Neonatal rat ventricular myocytes transduced with adenovirus GFP or Sam68OE were treated with Ang II±C188-9 (10 µM, 24 hours); immunoblots for Sam68, p-STAT3(Y705), STAT3, PDK4 (pyruvate dehydrogenase kinase 4), and p-PDHα(S293)/PDHα (pyruvate dehydrogenase E1 component subunit alpha) with quantification. **E**, Representative docking snapshots of Src–Sam68–STAT3 interfaces. **F**, STAT3 immunoprecipitation from neonatal rat ventricular myocytes treated with Ang II±YB-0158 (1 µM) and immunoblot for coprecipitated Src and Sam68. **G**, Neonatal rat ventricular myocytes treated with vehicle or YB-0158±Ang II for 24 hours were fractionated; nuclear:cytoplasmic ratios of Sam68 and p-STAT3(Y705) were quantified. Molecular weight markers (kDa) are indicated. Data are mean±SEM. Two-way ANOVA with the Tukey multiple comparison test (**A** through **D**, and **G**).

We next examined whether Sam68 regulates STAT3 activation and subcellular signaling. Canonical STAT3 activation requires Tyr705 phosphorylation, which promotes dimerization and nuclear import,^30^ whereas Ser727 phosphorylation has been linked to mitochondrial STAT3 signaling that supports OXPHOS.^31^ In vivo, TAC robustly increased p-STAT3(Y705) in control hearts, but this induction was markedly blunted in Sam68cKO hearts (Figure 6A). On the contrary, cardiomyocyte Sam68 overexpression elevated basal p-STAT3(Y705) in sham hearts and further potentiated its induction after TAC (Figure 6B). In NRVMs, Sam68 knockdown reduced Ang II–induced nuclear accumulation of p-STAT3(Y705) (Figure 6C). Pharmacologic STAT3 inhibition with C188-9 abolished Sam68-dependent PDK4 upregulation and PDHα Ser293 phosphorylation in Ang II–stimulated NRVMs (Figure 6D), establishing STAT3 as a required mediator of the Sam68–PDK4/PDH axis. Notably, although Sam68 knockdown preserved total mitochondrial STAT3 abundance under stress, it did not restore mitochondrial p-STAT3(S727) (Figure S10F and S10G), suggesting that Sam68 primarily promotes pathologic remodeling through Tyr705-dependent nuclear STAT3 signaling rather than through Ser727-associated mitochondrial STAT3 activity.

Because Sam68 is a known Src substrate and scaffold, we tested whether Sam68 couples STAT3 to Src. Docking supported a Src–Sam68–STAT3 complex (Figure 6E). Experimental Sam68 knockdown or Src inhibition each blocked Ang II–induced STAT3 Tyr705 phosphorylation without additive effects, consistent with Sam68 acting as the key adaptor enabling Src-dependent STAT3 activation (Figure S11A). Disrupting the Sam68–Src interface using the peptidomimetic inhibitor YB-0158^32^ inhibited Sam68–Src binding (Figure S11B), prevented Src–STAT3 complex formation (Figure 6F), and suppressed nuclear STAT3 activation (Figure 6G). Together, these data demonstrate that Sam68 organizes a Src–STAT3 signaling module that drives PDK4 transcription, PDH inhibition, and impaired pyruvate oxidation during hypertrophic stress.

### A STAT3–Sam68 Positive Feedback Loop Amplifies the Pathogenic Circuit

To identify transcriptional drivers of Sam68 upregulation, we performed an integrative bioinformatic screen (ChIP-Atlas, CHEA, KnockTF, hTFtarget, GTRD, JASPAR, and ENCODE). STAT3 emerged as a top predicted regulator of Sam68 and 2 conserved STAT3 motifs were identified in the Sam68 promoter (Figure S12A and S12B). Public chromatin immunoprecipitation followed by sequencing data (GSE85579)^33^ supported STAT3 occupancy (Figure S12C), and dual-luciferase reporter assays confirmed STAT3-dependent promoter activation (Figure S12D). In NRVMs, C188-9 reduced basal Sam68 expression and blunted Ang II–induced Sam68 upregulation, accompanied by reduced PDK4 and p-PDH-E1α(S293) (Figure S12E and S12F). These data identify a positive feedback loop in which Sam68 activates STAT3 and STAT3 directly enhances Sam68 transcription, reinforcing the Src–Sam68–STAT3–PDK4 circuit during stress.

### YB-0158 Engages Sam68 in the Heart and Attenuates TAC Remodeling in a Sam68-Dependent Manner

To determine whether YB-0158 confers cardioprotection through Sam68 inhibition, we first characterized pharmacokinetics and target engagement. After a single IP injection, YB-0158 showed dose-dependent exposure in plasma and heart. At 5 mg/kg, plasma C_max_ was 220 ng/mL with AUC_0–t_ 146 h·ng/mL; cardiac exposure reached C_max_ of 28.5 ng/g with AUC_0–t_ of 36.8 h·ng/g, and exposure approximately doubled at 10 mg/kg (Figure S13A and S13B). A single 5-mg/kg dose administered 1 day after TAC rapidly inhibited the Sam68–Src interaction within 2 hours and sustained inhibition for up to 24 hours; it also reduced Sam68–STAT3 interaction and suppressed p-STAT3(Y705) from 6 to 24 hours (Figure S13C and S13D).

We next tested YB-0158 in vivo using a preventive regimen (5 mg/kg per day, IP, starting 3 days before surgery and continuing through day 7 after surgery; Figure S14A). At 4 weeks after TAC, YB-0158 significantly attenuated remodeling and dysfunction, improving systolic performance (reduced LV internal diameter at end-systole with increased EF and FS), reducing hypertrophy indices (HW/BW and HW/TL), and limiting fibrosis, cardiomyocyte enlargement, and ANP/BNP induction (Figure S14B–S14G and Table S7). Notably, this protective profile closely phenocopied the Sam68cKO response to pressure overload.

**Figure 7.**
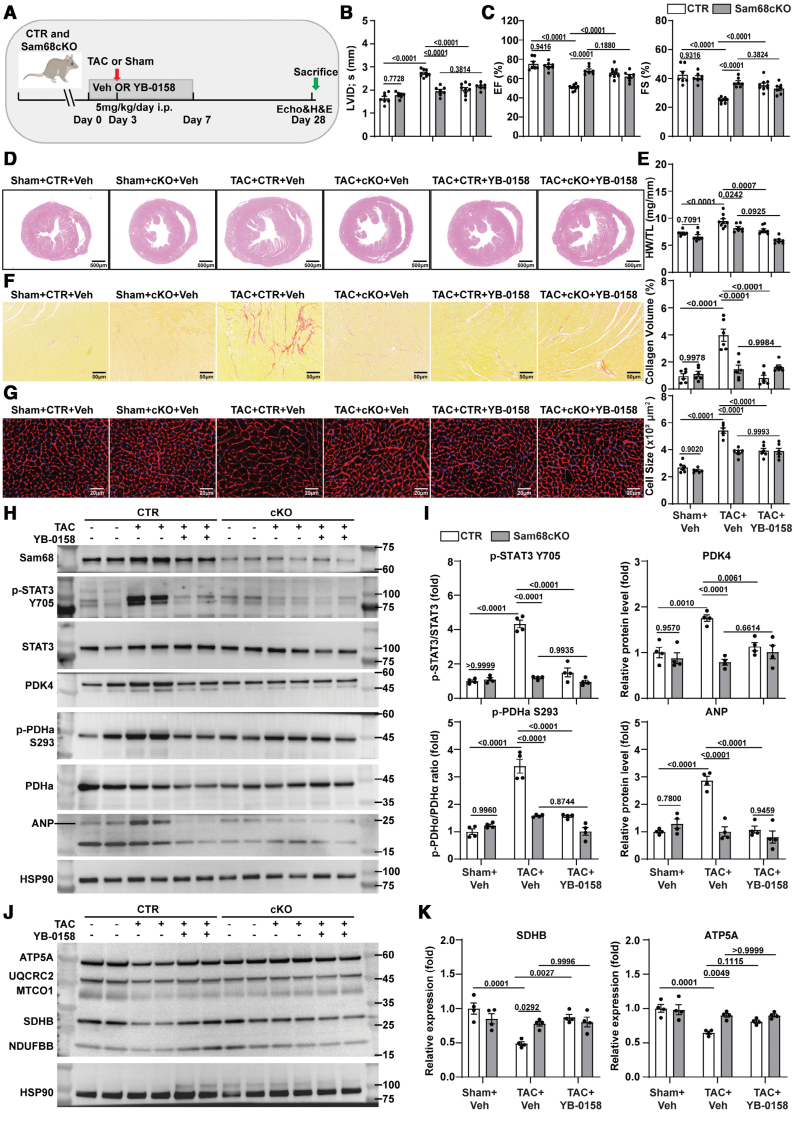
**Pharmacologic disruption of the Src–Sam68 interface limits TAC remodeling in a Sam68-dependent manner. A**, Study design: control (CTR) and cardiomyocyte-specific knockout (Sam68^flox/flox^;Myh6-MerCreMer [Sam68cKO]) mice received vehicle or YB-0158, underwent a sham procedure or transverse aortic constriction (TAC), and were assessed at 4 weeks after surgery. **B** and **C**, Echocardiography results. **B**, Left ventricular internal diameter at end-systole (LVID;s). **C**, Ejection fraction (EF) and fractional shortening (FS). **D**, Representative hematoxylin & eosin staining (cross-sections). **E**, Heart weight/tibia length (HW/TL) ratio (group n as indicated). **F**, Picrosirius red staining (left 4 panels) and collagen volume fraction (right panels; n=6/group). **G**, Wheat germ agglutinin staining (left 4 panels) and cardiomyocyte cross-sectional area (right panels; n=6/group). **H** and **I**, Representative immunoblots (**H**) and quantification (**I**) of p-STAT3(Y705)/STAT3 (signal transducer and activator of transcription 3), PDK4 (pyruvate dehydrogenase kinase 4), p-PDHα(S293)/PDHα (pyruvate dehydrogenase E1 component subunit alpha), and ANP (atrial natriuretic peptide; n=4/group). **J** and **K**, Immunoblots (**J**) and quantification (**K**) of representative electron transport chain/oxidative phosphorylation subunits (SDHB [succinate dehydrogenase complex iron-sulfur subunit B], ATP5a [alpha subunit of the catalytic core of mitochondrial ATP synthase]; n=4/group). Molecular weight markers (kDa) are indicated. Data are mean±SEM. Two-way ANOVA with the Tukey multiple comparison test (**B** and **C**, **E** through **G**, and **K**). Sam68 indicates Src-associated in mitosis, 68 kDa.

To test on-target dependence, CTR and Sam68cKO mice were treated with YB-0158 after TAC (Figure 7A). YB-0158 conferred robust protection in CTR mice but provided minimal additional benefit in Sam68cKO mice (Figure 7B–7G and Table S8), supporting Sam68-dependent efficacy. Consistently, YB-0158 suppressed TAC-induced activation of the Sam68–STAT3–PDK4 axis (p-STAT3[Y705], PDK4, p-PDHα[S293], and ANP) and restored representative OXPHOS subunits (SDHB and ATP5a) in CTR hearts, with little effect in Sam68cKO hearts (Figure 7H–7K). These data collectively indicate that YB-0158 limits pressure-overload remodeling primarily through Sam68-dependent inhibition of the maladaptive STAT3–PDK4/PDH pathway.

### The Src–SAM68–STAT3–PDK4 Axis Is Activated in Human Failing Hearts

**Figure 8.**
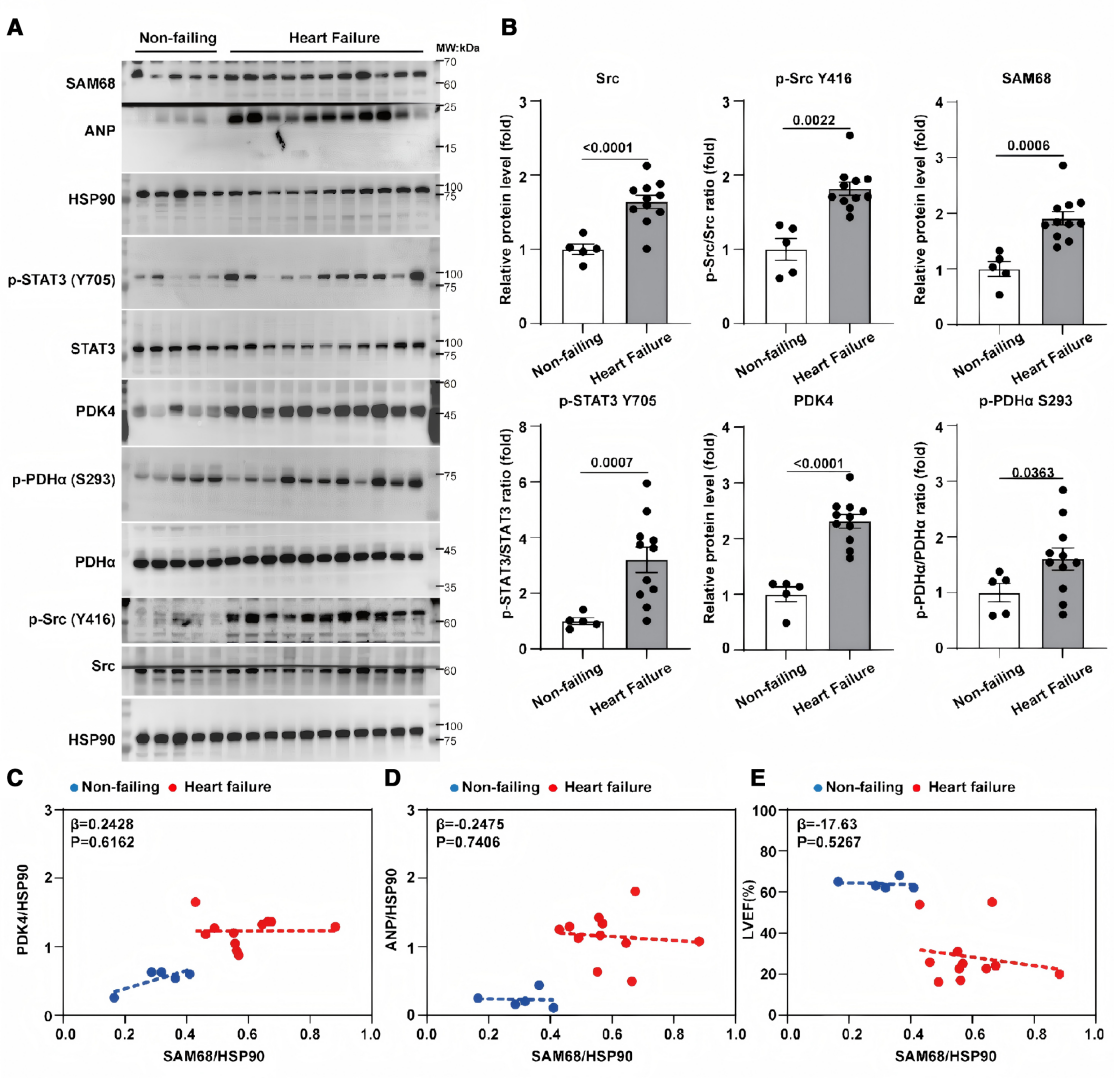
**The Src–SAM68–STAT3–PDK4 axis is activated in failing human hearts. A**, Representative immunoblots of left ventricular tissue from nonfailing and heart failure samples, probed for SAM68 (Src-associated in mitosis, 68 kDa), p-STAT3(Tyr705), STAT3 (signal transducer and activator of transcription 3), p-PDHα(S293), PDHα (pyruvate dehydrogenase E1 component subunit alpha), PDK4 (pyruvate dehydrogenase kinase 4), Src, p-Src(Tyr416), and ANP (atrial natriuretic peptide). **B**, Quantification of SAM68, PDK4, Src, and the indicated phosphorylation ratios (nonfailing, n=5; heart failure, n=11). **C** through **E**, Scatterplots showing SAM68 protein levels (relative to HSP90) versus (**C**) PDK4 protein, (**D**) ANP protein, and (**E**) left ventricular ejection fraction (%) in nonfailing (blue dots, n=5) and heart failure (red dots, n=11) human left ventricular samples. Dashed lines show group-specific fitted lines for visualization only. β coefficients and *P* values were derived from simple linear regression models adjusted for group status. Data are mean±SEM. Unpaired 2-tailed Student *t* test (**B**, Src, p-Src[Tyr416], SAM68, PDK4, p-PDHα[Ser293]), Welch *t* test (**B**, p-STAT3[Tyr705]), and simple linear regression adjusting for group status (**C** through **E**).

In human LV tissue (Table S9), p-Src(Y416), SAM68, p-STAT3(Y705), PDK4, p-PDHα(S293), and ANP were markedly increased in failing hearts compared with nonfailing controls (Figure 8A and 8B), consistent with activation of the Src–SAM68–STAT3–PDK4 signaling axis and inhibition of PDH in human HF.

When we examined the relationship between SAM68 protein abundance and functional measures, Sam68 levels were elevated in parallel with PDK4 and ANP and in the context of reduced LVEF (Figure 8C–8E). However, after adjusting for HF status using simple linear regression, Sam68 was not independently associated with PDK4 (β=0.24, *P*=0.62), ANP (β=−0.25, *P*=0.74), or LVEF (β=−17.6, *P* = 0.52; Figure 8C–8E). These findings indicate that the relationships observed in pooled analyses are primarily driven by disease state rather than continuous variation in Sam68 protein levels within this cohort.

Together with our experimental data identifying Sam68 as a stress-induced scaffold that facilitates Src-dependent STAT3 activation, these human data support a model in which Sam68 functions as an upstream regulator of the Src–STAT3–PDK4 axis in the failing heart.

## Discussion

Altered cardiac energetics is a fundamental driver of hypertrophy and HF, yet the upstream mechanisms that enforce impaired glucose oxidation during pressure overload have remained incompletely defined.^34^ Here, we identify Sam68—markedly upregulated in failing human hearts and in pressure-overloaded mouse hearts—as a key stress-responsive regulator of this maladaptive metabolic switch. Mechanistically, Sam68 functions as a signaling scaffold that couples activated Src to STAT3, enabling Src-dependent STAT3 Tyr705 phosphorylation, nuclear accumulation, and transcriptional induction of PDK4. Increased PDK4 then sustains PDH inhibition, suppresses pyruvate oxidation, and accelerates maladaptive remodeling. Importantly, cardiomyocyte-specific Sam68 deletion and pharmacologic disruption of the Src–Sam68 interface (YB-0158) restore PDH activity, improve mitochondrial energetic programs, and attenuate pressure-overload hypertrophy and dysfunction (Figure S15). Together, these data establish Sam68 as a stress-activated metabolic regulator that drives pathologic hypertrophy by suppressing cardiomyocyte glucose oxidation and identify Sam68 as a druggable control point for metabolic rescue.

A notable finding from our in vivo flux analyses is that Sam68cKO hearts exhibit reduced ^13^C-glucose utilization under sham conditions. This phenotype is consistent with previous work implicating Sam68 in AKT signaling and glucose uptake.^23,35^ Importantly, despite lower glucose-derived carbon influx, glycolytic intermediate pools were not depleted, and the M+2/M+3 ratio was preserved in sham Sam68cKO hearts, indicating that the intrinsic balance between PDH-mediated oxidation and anaplerotic entry remains intact under physiologic conditions. These data support a context-dependent model: Sam68 contributes to basal glucose utilization without materially shifting oxidative–anaplerotic coupling, whereas hypertrophic stress induces Sam68 and converts it into an active driver of maladaptive metabolic remodeling.

Under pressure overload, our data support a central role for Sam68 in enforcing a PDK4-dependent brake on pyruvate oxidation. Pressure overload robustly increases PDK4 in control hearts, whereas cardiomyocyte Sam68 deletion abolishes this induction. This relationship is supported across species and platforms, including human data sets and TAC-stressed mouse cardiomyocytes.^12,15^ Our longitudinal profiling adds temporal resolution: Sam68 is induced early (day 1) and precedes sustained PDK4 upregulation (day 3 onward), supporting an upstream role for Sam68 in chronic activation of the PDK4/PDH inhibitory axis. The biphasic pattern of PDH Ser293 phosphorylation further suggests that early PDH regulation reflects acute stress inputs or phosphatase dynamics, whereas late sustained PDH inhibition aligns with persistent Sam68/PDK4 elevation during chronic remodeling.

These mechanistic relationships have direct consequences for remodeling beyond metabolism alone. Sustained PDK4 activity is not merely a metabolic brake; chronic suppression of pyruvate oxidation has been linked to impaired excitation–contraction coupling and activation of fetal/hypertrophic gene programs.^36,37^ Consistent with this, selective PDK4 inhibition in Sam68OE hearts preserved PDH activity and mitigated the exaggerated remodeling phenotype, establishing PDK4-mediated PDH inhibition as a functional downstream effector of Sam68-driven disease progression. PDK4 inhibition also improved mitochondrial protein expression in TAC-treated control hearts, indicating that PDK4 broadly contributes to pressure-overload metabolic dysfunction while mediating the additional burden imposed by Sam68 upregulation. Although broad PDK inhibitors (eg, dichloroacetate) have toxicity and efficacy limitations,^38,39^ our data identify Sam68—and the Src–Sam68–STAT3–PDK4 pathway it controls—as an alternative, mechanistically specific entry point to relieve PDH inhibition.

This mechanism is especially compelling in the broader metabolic context of hypertrophy. Early hypertrophy features declining FAO alongside induction of PDK4, effectively impairing oxidative ATP production from both major substrates: fatty acids (reduced FAO) and glucose (PDH inhibition). In this setting, strategies that shift substrate preference toward glucose are likely to achieve maximal benefit only if PDH-dependent pyruvate oxidation is simultaneously restored, thereby channeling increased glycolytic carbon into the TCA cycle and improving energetic efficiency (ATP per O_2_). Partial FAO inhibition (eg, ninerafaxstat) has been reported to improve exercise capacity in nonobstructive hypertrophic cardiomyopathy, consistent with pushing substrate use toward glucose.^40^ Our results predict that relieving the PDK4/PDH block would “unlock” the energetic benefit of such a glucose shift by increasing mitochondrial pyruvate oxidation. Accordingly, the Sam68–Src–STAT3–PDK4 axis offers multiple therapeutic entry points: disrupting the Sam68–Src interface to prevent pathologic STAT3 activation, limiting STAT3-dependent PDK4 transcription, or applying isoform-selective PDK4 inhibitors to directly restore PDH activity.

Our findings also refine the therapeutic interpretation of STAT3. STAT3 signaling requires tight balance: sustained p-STAT3(Y705) promotes maladaptive transcription and inflammation, whereas excessive suppression can impair vascular integrity and exacerbate fibrosis.^41,42^ This dualism is shaped in part by site-specific phosphorylation: p-STAT3(Y705) predominantly drives nuclear gene programs linked to pathologic remodeling,^29,43^ whereas p-STAT3(S727) has been associated with mitochondrial STAT3 functions that support ATP synthesis and limit ROS.^31,44^ Therefore, indiscriminate STAT3 inhibition may be hazardous.^41–43^ In this context, targeting Sam68 offers a more selective leverage point. Sam68 loss suppresses the maladaptive nuclear p-STAT3(Y705)–PDK4 program but does not prevent the stress-associated decline in mitochondrial p-STAT3(S727), despite preserving total mitochondrial STAT3 abundance. Because Ser727 phosphorylation is required for the OXPHOS-supportive activity of mitochondrial STAT3,^31,45^ these data argue that restoration of Ser727-dependent mitochondrial STAT3 function is unlikely to be the dominant protective mechanism in our model. Instead, the primary benefit of Sam68 perturbation appears to be selective suppression of the prohypertrophic nuclear STAT3 program that drives PDK4 induction and PDH inhibition.

From a translational perspective, reverse-turn peptidomimetics have emerged as Sam68-directed agents.^46^ YB-0158 exhibits high affinity for Sam68 and disrupts the Sam68–Src interaction in cancer models.^32^ In our study, YB-0158 attenuated pathologic remodeling induced by Ang II infusion or TAC, demonstrated favorable plasma and cardiac exposure, produced robust cardiac target engagement, and showed Sam68-dependent on-target efficacy without overt toxicity in major organs or measurable changes in behavioral or routine clinical indices in treated mice. These findings highlight the therapeutic potential of pharmacologically targeting Sam68 in pressure-overload hypertrophy. Nevertheless, comprehensive evaluation of long-term safety, pharmacodynamics, and off-target liabilities is required before translation. It is also notable that CWP-291, another reported Sam68-targeting agent, has entered early-phase clinical trials for hematologic malignancies (Clinical Study of CWP232291 in Relapsed or Refractory Myeloma Patients [URL: https://www.clinicaltrials.gov; Unique identifier: NCT02426723] and Clinical Study of CWP232291 in Acute Myeloid Leukemia Patients [URL: https://www.clinicaltrials.gov; Unique identifier: NCT03055286]), although its structural basis and mode of Sam68 engagement have not been disclosed.^47^ Future studies should assess whether CWP-291 or related chemotypes modulate cardiac remodeling and how their mechanisms relate to YB-0158.

In the human failing heart cohort, Sam68 protein abundance was elevated in parallel with PDK4 and ANP and in the context of reduced LVEF; however, after controlling for disease status, Sam68 did not exhibit an independent linear association with these measures. This pattern suggests that the observed relationships reflect disease state differences rather than graded variation within the failing group. However, this does not argue against a pathogenic role for Sam68. Our experimental data demonstrate that Sam68 functions as a stress-induced signaling scaffold that facilitates Src-dependent STAT3 activation. Genetic deletion of Sam68 attenuates PDK4 induction and cardiac remodeling, whereas cardiomyocyte-specific overexpression exacerbates these phenotypes. Together, these findings support a model in which Sam68 operates as an upstream regulator of the Src–STAT3–PDK4 axis, with disease-associated upregulation sufficient to engage downstream metabolic remodeling. In this context, the absence of an independent linear association in the clinical cohort is consistent with Sam68 acting as a disease-linked regulatory node rather than as a graded determinant of HF severity.

This study has several limitations that define priorities for future investigation. First, cardiomyocyte-specific Sam68 deletion reduced basal cardiac glucose utilization, but the underlying mechanism and physiologic consequence remain unclear. Although previous studies have implicated Sam68 in insulin–AKT signaling,^23,35^ this has not been directly established in cardiomyocytes, and whether reduced basal glucose utilization alters responses to other forms of stress remains unknown. Second, although Sam68 deletion preserved total mitochondrial STAT3 under Ang II stimulation, it did not restore activating Ser727 phosphorylation. The functional significance of this preserved but hypophosphorylated mitochondrial STAT3 pool, as well as the mechanisms regulating mitochondrial STAT3 abundance or stability, therefore remains unresolved. Compartment-restricted STAT3 tools, including nucleus-restricted or mitochondria-targeted STAT3 variants, together with direct assessment of mitochondrial function, will be important to define compartment-specific roles. Third, the current study was performed exclusively in male mice. Sex is a major biologic variable in pressure overload–induced cardiac remodeling, and female mice generally exhibit a less pronounced hypertrophic and fibrotic response, in part because of estrogen-mediated cardioprotection.^48^ Although this rationale supported the use of a male model in the current mechanistic study, whether Sam68 exerts sex-specific effects under pathologic stress warrants further investigation. Fourth, although our findings support Sam68 as a druggable node, therapeutic targeting remains at an early stage. Rigorous long-term evaluation of safety, pharmacodynamics, and off-target effects will be required, and extended models of cardiac remodeling and vascular disease will be needed to define tissue-specific benefits and liabilities.

Our results identify Sam68 as a stress-activated regulator that drives pathologic hypertrophy, at least in part, by inducing a Src–STAT3–PDK4 program that inhibits PDH and suppresses pyruvate oxidation. These findings establish Sam68 as a previously unrecognized druggable control point in cardiac metabolic remodeling and support targeting the Src–Sam68–STAT3–PDK4 axis to slow progression from hypertrophy to HF.

## ARTICLE INFORMATION

### Acknowledgments

The authors thank the Gene Denovo Corporation for support in bioinformatics analysis through the Omicshare Cloud Analysis Platform.

### Author Contributions

Dr An designed and conducted the experiments, analyzed the data, and wrote the manuscript. Dr Han conducted the experiments and revised the manuscript. Drs Jiang, Shi, and Xu and J. Huang conducted the experiments and analyzed the data. C. Wang performed the bioinformatics analyses. J. Ni and Dr Cao provided technical support for metabolic flux analysis. Drs Feng and Lyu provided technical assistance and advice. Drs Li and Dong provided key research materials. Dr Qin conceived and designed the experiments, analyzed the data, and revised the manuscript. All authors approved the final version of the manuscript.

### Disclosures

None.

### Supplemental Material

Checklist

Methods

Tables S1–S11

Figures S1–S15

Excel File S1

References 49–53

## Supplementary Material
