# Supplementary material for: Sam68 Exacerbates Pathologic Cardiac Hypertrophy by Suppressing Cardiomyocyte Glucose Oxidation
Source: Circulation. 2026 May 22;153(25):2044–63. doi: 10.1161/CIRCULATIONAHA.125.077533 (PMC13286121; doi:10.1161/CIRCULATIONAHA.125.077533)
Supplement: Supplementary file 1 [file cir-153-2044-s001.pdf]

SUPPLEMENTAL MATERIAL

Sam68 Exacerbates Pathological Cardiac Hypertrophy by Suppressing Cardiomyocyte Glucose Oxidation

Junqing An, PhD[1,#], Chaoshan Han, PhD[1,#], Ying Jiang, MD[2], Jiawei Shi, MD, PhD[3], Huadong Li, MD[3], Chenqi Wang, MS[1], Jianrong Huang, MS[1], Shiyue Xu, MD[4], Jie Ni, MS[1], Yangpo Cao, PhD[1], Yuliang Feng, MD, PhD[1], Qing Lv, PhD[5], Nianguo Dong, MD, PhD[3], and Gangjian Qin, MD[1,*]

Short title: Sam68 Suppresses Glucose Oxidation in Hypertrophy

Expanded Methods

Ethics and Experimental animals

All animal experiments were approved by the Institutional Animal Care and Use Committee (IACUC) of the Southern University of Science and Technology and were conducted in accordance with the ARRIVE guidelines. Male C57BL/6J mice (10–12 weeks old) were randomly assigned to experimental groups with balanced age and body weight. Group allocation was performed by a researcher not involved in experimental procedures or data analysis, and outcome assessments were performed in a blinded manner. Sample sizes were determined based on prior studies. Animals that did not survive to the planned endpoint were unavailable for terminal assessments and therefore were not included in endpoint-specific analyses. Treatments and measurements were performed in a random order when feasible. Unless otherwise specified, *n* indicates the number of animals; detailed *n* values are provided in each figure legend. The experimental unit for in vivo studies was a single animal.

Mouse Lines and Genetic Models

Sam68-floxed mice were initially generated in our laboratory [22] and subsequently deposited at The Jackson Laboratory (stock no. 037100; Bar Harbor, ME, USA). To generate inducible cardiomyocyte-specific Sam68 knockout mice (Sam68cKO), Sam68-floxed mice (JAX #037100) were crossed with α-MHC–MerCreMer transgenic mice (Cyagen Biosciences, stock #C001443) to obtain Sam68$^{flox/flox}$;α-MHC–MerCreMer offspring. Cardiomyocyte-specific recombination was induced by tamoxifen (20 mg/kg/day, i.p.; T5648, Sigma-Aldrich) administered for five consecutive days. Littermate α-MHC–MerCreMer mice receiving the same tamoxifen regimen served as controls (CTR), unless otherwise stated.

AAV9-Mediated Cardiomyocyte Sam68 Overexpression

For cardiomyocyte-restricted Sam68 overexpression (Sam68-OE), *Sam68* or *GFP* was subcloned into a pAAV9-P2A vector under the cardiac troponin T (*cTnT*) promoter. Plasmids were packaged into AAV9, concentrated, and titered by qPCR. Eight-week-old C57BL/6J mice received a single tail-vein injection of 5 × 10^{11} vg/mouse in 100 μL to generate Sam68-OE or GFP-control mice. Four weeks later, mice were subjected to experiments.

Husbandry and Drug Treatments

Mice were housed under specific pathogen-free conditions on a 12-h light/dark cycle with ad libitum access to sterilized water and chow. In the PDK4 inhibitor study, GFP or Sam68OE mice received daily i.p. injections of PDK4-IN-1 (5 mg/kg/day; MedChemExpress) or vehicle (2% DMSO in saline) for 2 weeks. In the YB-0158 preventive study, mice received YB-0158 (5 mg/kg/day; MedChemExpress) or vehicle (2% DMSO, 40% PEG400, 5% Tween-80, 53% saline) by i.p. injection starting 3 days before surgery and continuing for 7 days after surgery.

Angiotensin II (AngII)-Induced Cardiac Hypertrophy

Sam68cKO and CTR mice were implanted with subcutaneous osmotic minipumps (model 2002W, RWD, Shenzhen, China) delivering angiotensin II (Ang II; 1.44 mg/kg/day) or 0.9% saline for 14 days. At the endpoint, physiological indices were assessed and hearts were collected for downstream analyses.

Transverse Aortic Constriction Model

Transverse aortic constriction (TAC) was performed in 10–12-week-old mice (25–27 g) as described previously [49]. Briefly, mice were anesthetized with isoflurane (1.5% in oxygen). After confirming adequate anesthesia, a left thoracotomy was performed to expose the aortic arch. The arch was constricted using a 7-0 silk suture tied around a 26-gauge needle, which was then removed. The thoracic incision was closed with 4-0 sutures, and mice were placed on a warming pad until fully recovered. Sham-operated mice underwent the same procedure without aortic constriction. Successful TAC was confirmed by echocardiography. Cardiac function was assessed 4 weeks after surgery, followed by tissue collection for downstream experiments.

Echocardiography

Transthoracic echocardiography was performed using a VEVO1100 small-animal ultrasound system (FUJIFILM VisualSonics, Canada). Mice were anesthetized with isoflurane (1.5% induction; maintained at ~1.0–1.5% as needed) and placed supine on a temperature-controlled platform maintained at 37°C. M-mode images were acquired to measure interventricular septal thickness (IVS), left ventricular posterior wall thickness (LVPW), and left ventricular internal diameter (LVID) in diastole and systole. Left ventricular ejection fraction (LVEF) and fractional shortening (LVFS) were calculated using Vevo software (LV trace analysis).

Histological Analysis

Hearts were collected, fixed in 4% paraformaldehyde, embedded in paraffin, and sectioned at 4 µm. Hematoxylin and eosin (H&E) and Picrosirius red staining were performed using commercial kits (H&E: G1076; Sirius Red: G1018; Wuhan Servicebio Technology Co., Ltd.). Whole-slide images were acquired using a digital slide scanner (Panoramic MIDI, 3DHISTECH, Hungary). Collagen volume fraction was quantified using Image-Pro Plus (v6.0) as the ratio of Picrosirius red–positive area to total tissue area.

Immunofluorescence Staining

Paraffin sections were deparaffinized and rehydrated through graded alcohols. Antigen retrieval was performed using 1× sodium citrate buffer (Cat. PN4115, G-CLONE) under high-temperature conditions. Sections were washed in PBS and blocked with QuickBlock™ Blocking Buffer for Immunostaining (Beyotime, P0260) for 30 min at room temperature, then incubated overnight at 4°C with primary antibodies: anti-Sam68 (Abcam, ab76471; 1:50) and anti–α-actinin (BOSTER, BM0003; 1:200). After washing, sections were incubated for 1 h at room temperature with secondary antibodies: Alexa Fluor 488 goat anti-mouse (Invitrogen, A11029; 1:400) and Alexa Fluor 555 goat anti-rabbit (Invitrogen, A21428; 1:400). Wheat germ agglutinin (WGA) staining was performed using iF488-WGA (Servicebio, G1730) or iF555-WGA (Servicebio, G1731) as indicated. Nuclei were counterstained with DAPI, and slides were mounted using VectaMount™ AQ (Vector Laboratories, 5501). Images were acquired using a digital slide scanner (Panoramic MIDI) or a fluorescence microscope (Nikon Eclipse Ti2, Japan).

RNA Extraction and Quantitative Real-Time PCR

Gene expression was quantified by quantitative real-time PCR (qRT-PCR). Total RNA was reverse-transcribed to cDNA, and qRT-PCR was performed using ChamQ Universal SYBR qPCR Master Mix (Vazyme, Q711-03) on a Roche LightCycler 480 II Real-Time PCR system. Transcript levels of genes of interest were normalized to 18S rRNA. Primer sequences are listed in **Table S10**.

Western Blotting

Tissues and cells were lysed in RIPA buffer (Epizyme, PC101/PC102) supplemented with protease inhibitor cocktail (MCE, HY-K0010) and phosphatase inhibitor cocktails I and II (MCE, HY-K0021 and HY-K0022). Protein samples were denatured at 95°C for 10 min, separated by SDS-PAGE, and transferred onto PVDF membranes (Merck). Membranes were blocked in 5% non-fat milk for 30 min, incubated with primary antibodies overnight at 4°C, and then incubated with appropriate secondary antibodies. Signals were developed using enhanced chemiluminescence (ECL; YEASEN) and imaged on a Tanon 6100C system. Antibody details are provided in **Table S11**.

Isolation and Treatment of Neonatal Rat Ventricular Myocytes

Neonatal rat ventricular myocytes (NRVMs) were isolated from 1–3-day-old Sprague–Dawley rats. Ventricles were dissected, minced (~1 mm^3), and incubated in 0.05% trypsin (without EDTA) overnight at 4°C. Tissue fragments were then digested in 0.2% type II collagenase at 37°C for 45 min with gentle agitation. Cell suspensions were filtered through a 75-μm strainer (2–3 passes) until digestion was complete, then centrifuged at 800 × g for 5 min. Cells were resuspended in DMEM (C11995500BT) with 15% FBS and pre-plated for 1 h in 100-mm dishes to reduce fibroblast contamination. Non-adherent cardiomyocyte-enriched supernatants were transferred to collagen-coated dishes. After 24 h, cells were washed and medium was replaced before treatments. Where indicated, NRVMs were treated with phenylephrine (20 μM; Sigma, P6126), Ang II (1 μM; Sigma, A9525), C188-9 (10 μM; MedChemExpress, HY-112288), PP2 (1 μM; MedChemExpress, HY-13805), or YB-0158 (1 μM; MedChemExpress, HY-136541).

RNA Sequencing

RNA-seq was performed by HaploX Medical Laboratory Co., Ltd. Total RNA was extracted from apical regions of Sam68cKO and CTR hearts using TRIzol™ (Invitrogen). RNA quantity and purity were assessed using NanoDrop™ One/OneC and a Qubit RNA BR Assay Kit, and RNA integrity was evaluated using an Agilent 4200 TapeStation. Poly(A)+ RNA was captured using the ABclonal Poly(A) mRNA Capture Module, and libraries were prepared using the ABclonal Fast RNA-seq Library Prep Kit V2 according to the manufacturers' instructions. Library concentration and fragment-size distribution were assessed by Qubit dsDNA HS and Agilent 4200 TapeStation, and library molarity was determined using the KAPA Library Quant kit. Sequencing was performed on an Illumina NovaSeq X Plus platform.

Differential Expression Analysis

A gene-level count matrix was generated from RNA-seq data and normalized to account for sequencing depth and compositional differences between libraries. Differential expressions between groups were tested using a count-based statistical model on normalized counts. P values were adjusted for multiple testing using the Benjamini–Hochberg false discovery rate (FDR) procedure; FDR-adjusted P values are reported as q-values, and genes with $q < 0.05$ were considered differentially expressed.

Pathway Enrichment Analysis

KEGG and Gene Ontology (GO) enrichment analyses were performed using a hypergeometric over-representation test. Enrichment P values were adjusted for multiple comparisons using the Benjamini–Hochberg FDR method; FDR-adjusted enrichment P values are reported as q-values, and terms with $q \leqslant 0.05$ were considered significantly enriched.

Targeted Metabolomics

Targeted energy-metabolome profiling was performed by Biotree Biotech Co., Ltd. (Shanghai, China), and data were processed using the MetWare cloud platform. Heart tissues were thawed on ice and homogenized. For each sample, 25 mg tissue was extracted in 500 μL of 70% methanol/water, vortexed, and centrifuged at 12,000 rpm for 10 min at 4°C. Supernatants were incubated at −20°C for 30 min and centrifuged again under the same conditions. An aliquot of the clarified supernatant was passed through a protein precipitation plate and analyzed by LC-ESI-MS/MS. Significantly regulated metabolites were identified using variable importance in projection (VIP) values and absolute fold changes. VIP values were derived from OPLS-DA using MetaboAnalystR in R after mean-centering. A 200-permutation test was used to assess overfitting.

[U-^{13}C]-Glucose Flux Assays

In vivo [U-$^{13}C_6$]-glucose tracing was performed in CTR and Sam68cKO mice subjected to sham or TAC surgery using established approaches [50-52]. At 3 days or 4 weeks after surgery, mice were fasted overnight (12–16 h) and injected intravenously with [U-$^{13}C_6$]-glucose (Cambridge Isotope Laboratories, CLM-1396-1; 1 mg/g body weight). After 20 min, hearts were harvested and flash-frozen in liquid nitrogen. Frozen samples were processed and analyzed by Biotree Biotech Co., Ltd. for ^{13}C enrichment and isotopologue distributions by mass spectrometry.

For metabolite extraction, frozen ventricular tissue (30–40 mg) was homogenized on dry ice in pre-chilled (−40°C) 50% (v/v) methanol/water and subjected to one freeze–thaw cycle (30 min on dry ice, then 5 min on ice). Chloroform (400 μL) was added, samples were vortexed, and then centrifuged (12,000 × g, 15 min, 4°C). Supernatants were stored

at −80°C prior to analysis.

Polar metabolites were measured on a Q Exactive PLUS hybrid quadrupole–Orbitrap mass spectrometer (Thermo Fisher Scientific) coupled to HILIC. Separation was performed on an XBridge BEH Amide column (150 × 2.1 mm, 2.5 μm; Waters) using mobile phase A (95:5 H_2O:acetonitrile containing 20 mM ammonium acetate and 20 mM ammonium hydroxide, pH 9.4) and mobile phase B (100% acetonitrile). Flow rate was 150 μL/min, injection volume was 5 μL, and column temperature was 25°C. Data were acquired in negative ion mode at 140,000 resolution (m/z 200), with an AGC target of 1 × 10^6, scan range m/z 75–1000. Isotope labeling data were analyzed in El-MAVEN with natural-abundance correction.

Pyruvate Dehydrogenase Activity Assay

Pyruvate dehydrogenase (PDH) activity in cardiac tissue was measured using a commercial kit (BC0385; Solarbio, China). Approximately 0.1 g tissue was homogenized in the supplied buffer on ice and centrifuged at 11,000 × g for 10 min at 4°C. Ten microliters of supernatant was added to a 96-well plate, followed by 180 μL reaction buffer. Absorbance at 605 nm was recorded at 10 s and 70 s. PDH activity was calculated from the linear decrease in absorbance reflecting reduction of 2,6-dichlorophenolindophenol (2,6-DCPIP).

Protein–Protein Docking

Protein–protein docking was performed using HDOCK (hybrid FFT-based global search with knowledge-based scoring refinement). Structures were obtained from the Protein Data Bank (PDB): Sam68 KH domain (PDB: 2XA6), STAT3 (PDB: 6NJS), and Src (PDB: 2SRC). A full-length Sam68 model was obtained from AlphaFold (UniProt: Q07666; AF-Q07666-F1). Structures were prepared using standard preprocessing in the HDOCK pipeline (including removal of crystallographic water), and relevant protein pairs were docked to generate candidate complexes. Models were ranked by HDOCK score, and top-ranked complexes were examined for interface complementarity and putative binding residues.

Co-immunoprecipitation

Co-immunoprecipitation (co-IP) was performed using the Pierce™ Classic Magnetic IP/Co-IP Kit (Thermo Fisher, 88804). Cardiac tissues or NRVMs were lysed in Pierce lysis buffer (pH 7.4; 25 mM Tris, 150 mM NaCl, 1 mM EDTA, 1% NP-40, 5% glycerol) supplemented with protease and phosphatase inhibitors (MCE). After centrifugation, protein concentration was determined. For each IP, 200 μg lysate was incubated overnight

at 4°C with 2 μg antibody (anti-Flag, Sigma F1804; anti-Sam68, Santa Cruz sc-1238; anti-STAT3, Proteintech 10253-2-AP) or anti-Flag magnetic beads (MCE, HY-K0207). Beads were washed twice, and immune complexes were eluted and neutralized per kit instructions before SDS-PAGE and immunoblotting (or mass spectrometry, where indicated).

Subcellular Fractionation

Nuclear and Cytosolic Fractions. Nuclear and cytosolic proteins were prepared from NRVMs as described previously [53]. Briefly, 1–2 × 10^6 cells were washed in cold PBS and resuspended in 65 μL ice-cold 0.1% NP-40 PBS with protease/phosphatase inhibitors. Lysates were briefly centrifuged (10 s), and 60 μL supernatant was collected as the cytosolic fraction and mixed with 3× SDS buffer, then boiled for 5 min. Pellets were washed twice with 0.1% NP-40 PBS, resuspended, mixed with 3× SDS buffer, boiled for 5 min, and sonicated to shear DNA (nuclear fraction).

Mitochondrial Isolation. Following adenoviral transduction and AngII treatments, NRVM mitochondria were isolated using a Cell Mitochondria Isolation Kit (Beyotime) according to the manufacturer's protocol. Cells were homogenized in extraction buffer, incubated on ice for 15 min, and centrifuged at 600 × g for 10 min to remove nuclei/debris. Supernatants were centrifuged at 11,000 × g for 10 min to pellet mitochondria. The post-mitochondrial supernatant was retained as the cytosolic fraction; mitochondrial pellets were resuspended for downstream analyses.

Lentiviral and Adenoviral Transduction

Sam68 shRNA oligonucleotides were obtained from Sigma (TRCN0000102324). Lentivirus was produced in HEK293T cells by co-transfecting pLenti-XI-Puro-shKHDRBS1 with packaging plasmids. NRVMs were seeded at 1 × 10^6 cells/well and transduced with 5 × 10^6 viral particles in the presence of 8 μg/mL polybrene for 24 h. Cells were then cultured in serum-free medium and treated with Ang II (1 μM) for an additional 24 h as indicated.

Recombinant adenoviruses were generated using the pADM system. The Sam68-3×Flag cassette was cloned into pADM-CMV-C-3×Flag-mCMV-copGFP for overexpression, and Sam68 shRNA was cloned into pADM-U6-shRNA-mCMV-copGFP for knockdown. Viruses were packaged in HEK293T cells using polyethylenimine (PEI). NRVMs were infected in serum-free medium at MOI 100 for 24 h.

Pharmacokinetics of YB-0158

YB-0158 pharmacokinetics were assessed following a single i.p. injection at 5 mg/kg or 10 mg/kg (10 mL/kg dosing volume). Blood (≥0.3 mL; terminal collection via abdominal aorta under isoflurane anesthesia) was collected at 0.167, 0.5, 2, and 6 h post-dose (n = 6 per time point). Blood was mixed with heparin, centrifuged (2,000 × g, 10 min, 4°C) within 2 h, and plasma stored at −30°C. Hearts were harvested, rinsed, and stored at −30°C.

Plasma and heart homogenates (25 μL) were protein-precipitated with 75 μL internal standard precipitant (acetonitrile containing 50 ng/mL tolbutamide), vortexed, centrifuged, and analyzed by LC-MS/MS using a Waters ACQUITY UPLC I-Class Xevo TQ-S system with a BEH C18 column (2.1 × 50 mm, 1.7 μm). Calibration standards (10–1,000 ng/mL) and QC samples were prepared in blank mouse plasma to ensure assay accuracy and precision.

Dual-Luciferase Reporter Assay

HEK293T cells were seeded in 24-well plates (5 × 10^4 cells/well) and co-transfected 24 h later with 250 ng Sam68 promoter firefly luciferase reporter (~1.5 kb mouse Sam68 promoter), 160 ng pRL-TK Renilla luciferase control plasmid, and increasing amounts (0–300 ng) of full-length STAT3 expression vector using jetPRIME (Polyplus) at a 2:1 reagent:DNA ratio. Thirty-six hours after transfection, cells were lysed and luciferase activities were measured using a Dual-Luciferase Reporter Assay system (Yeasen). Firefly luciferase activity was normalized to Renilla luciferase activity and expressed as fold change relative to empty-vector control.

Supplementary Tables

Table S1: Echocardiographic parameters of Sam68cKO and control (CTR) mice at 2 weeks after sham or TAC

	Sham		TAC	
	CTR	Sam68-cKO	CTR	Sam68-cKO
n	4	4	8	8
Heart rate	459.0±17.15	465.4±12.22	453.8±15.47	465.6±11.97
IVS; d (mm)	0.768±0.056	0.786±0.023	1.455±0.070****	0.959±0.068$^{\#\#\#\#}$
IVS; s (mm)	1.287±0.030	1.202±0.065	1.881±0.071***	1.4980±0.076$^{\#\#}$
LVID; d (mm)	3.866±0.158	3.318±0.359	3.940±0.200	3.910±0.179
LVID; s (mm)	2.595±0.093	2.272±0.285	2.731±0.238	2.703±0.184
LVPW; d (mm)	0.553±0.032	0.631±0.049	1.025±0.064****	0.859±0.034
LVPW; s (mm)	0.813±0.071	0.814±0.058	1.285±0.056***	1.084±0.066
EF (%)	62.71±0.572	61.90±2.084	59.38±4.239	56.72±2.565
FS (%)	32.81±0.983	31.82±1.667	31.43±2.640	30.78±1.674

Abbreviations: EF, ejection fraction; FS, fractional shortening; IVS;d, interventricular septal thickness at end-diastole; IVS;s, interventricular septal thickness at end-systole; LVPW;d, left ventricular posterior wall thickness at end-diastole; LVPW;s, left ventricular posterior wall thickness at end-systole; LVID;d, left ventricular internal diameter at end-diastole; LVID;s, left ventricular internal diameter at end-systole.

Statistics: Mean ± SEM. Two-way ANOVA with Tukey's multiple-comparisons test. ***P < 0.001, ****P < 0.0001 vs. Sham+CTR; $^{\#\#}$P < 0.01, $^{\#\#\#\#}$P < 0.0001 vs. TAC+CTR.

Table S2: Echocardiographic parameters of Sam68cKO and CTR mice at 4 weeks after sham or TAC

	Sham		TAC	
	CTR	Sam68-cKO	CTR	Sam68-cKO
n	7	7	13	12
Heart rate	465.3±8.279	458.7±10.363	464.3±10.775	472.9±5.135
IVS; d (mm)	0.816±0.065	0.746±0.08	1.080±0.051*	1.281±0.058
IVS; s (mm)	1.330±0.078	1.335±0.111	1.422±0.080	1.787±0.054##
LVID; d (mm)	3.542±0.101	3.508±0.114	4.392±0.178**	3.617±0.167#
LVID; s (mm)	2.356±0.1	2.277±0.151	3.549±0.211***	2.668±0.128##
LVPW; d (mm)	0.630±0.039	0.622±0.039	0.832±0.050*	0.868±0.053
LVPW; s (mm)	0.844±0.046	0.885±0.045	1.015±0.058	1.154±0.045
EF (%)	65.08±2.758	66.00±3.216	40.86±3.258****	52.29±1.622#
FS (%)	33.60±1.508	35.36±2.901	19.96±1.904****	26.25±0.989#

Abbreviations: As in **Table S1**.
Statistics: Mean ± SEM. Two-way ANOVA with Tukey's multiple-comparisons test. *P < 0.05, **P < 0.01, ***P < 0.001, ****P < 0.0001 vs. Sham+CTR; #P < 0.05, ##P < 0.01 vs. TAC+CTR.

Table S3: Echocardiographic parameters of Sam68cKO and CTR mice after 2 weeks of Ang II infusion

	Saline		AngII	
	CTR	Sam68-cKO	CTR	Sam68-cKO
n	7	7	8	7
Heart rate	470.6±11.55	470.3±8.559	459.8±5.874	475.6±7.691
IVS; d (mm)	0.664±0.058	0.765±0.072	1.376±0.067***	1.019±0.028$^{\#\#\#}$
IVS; s (mm)	1.173±0.043	1.214±0.05	2.058±0.077****	1.489±0.06$^{\#\#\#\#}$
LVID; d (mm)	3.694±0.112	3.529±0.151	3.583±0.187	3.483±0.066
LVID; s (mm)	2.487±0.104	2.321±0.121	2.271±0.179	2.258±0.066
LVPW; d (mm)	0.704±0.034	0.634±0.035	1.019±0.036****	0.748±0.048$^{\#\#\#}$
LVPW; s (mm)	0.842±0.045	0.781±0.031	1.291±0.033****	0.992±0.042$^{\#\#\#\#}$
EF (%)	63.18±1.139	62.51±1.239	66.83±2.122	63.19±1.039
FS (%)	33.63±0.99	33.00±0.84	36.45±1.549	33.43±0.764

Abbreviations: As in **Table S1**; Ang II, angiotensin II.
Statistics: Mean ± SEM. Two-way ANOVA with Tukey's multiple-comparisons test. ***P < 0.001, ****P < 0.0001 vs. Saline+CTR; $^{\#\#\#}$P < 0.001, $^{\#\#\#\#}$P < 0.0001 vs. Ang II+CTR.

Table S4: Echocardiographic parameters of Sam68OE and GFP control mice at 2 weeks after sham or TAC

	Sham		TAC	
	GFP	Sam68OE	GFP	Sam68OE
n	7	10	7	12
Heart rate	463.8±13.281	474.4±8.236	447.6±6.115	481.4±10.046
IVS; d (mm)	1.129±0.037	1.201±0.036	1.502±0.036****	1.665±0.034$^{\#}$
IVS; s (mm)	1.547±0.060	1.662±0.068	1.913±0.033**	2.102±0.057
LVID; d (mm)	3.589±0.066	3.571±0.093	3.646±0.098	3.546±0.122
LVID; s (mm)	2.362±0.038	2.444±0.063	2.452±0.094	2.371±0.140
LVPW; d (mm)	0.804±0.034	0.717±0.036	0.929±0.036	1.077±0.048
LVPW; s (mm)	0.991±0.043	0.889±0.044	1.228±0.041*	1.340±0.051
EF (%)	63.45±1.097	60.92±1.486	62.20±1.711	61.76±3.774
FS (%)	34.15±0.631	31.54±0.632	33.65±1.159	33.39±2.540

Abbreviations: As in **Table S1**.
Statistics: Mean ± SEM. Two-way ANOVA with Tukey's multiple-comparisons test. *P < 0.05, **P < 0.01, ****P < 0.0001 vs. Sham+GFP; $^{\#}$P < 0.05 vs. TAC+GFP.

Table S5: Echocardiographic parameters of Sam68OE and GFP control mice at 4 weeks after sham or TAC

	Sham		TAC	
	GFP	Sam68OE	GFP	Sam68OE
n	7	10	9	14
Heart rate	472.4±6.853	483.7±12.081	482.4±8.396	475.2±8.401
IVS; d (mm)	0.977±0.039	1.052±0.056	1.389±0.051**	1.323±0.081
IVS; s (mm)	1.575±0.039	1.511±0.064	1.772±0.039	1.723±0.072
LVID; d (mm)	3.622±0.108	3.270±0.101	4.258±0.110***	4.639±0.067##
LVID; s (mm)	2.351±0.086	2.204±0.079	3.059±0.068***	3.534±0.055#
LVPW; d (mm)	0.875±0.076	0.774±0.038	0.898±0.032	0.932±0.065
LVPW; s (mm)	1.095±0.077	0.864±0.052	1.190±0.047	1.155±0.067
EF (%)	65.16±1.219	62.49±1.001	54.62±1.083****	47.35±1.193###
FS (%)	35.12±1.055	32.66±0.651	28.10±0.554****	23.81±0.652####

Abbreviations: As in **Table S1**.

Statistics: Mean ± SEM. Two-way ANOVA with Tukey's multiple-comparisons test. **P < 0.01, ***P < 0.001, ****P < 0.0001 vs. Sham+GFP; #P < 0.05, ##P < 0.01, ###P < 0.001, ####P < 0.0001 vs. TAC+GFP.

Table S6: Echocardiographic parameters of GFP and Sam68OE mice treated with vehicle or PDK-IN at 4 weeks after TAC

	Sham+ Vehicle		TAC+Vehicle		TAC+PDK4-IN	
	GFP	Sam68OE	GFP	Sam68OE	GFP	Sam68OE
n	7	7	9	6	10	6
Heart rate	474.4±5.902	488.0±5.973	477.1±6.674	487.9±8.688	488.0±11.887	488.2±8.870
IVS; d (mm)	1.125±0.050	1.060±0.075	1.099±0.066	1.217±0.048	1.220±0.080	1.389±0.029
IVS; s (mm)	1.786±0.068	1.608±0.068	1.463±0.075*	1.598±0.042	1.760±0.079$^{\#}$	1.948±0.042$^{\#\#\#\#+}$
LVID; d (mm)	3.404±0.134	3.721±0.130	3.842±0.116	4.525±0.095$^{****\#}$	3.643±0.161^{+++}	4.203±0.113**
LVID; s (mm)	2.012±0.122	2.399±0.109	2.764±0.096**	3.606±0.088$^{****\#\#\#}$	2.320±0.173^{++++}	2.865±0.061^{**++}
LVPW; d (mm)	0.802±0.043	0.784±0.043	0.771±0.060	0.815±0.048	0.913±0.032	0.938±0.022
LVPW; s (mm)	0.993±0.040	1.002±0.081	1.000±0.057	1.098±0.082	1.223±0.036	1.305±0.065$^{*\#}$
EF (%)	72.85±2.940	67.69±2.028	55.85±1.372***	42.27±0.672$^{****\#\#}$	67.10±3.170$^{\#\#++++}$	60.81±1.596^{*+++}
FS (%)	41.35±2.591	36.30±1.701	26.92±1.319	20.33±0.431	36.99±2.302	32.10±1.061

Abbreviations: As in **Table S1**.
Statistics: Mean ± SEM. Two-way ANOVA with Tukey's multiple-comparisons test.
$^{*}P < 0.05$, $^{**}P < 0.01$, $^{***}P < 0.001$, $^{****}P < 0.0001$ vs. Sham+Vehicle+GFP;
$^{\#}P < 0.05$, $^{\#\#}P < 0.01$, $^{\#\#\#}P < 0.001$, $^{\#\#\#\#}P < 0.0001$ vs. TAC+Vehicle+GFP;
$^{+}P < 0.05$, $^{++}P < 0.01$, $^{+++}P < 0.001$, $^{++++}P < 0.0001$ vs. TAC+Vehicle+Sam68OE.

Table S7: Echocardiographic parameters of vehicle- and YB-0158–treated mice at 4 weeks after sham or TAC

	Sham		TAC	
	Vehicle	YB-0158	Vehicle	YB-0158
n	5	7	12	11
Heart rate	497.5±15.120	500.8±13.560	466.3±9.676	467.0±9.581
IVS; d (mm)	1.229±0.051	1.036±0.063	1.104±0.067	1.248±0.058
IVS; s (mm)	1.745±0.083	1.571±0.076	1.501±0.059	1.725±0.045
LVID; d (mm)	3.193±0.139	3.611±0.166	4.036±0.088***	3.702±0.078
LVID; s (mm)	1.972±0.133	2.292±0.151	2.993±0.076****	2.465±0.096#
LVPW; d (mm)	0.965±0.043	0.918±0.023	0.810±0.043	0.936±0.049
LVPW; s (mm)	1.190±0.047	1.179±0.058	0.988±0.053	1.164±0.052
EF (%)	72.11±0.948	68.61±1.557	53.57±1.990****	61.96±2.973#
FS (%)	40.12±0.703	37.71±1.247	25.87±0.894****	33.15±1.948#

Abbreviations: As in **Table S1**.
Statistics: Mean ± SEM. Two-way ANOVA with Tukey's multiple-comparisons test. ***P < 0.001, ****P < 0.0001 vs. Sham+Vehicle; #P < 0.05 vs. TAC+Vehicle.

Table S8: Echocardiographic parameters of vehicle- and YB-0158–treated CTR and Sam68cKO mice at 4 weeks after TAC

	Sham+ Vehicle		TAC+Vehicle		TAC+YB-0158	
	CTR	Sam68cKO	CTR	Sam68cKO	CTR	Sam68cKO
n	7	7	9	7	9	7
Heart rate	459.3±10.234	465.1±7.428	480.4±10.276	466.1±10.314	482.2±11.925	468.7±7.047
IVS; d (mm)	0.867±0.044	1.085±0.044	1.123±0.037	1.082±0.052	1.240±0.104**	0.969±0.039
IVS; s (mm)	1.449±0.048	1.694±0.069	1.526±0.036	1.582±0.042	1.510±0.087	1.400±0.047
LVID; d (mm)	2.874±0.087	3.010±0.045	3.685±0.057****	3.172±0.068$^{\#\#\#\#}$	3.130±0.085$^{\#\#\#\#}$	3.222±0.052$^{*\#\#\#}$
LVID; s (mm)	1.648±0.085	1.782±0.048	2.729±0.050****	1.955±0.076$^{*\#\#\#\#}$	2.021±0.084$^{**\#\#\#\#}$	2.155±0.053$^{***\#\#\#\#}$
LVPW; d (mm)	0.765±0.019	0.774±0.078	0.964±0.061	0.789±0.048	0.955±0.040	0.800±0.042
LVPW; s (mm)	1.085±0.047	1.103±0.091	1.118±0.069	1.122±0.064	1.292±0.089	1.125±0.054
EF (%)	75.33±2.400	72.98±1.612	50.67±0.958****	68.32±1.586$^{\#\#\#\#}$	66.02±1.855$^{**\#\#\#\#}$	62.18±1.962$^{***\#\#\#}$
FS (%)	42.66±2.411	40.67±1.334	25.11±0.555****	37.24±1.205$^{\#\#\#\#}$	35.52±1.374$^{*\#\#\#\#}$	33.11±1.419$^{***\#\#}$

Abbreviations: As in **Table S1**.

Statistics: Mean ± SEM. Two-way ANOVA with Tukey's multiple-comparisons test. *P < 0.05, **P < 0.01, ***P < 0.001, ****P < 0.0001 vs. Sham+CTR+Vehicle; $^{\#\#}$P < 0.01, $^{\#\#\#}$P < 0.001, $^{\#\#\#\#}$P < 0.0001 vs. TAC+CTR+Vehicle.

Table S9: Clinical characteristics of HF patients and non-failing controls

Type	No.	Sex	Age (y)	LVEF (%)	BCD	SAM	AF	DM	HTN	GDMT			
										ARNI/ ACEI/ ARB	β-block	MRA	SGLT2i
NF	1	M	32	62	N/A	N	N	N	N	N	N	N	N
	2	F	38	65	N/A	N	N	N	N	N	N	N	N
	3	M	35	62	N/A	N	N	N	N	N	N	N	N
	4	M	28	68	N/A	N	N	N	N	N	N	N	N
	5	F	31	63	N/A	N	N	N	N	N	N	N	N
HF	1	M	62	22.7	RCM	N	Y	Y	N	Y	Y	Y	Y
	2	M	28	24	RCM	N	Y	N	N	Y	Y	Y	Y
	3	M	46	17	RCM	N	Y	Y	Y	Y	Y	Y	Y
	4	M	35	16.2	RCM	N	Y	N	Y	Y	Y	Y	Y
	5	F	61	25.8	RCM	N	Y	N	N	Y	N	Y	Y
	6	M	35	25.1	RCM	N	N	N	N	N	Y	N	N
	7	F	54	22.7	RCM	N	Y	N	N	Y	N	Y	Y
	8	M	43	20	RCM	N	N	N	N	N	Y	N	N
	9	M	56	53.9	HCM	N	Y	Y	Y	Y	Y	Y	N
	10	M	56	31	HCM	N	N	N	N	N	N	Y	N
	11	M	66	55	HCM	Y	N	N	N	N	N	N	Y

Effect sizes

Variable	NF (n=5)	HF (n=11)	Effect Size (95% CI)	p-value
**Age (y)**	32.8±4.0	50.2±13.4	Hedges' g: 1.41 (0.25, 2.57)	0.02
**Male, n (%)**	3 (60%)	9 (81.8%)	RD: 0.22 (-0.27, 0.71)	0.67
**LVEF (%)**	64.0±2.5	28.5±13.3	Hedges' g: -3.10 (-4.59, -1.61)	<0.001

LV tissue from HF patients was obtained at transplantation (restrictive or hypertrophic cardiomyopathy). Non-failing LV tissue was obtained from organ donors whose hearts were declined for transplantation, with research consent provided by next of kin.

Data presentation and statistics: Individual-level clinical variables are shown. Group summaries are presented as mean ± SD or n (%). Effect sizes are reported as Hedges' g (continuous variables) or risk difference (RD; binary variables) with 95% confidence intervals (CIs). Continuous variables were compared using the Mann–Whitney U test, and categorical variables using Fisher's exact test.

Abbreviations: BCD, baseline cardiac disease; RCM, restrictive cardiomyopathy; HCM, hypertrophic cardiomyopathy; SAM, systolic anterior motion (mitral valve); AF, atrial fibrillation;

DM, diabetes mellitus; HTN, hypertension; GDMT, guideline-directed medical therapy; ARNI, angiotensin receptor–neprilysin inhibitor; ACEI, angiotensin-converting enzyme inhibitor; ARB, angiotensin receptor blocker; β-blocker, β-adrenergic receptor blocker; MRA, mineralocorticoid receptor antagonist; SGLT2i, sodium–glucose cotransporter-2 inhibitor; Y, yes; N, no.

Table S10. Primer sequences used for qRT-PCR

Oligo name	Oligo sequence
m*Nppa*_R	CCTTGGCTGTTATCTTCGGTACCGG
m*Nppa*_F	ACCTGCTAGACCACCTGGAG
m*Nppb*_R	GCCATTTCCTCCGACTTTTCTC
*mNppb*_F	GAGGTCACTCCTATCCTCTGG
m18S_R	AGCTTATGACCCGCACTTAC
m18S_F	GTCTGTGATGCCCTTAGATG
m*Sam68*_R	CTCCTCGTCCTCTCACAGATA
m*Sam68*_F	GATATCTGTCAGGAGCAGTTTCT

Table S11. Antibodies and reagents

REAGENT or RESOURSE	SOURCE	IDENTIFIER	Dilution
**Antibodies**			
WB/IF: Recombinant anti-Sam68 antibody [EPR3231]	Abcam	Ab76471	1:1000 (WB)<br>1:50 (IF)
IP: Sam68 antibody (7-1)	Santa Cruz Biotechnology	Sc-1238	1:50 (IP)
WB: ANP antibody (F-2)	Santa Cruz Biotechnology	Sc-515701	1:1000 (WB)
WB: BNP Rabbit mAb	Abclonal	A23996	1:1000 (WB)
IF: Anti-Alpha Actinin/ACTN2 Antibody (Clone#EA-53)	BOSTER	BM0003	1:100 (IF)
WB: Total OXPHOS Rodent Antibody Cocktail	Abcam	Ab110413	1:1000 (WB)
WB: PDK4 Polyclonal antibody	Proteintech	12949-1-AP	1:1000 (WB)
WB: Pyruvate Dehydrogenase Antibody	Cell Signaling Technology	2784S	1:1000 (WB)
WB: Phospho-Pyruvate Dehydrogenase α1 (S293) Antibody	Cell Signaling Technology	31866S	1:1000 (WB)
WB/IP: STAT3 Polyclonal Antibody	Proteintech	10253-2-AP	1:1000 (WB)
WB: STAT3 (124H6) Mouse mAb	Cell Signaling Technology	9139T	1:1000 (WB)
WB: Phospho-STAT3 (Tyr 705) Rabbit pAb	ZENBio	381552	1:1000 (WB)
WB: Phospho-STAT3 (Ser 727) Rabbit pAb	Cell Signaling Technology	9134	1:1000 (WB)
WB: Monoclonal Anti-Flag M2 antibody	Sigma-Aldrich	F1804	1:1000 (WB)<br>1:50 (IP)
WB: Anti-beta Actin Rabbit mAb	PTM Bio	PTM-5028	1:1000 (WB)
WB: Anti-GFP Rabbit mAb	PTM Bio	PTM-6673	1:1000 (WB)
WB: HSP90 Monoclonal antibody	proteintech	60318-1-Ig	1:1000 (WB)
WB: VDAC1/Porin Polyclonal antibody	proteintech	55259-1-AP	1:1000 (WB)
WB: HRP, Goat Anti-Rabbit IgG	Abbkine	A21020	1:5000 (WB)
WB: HRP, Goat Anti Mouse IgG	Abbkine	A21010	1:5000 (WB)
IP: IPKINE™ HRP Goat Anti-Mouse IgG HCS	Abbkine	A25112	1:5000 (WB)
IP: IPKINE™ HRP Goat Anti-Rabbit IgG HCS	Abbkine	A25222	1:5000 (WB)
WB: Phospho-Src Family Src (Tyr416) (D49G4) Rabbit mAb	Cell Signaling Technology	6943S	1:1000 (WB)
WB: Src Antibody	MedChemExpress	HY-P80338	1:1000 (WB)
WB: c-Src Polyclonal antibody	proteintech	11097-1-AP	1:1000 (WB)
WB: Lamin B1 Monoclonal antibody	proteintech	HRP-66095	1:1000 (WB)

WB: GAPDH Monoclonal antibody	proteintech	60004-1-Ig	1:1000 (WB)
**Reagents**			
YB-0158	MedChemExpress	HY-136541	5mg/kg/day;10mg/kg (*in vivo*)
			1μM (*in vitro*)
PDK4-IN-1 hydrochloride	MedChemExpress	HY-135954A	5mg/kg/day (*in vivo*)
C188-9	MedChemExpress	HY-112288	10μM (*in vitro*)
PP2	MedChemExpress	HY-13805	1μM (*in vitro*)
Tamoxifen	Sigma-Aldrich	T5648	20mg/kg/day (*in vivo*)
Phosphatase Inhibitor Coctail II	MedChemExpress	HY-K0022	1:100
Phosphatase Inhibitor Coctail I	MedChemExpress	HY-K0021	1:100
Protease Inhibitor Cocktail	MedChemExpress	HY-K0010	1:100

Abbreviations: WB, western blot; IF, immunofluorescence; IP, immunoprecipitation.

Supplementary Figures

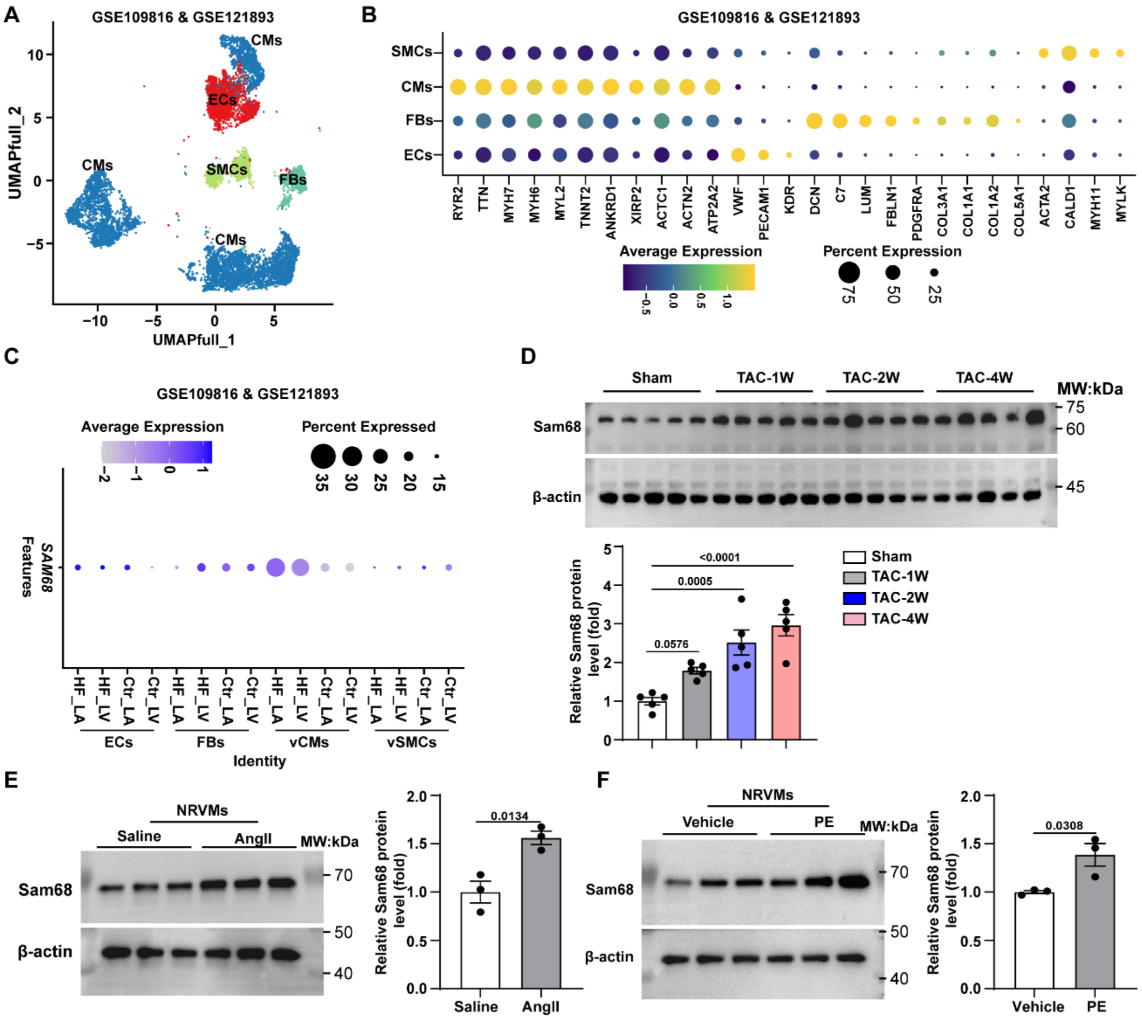

Figure S1. Single-cell RNA-seq identifies cardiomyocyte-enriched Sam68 upregulation in human heart failure. A, UMAP of left atrial (LA) and left ventricular (LV) cells from healthy donors (GSE109816, n=12) and heart failure (HF) patients (GSE121893, n=8). **B,** Dot plot of canonical marker genes defining major cardiac clusters, including ventricular cardiomyocytes (CMs), smooth muscle cells (SMCs), fibroblasts (FBs), and endothelial cells (ECs). **C,** Sam68 (KHDRBS1) expression across major cardiac cell types in control vs. HF samples from LA and LV. **D,** Sam68 protein abundance in mouse hearts at 1, 2, and 4 weeks after sham or TAC (immunoblot and quantification). **E and F,** Sam68 protein levels in neonatal rat ventricular myocytes (NRVMs) treated for 24h with saline vs Ang II (1 μM) (**E**) or vehicle vs phenylephrine (PE, 20 μM) (**F**) (immunoblots and quantification). Data are mean ± SEM. Statistics: one-way ANOVA with Dunnett's multiple-comparisons test (D) or unpaired two-tailed Student's t-test (E–F).

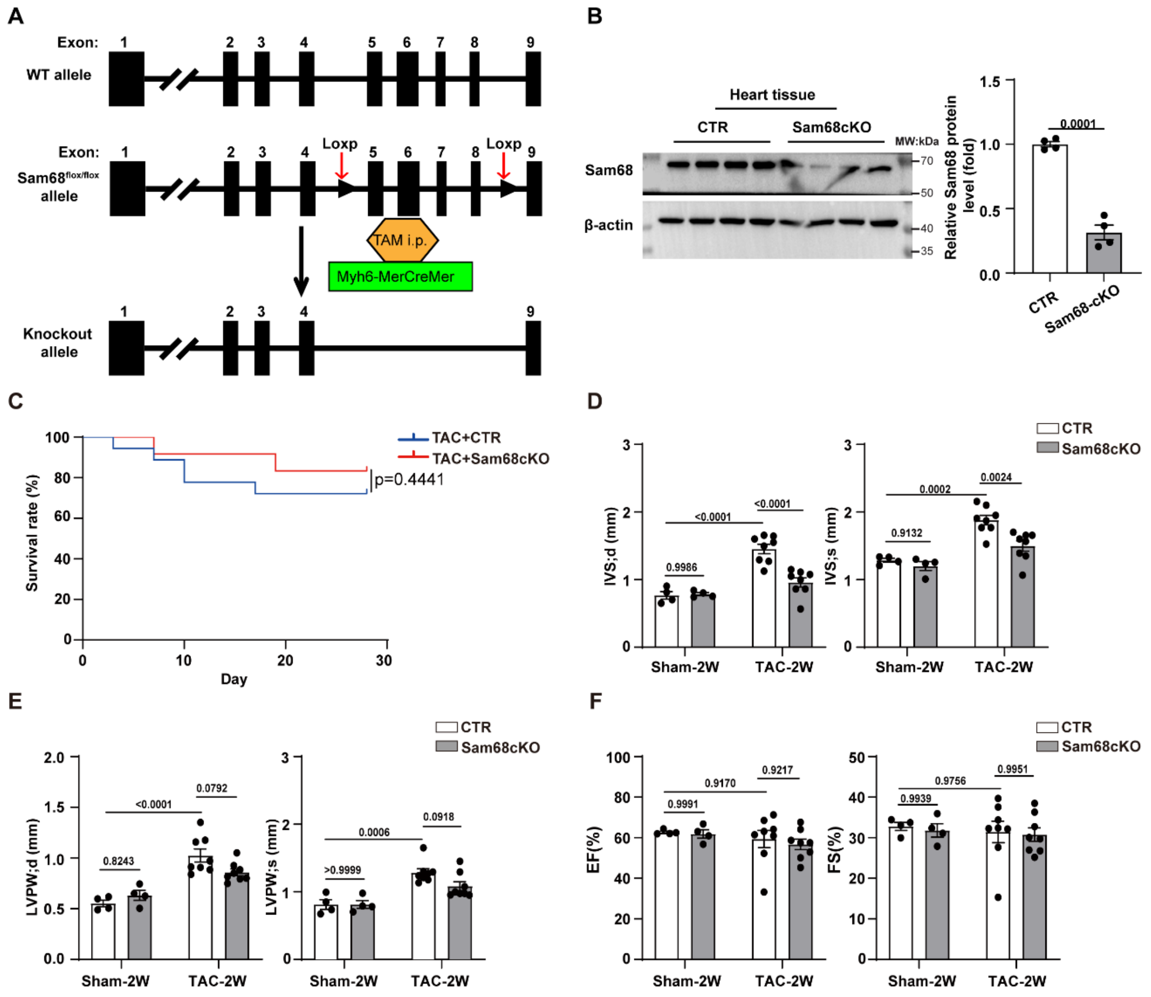

Figure S2. Generation and validation of inducible cardiomyocyte-specific Sam68 knockout mice. A, Targeting and breeding strategy to generate tamoxifen-inducible cardiomyocyte Sam68 knockout mice (Sam68$^{flox/flox}$;α-MHC–MerCreMer; Sam68cKO) and Cre-only littermate controls (α-MHC–MerCreMer; CTR). **B,** Validation of Sam68 deletion in heart tissue 2 weeks after tamoxifen administration (i.p.) (immunoblot). **C,** Kaplan–Meier survival analysis following TAC (CTR, n=18; Sam68cKO, n=12). **D through F,** Echocardiography at 2 weeks after sham or TAC: IVS thickness at end-diastole and end-systole (IVS;d and IVS;s) (**D**); LVPW thickness at end-diastole and end-systole (LVPW;d and LVPW;s) (**E**); ejection fraction (EF) and fractional shortening (FS) (**F**) (sham, n=4/group; TAC, n=8/group). Data are mean ± SEM. Statistics: unpaired two-tailed Student's t-test (B), log-rank test (C), and two-way ANOVA with Tukey's multiple-comparisons test (D through F).

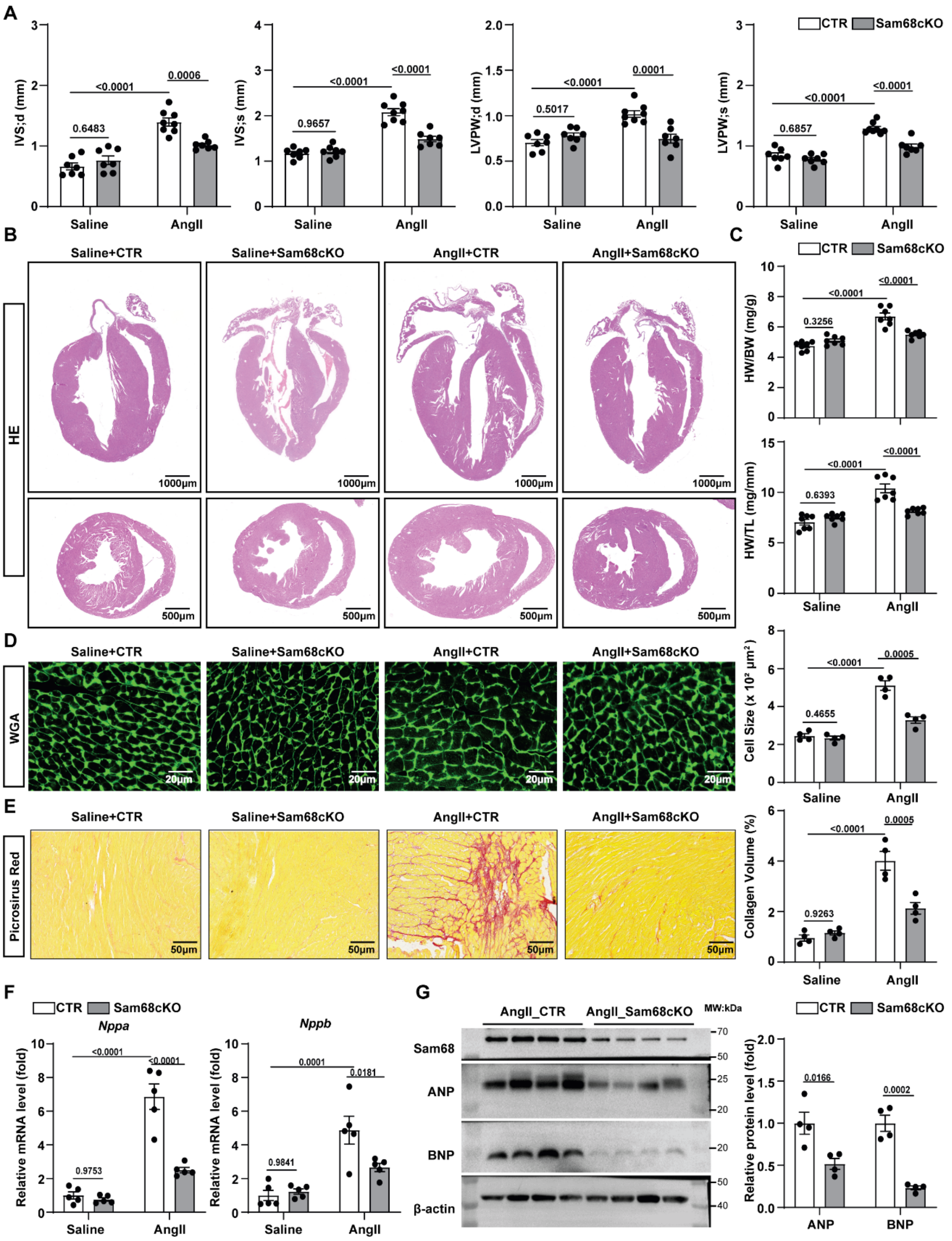

Figure S3. Cardiomyocyte Sam68 deletion attenuates AngII-induced cardiac hypertrophy. Sam68cKO and CTR mice received saline or angiotensin II (AngII; 1.44 mg/kg/day) via osmotic minipumps for 2 weeks. **A,** Echocardiography: IVS;d/IVS;s) and LVPW;d/LVPW;s. **B,** Representative H&E-stained heart sections (longitudinal and

transverse). **C,** Hypertrophy indices (HW/BW and HW/TL; n=7/group). **D,** WGA staining and quantification of cardiomyocyte cross-sectional area (scale bar, 20 μm; n=4/group). **E,** Picrosirius red staining and quantification of collagen volume fraction (scale bar, 50 μm; n=4/group). **F,** Nppa and Nppb mRNA expression (n=5/group). **G,** ANP and BNP protein levels (immunoblots and quantification; n=4/group). Data are mean ± SEM. Statistics: two-way ANOVA with Tukey's multiple-comparisons test (A, C through F) and Sidak's multiple-comparisons test (G).

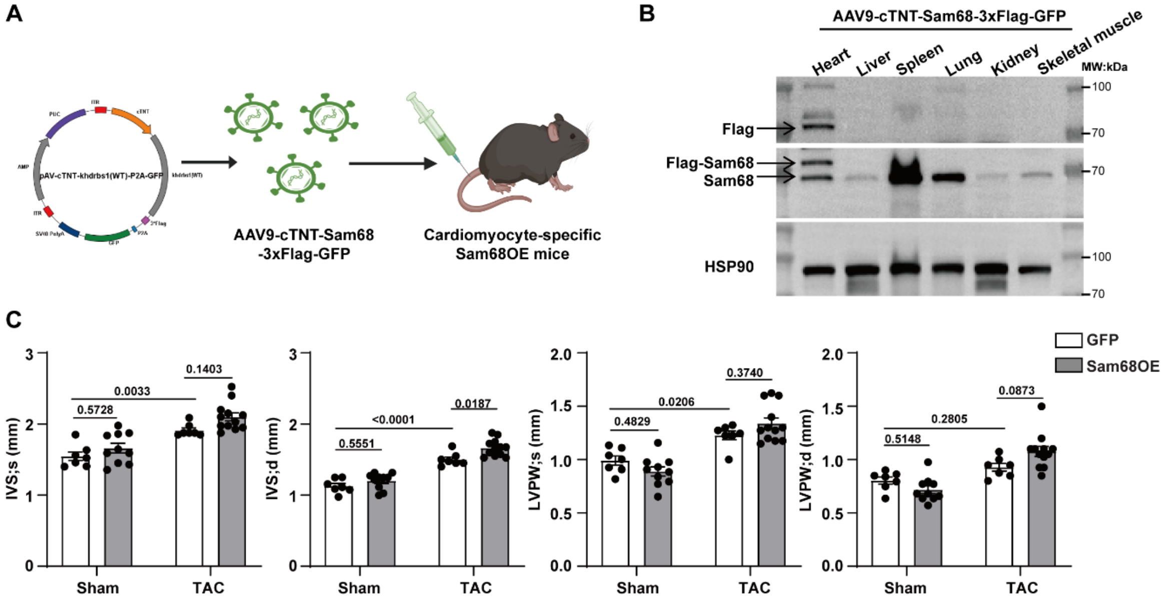

Figure S4. Cardiomyocyte Sam68 overexpression exacerbates TAC-induced hypertrophy. A, Cardiomyocyte-restricted AAV9 delivery via tail-vein injection: AAV9-cTnT–Sam68-3×Flag (Sam68OE) and AAV9-cTnT–GFP control. **B,** Flag and Sam68 expression in heart and indicated non-cardiac tissues collected 4 weeks after AAV9 administration (immunoblots). **C,** Echocardiography at 2 weeks after sham or TAC: IVS;d/IVS;s and LVPW;d/LVPW;s (sham-GFP, n=7; sham-Sam68OE, n=10; TAC-GFP, n=7; TAC-Sam68OE, n=12). Data are mean ± SEM. Statistics: two-way ANOVA with Tukey's multiple-comparisons test (C).

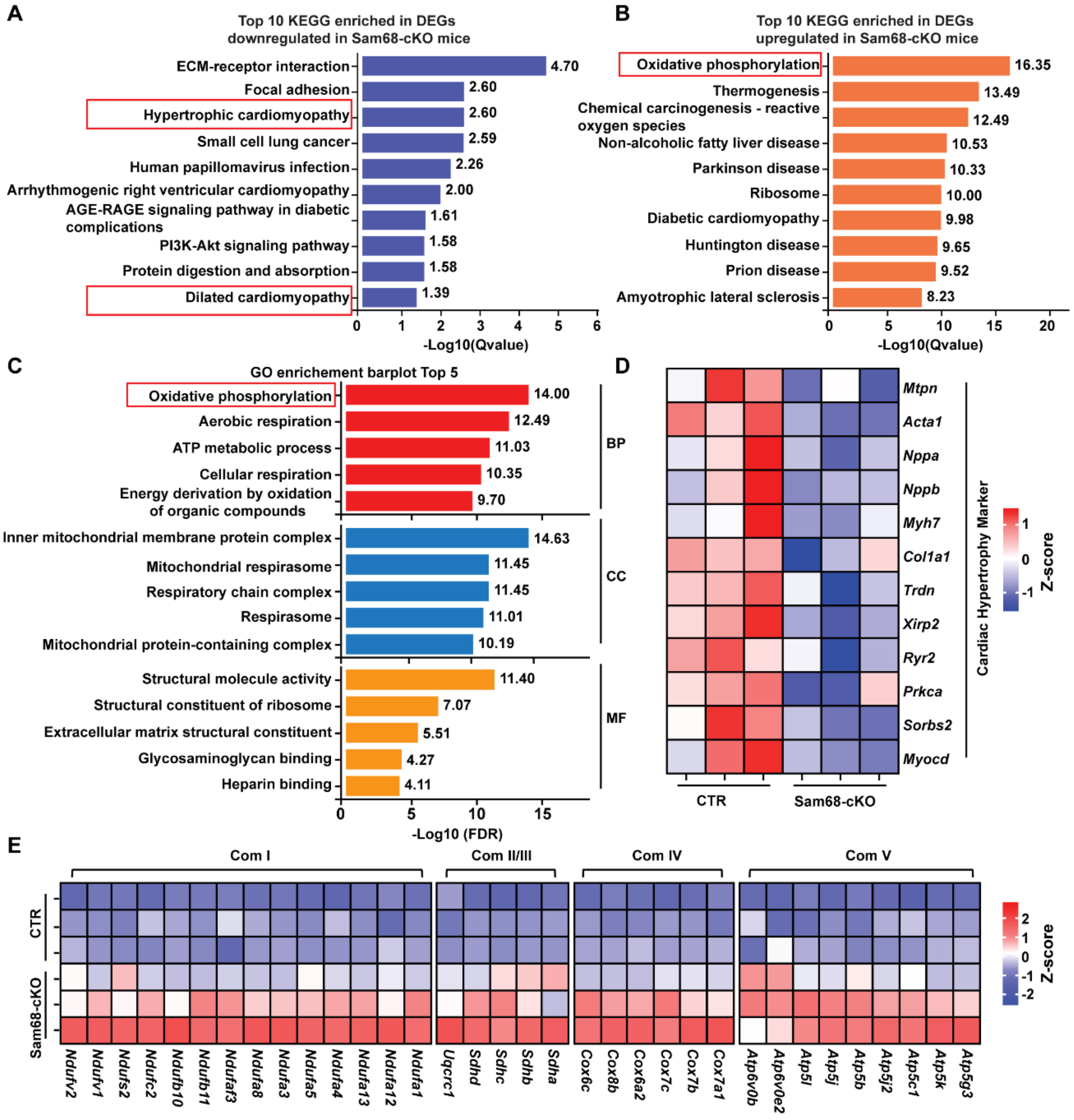

Figure S5. Cardiomyocyte Sam68 deletion preserves oxidative transcriptional programs during hypertrophic stress. Sam68cKO and CTR mice received AngII (1.44 mg/kg/day) for 1 week; hearts were analyzed by RNA-seq. **A and B,** KEGG enrichment for genes upregulated (**A**) or downregulated (**B**) in Sam68cKO vs. CTR hearts. **C,** Gene Ontology (GO) enrichment of differentially expressed genes. **D and E,** Heat maps of hypertrophy-associated markers (**D**) and ETC complex I–V gene sets (**E**). Enrichment significance: hypergeometric test with Benjamini–Hochberg FDR correction; q-values denote Benjamini–Hochberg FDR–adjusted P values (A through C)

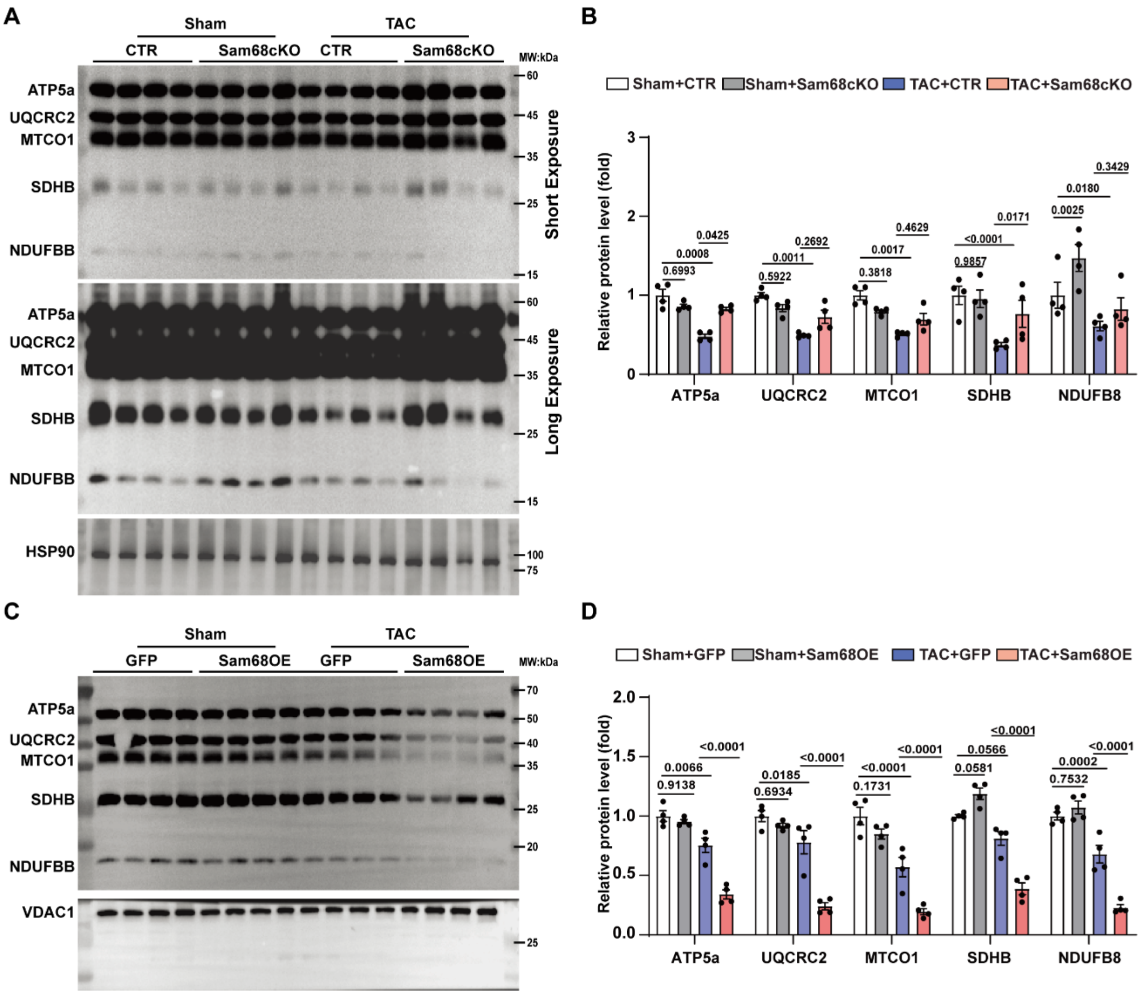

Figure S6. Sam68 suppresses electron transport chain (ETC) protein expression during pressure overload. A and B, ETC complex subunits in CTR and Sam68cKO hearts 4 weeks after sham or TAC (immunoblots and quantification; n=4/group). **C and D,** ETC complex subunits in GFP and Sam68OE hearts 4 weeks after sham or TAC (immunoblots and quantification; n=4/group). Data are mean ± SEM. Statistics: two-way ANOVA with Tukey's multiple-comparisons test (B, D).

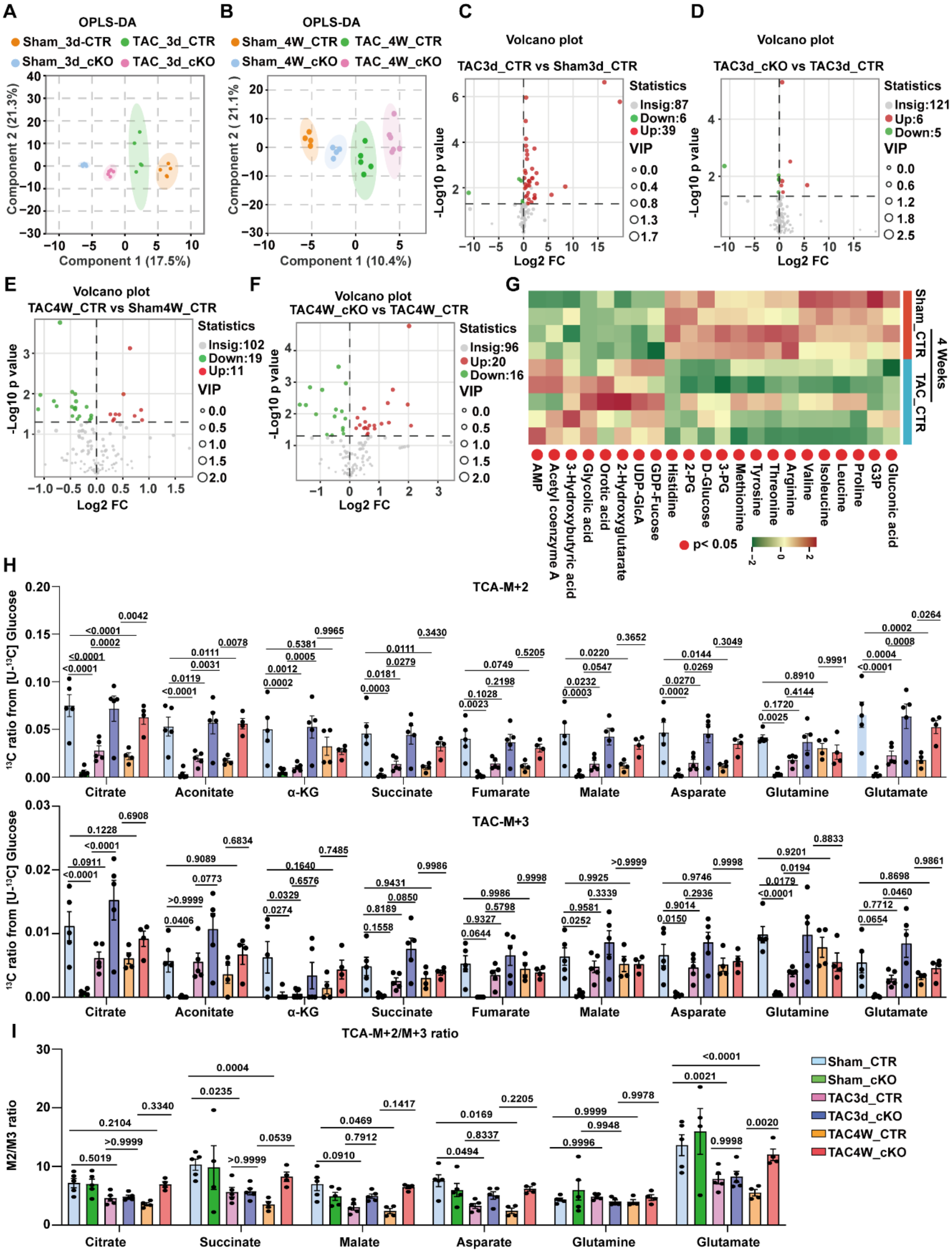

Figure S7. Cardiomyocyte Sam68 deletion reshapes cardiac energy metabolism under pressure overload. A through C, Targeted metabolomics in CTR and Sam68cKO hearts at 3 days after sham or TAC: OPLS-DA score plot (**A**); volcano plot comparing TAC-

CTR vs. sham-CTR (VIP > 1, $P < 0.05$) (**B**); volcano plots comparing TAC-Sam68cKO vs. TAC-CTR (VIP > 1, $P < 0.05$) (**C**). **D through F,** Targeted metabolomics at 4 weeks after sham or TAC: OPLS-DA score plot (**D**); volcano plot comparing TAC-CTR vs sham-CTR (VIP > 1, $P < 0.05$) (**E**); volcano plot comparing TAC-Sam68cKO vs TAC-CTR (VIP > 1, $P < 0.05$) (**F**). **G,** Heat map of significantly altered metabolites (VIP > 1, $p < 0.05$) in TAC-CTR vs. sham-CTR hearts at 4 weeks (sham-CTR, n=4; TAC-CTR, n=5). **H,** Natural-abundance–corrected fractional enrichment of M+2 (upper) and M+3 (lower) isotopologues of the indicated TCA-cycle metabolites (and related amino acids) after in vivo [U-$^{13}C_6$]-glucose tracing. Sample sizes: sham and 3 days post-TAC, n=5/group; 4 weeks post-TAC, n=4/group. **I**, M+2/M+3 ratio for the indicated metabolites (n as in **H**), used as an index of relative PDH-mediated entry (M+2) versus pyruvate carboxylase–mediated anaplerosis (M+3). Data are mean ± SEM. Statistics: two-way ANOVA with Tukey's multiple-comparisons test (H and I).

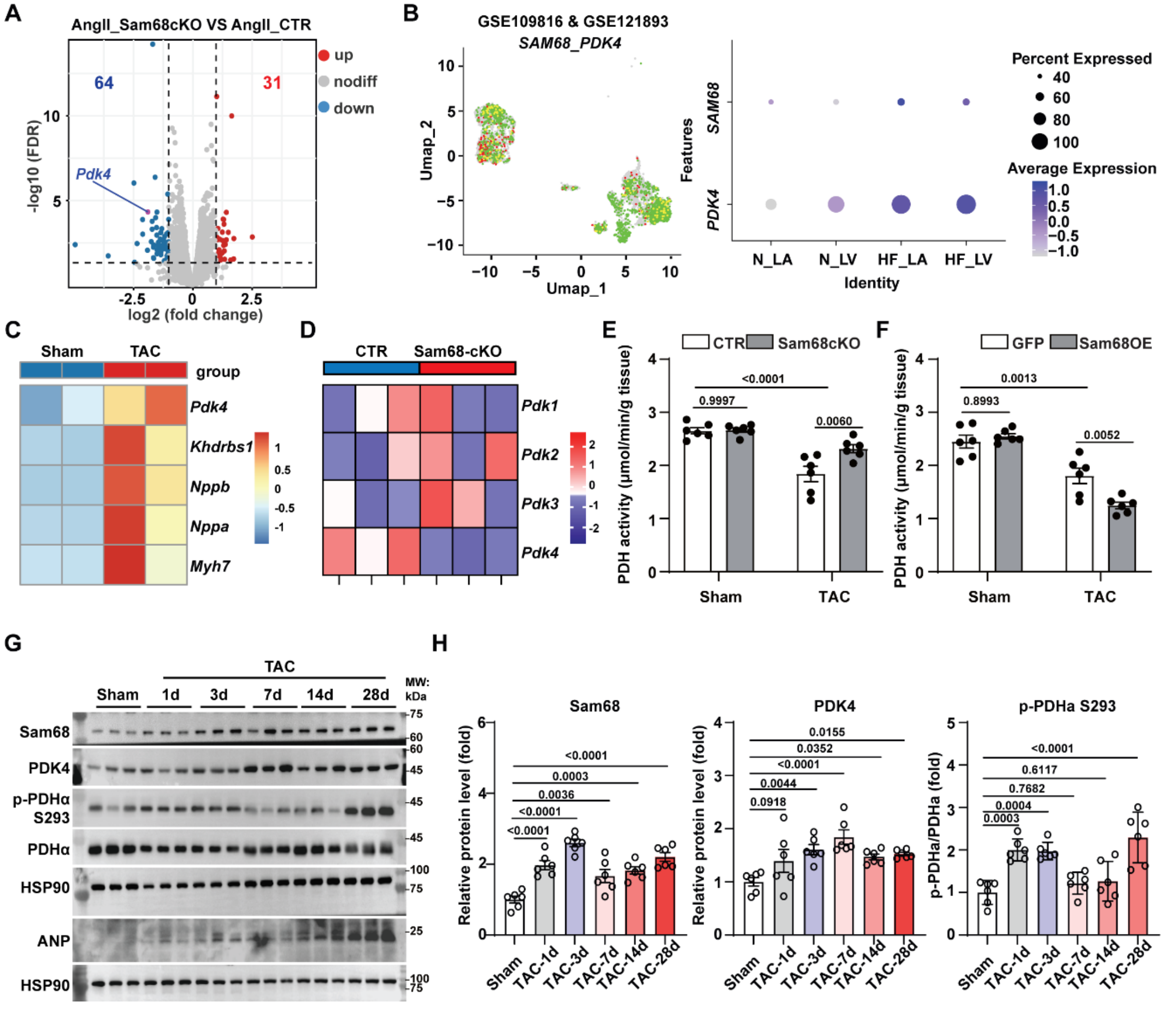

Figure S8. Sam68 and PDK4 are coordinately regulated in mouse and human cardiomyocytes and associate with PDH inhibition. A, Volcano plot of differentially expressed genes in CTR vs. Sam68cKO hearts after 1 week of AngII infusion. **B,** Single-cell RNA-seq dot plot of Sam68 and PDK4 expression in cardiomyocytes from LA and LV tissue of non-failing donors and HF patients (GSE109816 and GSE121893). **C,** Heat map of Khdrbs1 (Sam68), Pdk4, and hypertrophy markers (Nppa, Nppb, Myh7) in isolated adult mouse cardiomyocytes ± 1 week TAC (GSE79883). **D,** Heat map of Pdk1–4 expression in RNA-seq from Ang II–treated CTR vs. Sam68cKO hearts (1 week). **E and F,** PDH activity at 4 weeks after sham or TAC in CTR vs. Sam68cKO hearts (**E**) and GFP vs. Sam68OE hearts (**F**) (n=6/group). **G and H,** Sam68, PDK4, and p-PDHα(Ser293) at indicated times after sham or TAC (immunoblots and quantification; n=6/group). Data are mean ± SEM. Statistics: two-way ANOVA with Tukey's multiple-comparisons test (E and F) or one-way ANOVA with Dunnett's multiple-comparisons test (H).

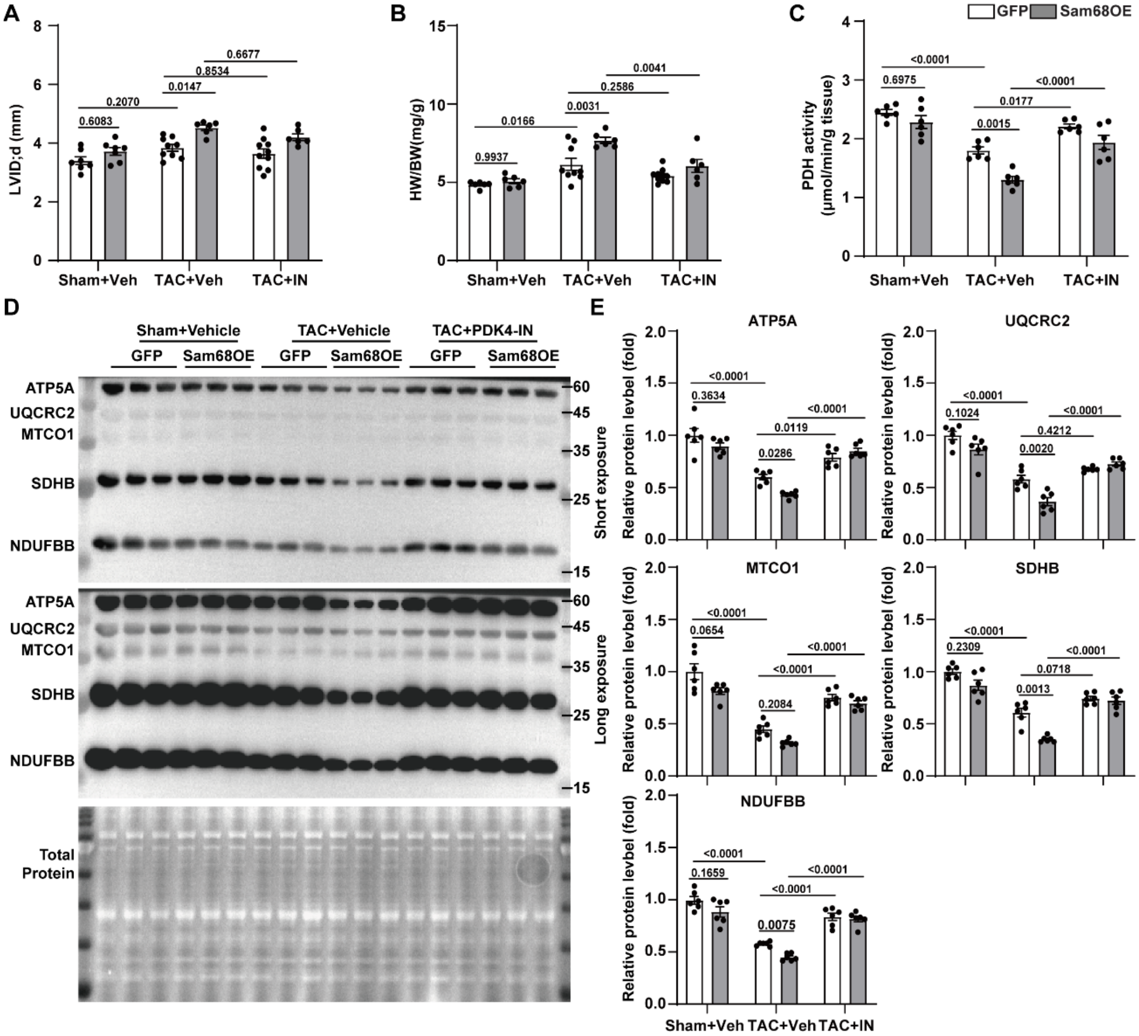

Figure S9. PDK4 inhibition preserves mitochondrial ETC protein expression 4 weeks after TAC. A, Echocardiography (LVID;d) in indicated groups (sham: n=7/group; TAC+GFP+vehicle: n=9; TAC+Sam68OE+vehicle: n=6; TAC+GFP+PDK4-IN: n=9; TAC+Sam68OE+PDK4-IN: n=6). **B,** HW/BW in the same cohorts as (**A**). **C,** PDH activity (n=6/group). **D and E,** ETC complex subunits (immunoblots and quantification; n=4/group). Data are mean ± SEM. Statistics: two-way ANOVA with Tukey's multiple-comparisons test (A through C, E).

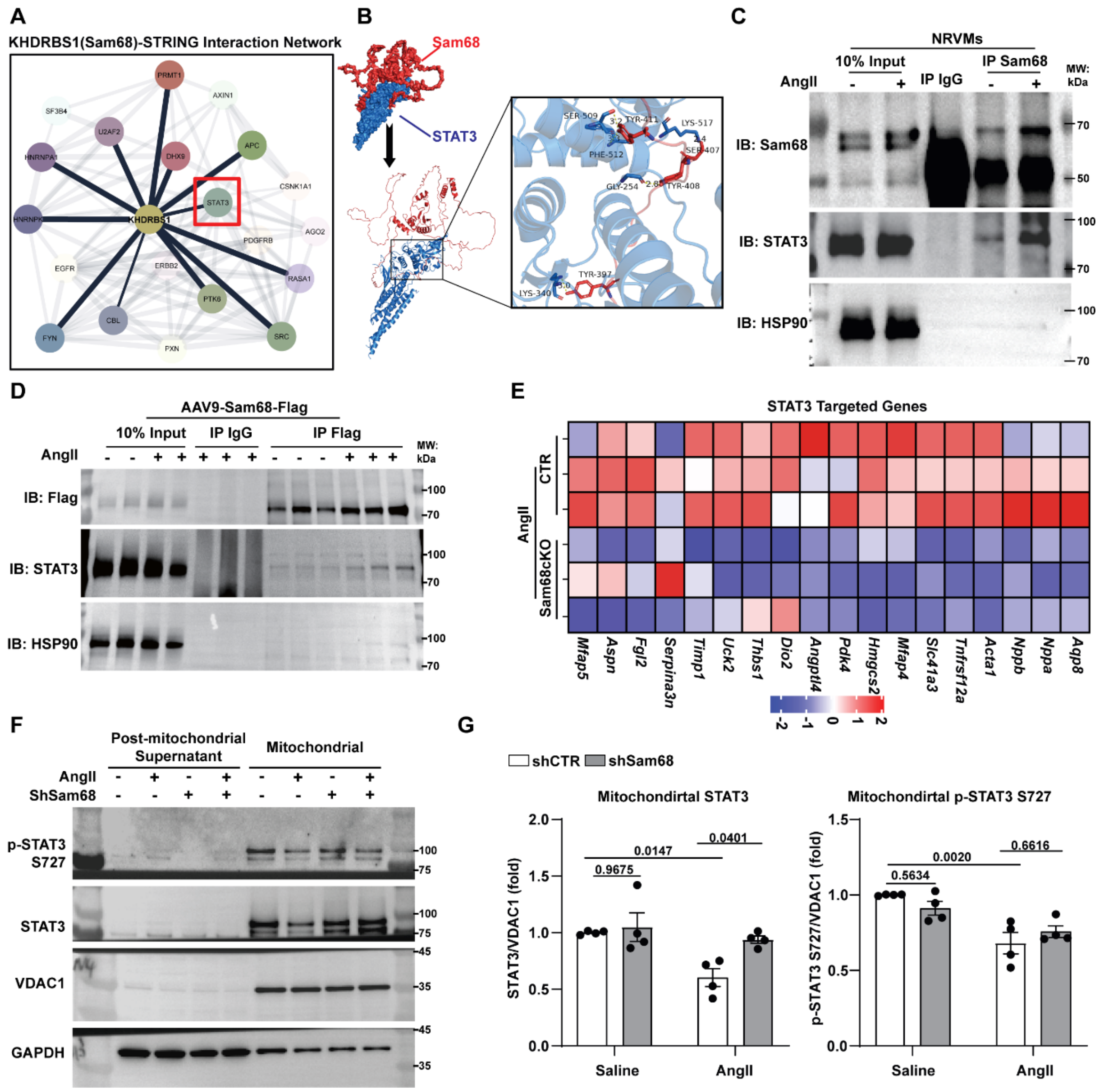

Figure S10. Sam68 interacts with STAT3 in cardiomyocytes and modulates STAT3 compartmental signaling. A, Predicted Sam68 protein–protein interaction network (STRING). **B,** Predicted Sam68–STAT3 binding model (docking). **C,** Endogenous Sam68–STAT3 co-IP in NRVMs treated with saline or AngII (1 μM). **D,** FLAG co-IP from AAV9-Sam68–transduced mouse hearts ± AngII, immunoblotted for STAT3. **E,** STAT3-responsive gene programs (direct targets and/or genes with STAT3-binding promoters) in RNA-seq from AngII-treated CTR vs. Sam68cKO hearts (1 week). **F and G,** NRVMs transduced with adenoviral Sam68 shRNA or non-targeting control and treated with AngII (1 μM, 24 h): mitochondrial and post-mitochondrial fractions (**F**) and quantification of mitochondrial STAT3 and p-STAT3(S727) (**G**) (n=4/group). Data are mean ± SEM. Statistics: two-way ANOVA with Tukey's multiple-comparisons test (G).

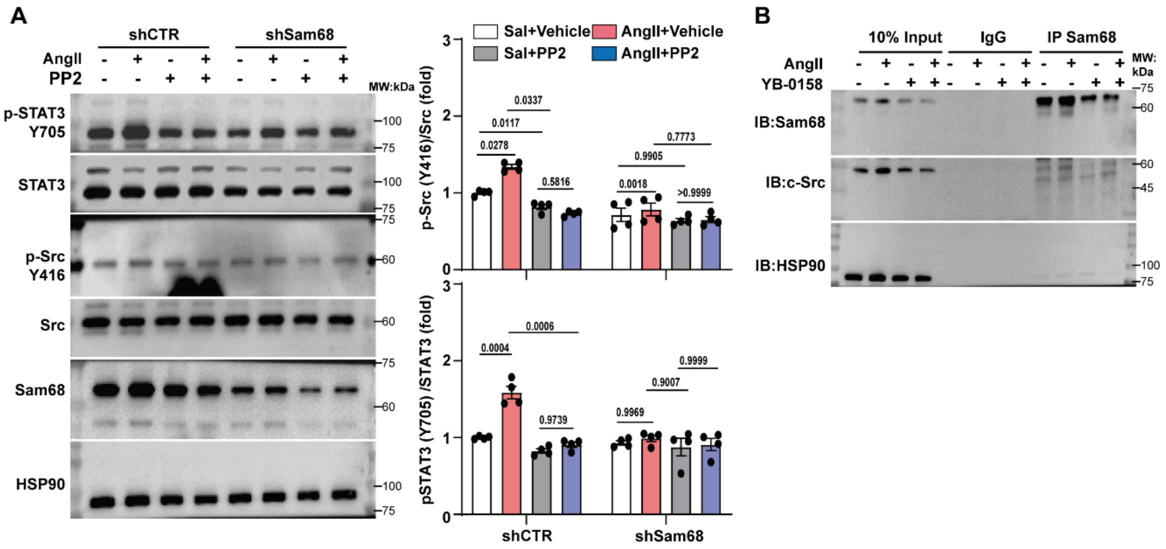

Figure S11. Sam68 promotes Src-dependent STAT3 activation by facilitating the Src–STAT3 interaction. A, p-STAT3(Y705)/STAT3 and p-Src(Y416)/Src in NRVMs transduced with control shRNA (Adv-shCTR) or Sam68 shRNA (Adv-shSam68) and treated with AngII (1 μM) and/or the Src inhibitor PP2 (1 μM) for 24 h (immunoblots and quantification; n=4/group). **B,** Sam68–Src co-IP in NRVMs treated with vehicle or YB-0158 (1 μM) ± AngII (1 μM, 24 h). Data are mean ± SEM. Statistics: three-way ANOVA with Tukey's multiple-comparisons test (A).

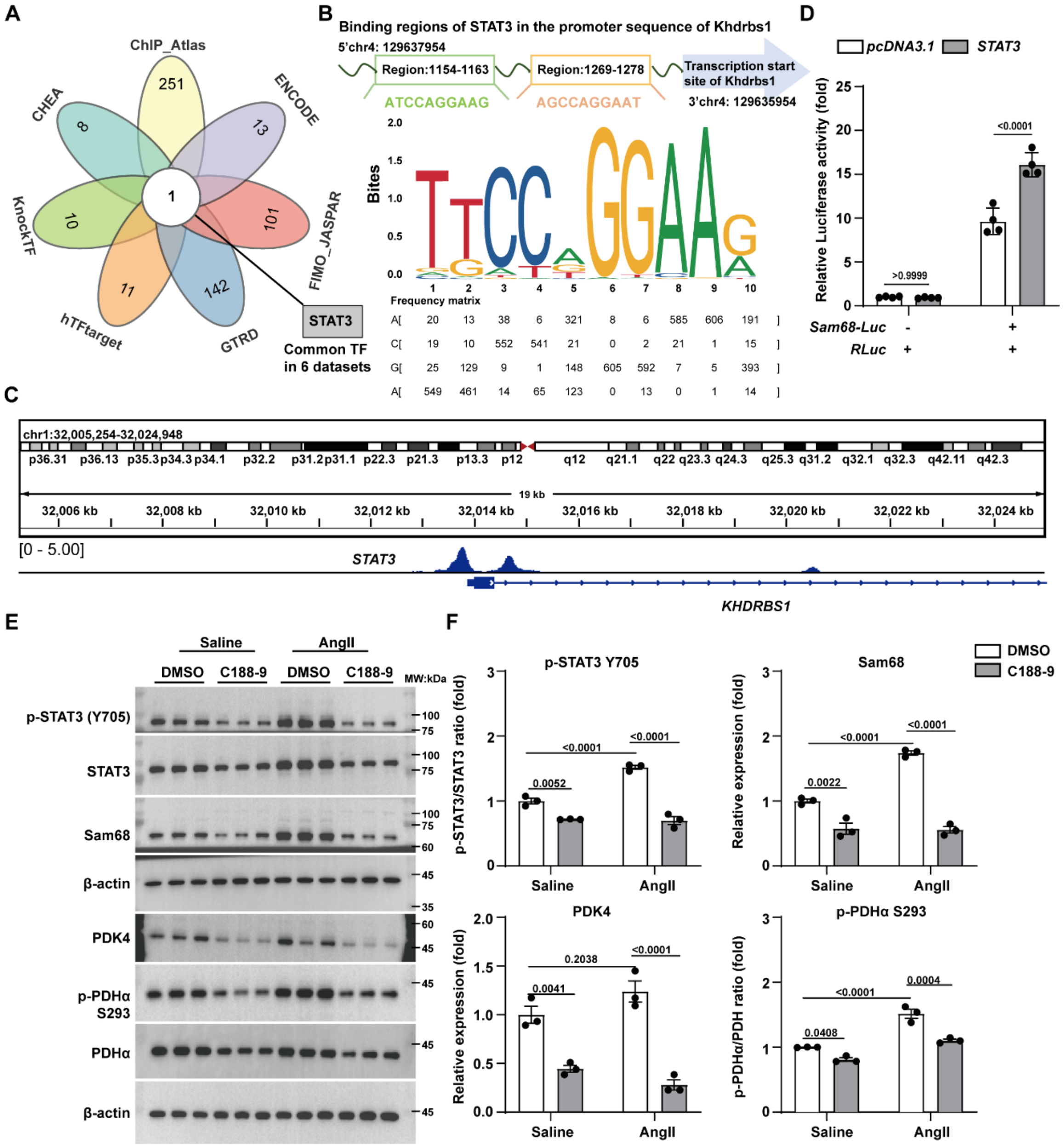

Figure S12. AngII induces Sam68 transcription through STAT3 activation. A, Integrative bioinformatic analysis identifying STAT3 as a top predicted upstream transcriptional regulator of Sam68. **B,** Predicted STAT3-binding motifs within the Sam68 promoter region. **C,** STAT3 ChIP-seq (GSE85579) showing STAT3 occupancy at the Sam68 locus near the transcription start site (TSS). **D,** Dual-luciferase reporter assay (HEK293T) showing STAT3-dependent activation of the Sam68 promoter (firefly/Renilla; fold vs. empty vector). **E and F,** NRVMs treated 24 h with saline, AngII (1 μM), C188-9 (10 μM), or AngII + C188-9: immunoblots (**E**) and quantification (**F**) of p-STAT3(Y705)/STAT3, Sam68, PDK4, and p-PDHα(S293)/PDHα. Data are mean ± SEM. Statistics: two-way ANOVA with Tukey's multiple-comparisons test (D, F).

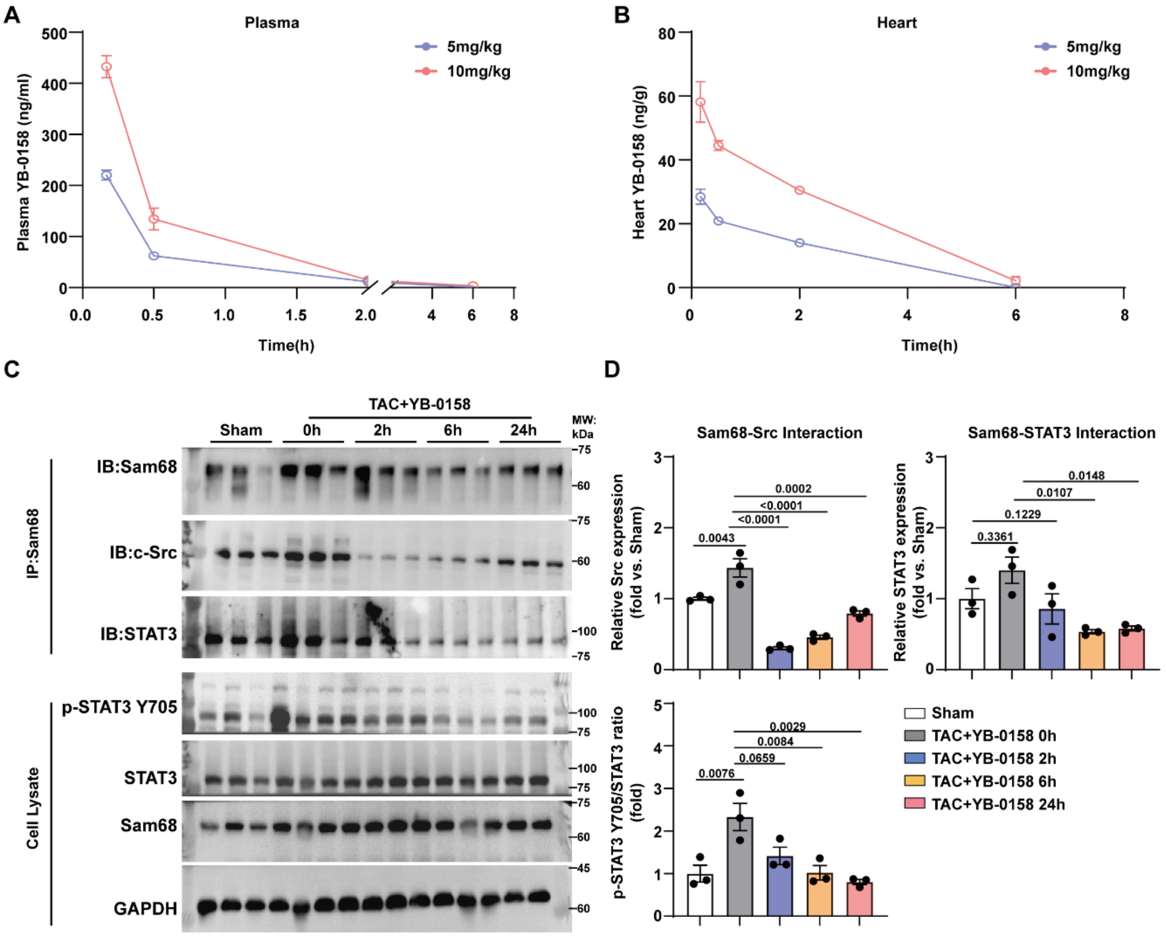

Figure S13. Pharmacokinetics and cardiac target engagement of YB-0158 in mice. A and B, Single i.p. dose of YB-0158 (5 or 10 mg/kg) in C57BL/6J mice; concentrations in plasma (**A**) and heart (**B**) at indicated time points (n=6 per time point and dose). **C and D,** Sham or TAC surgery; 1 day later, TAC mice received YB-0158 (5 mg/kg, i.p.) and hearts were collected at 0, 2, 6, and 24 h post-dose. Sam68–Src and Sam68–STAT3 interactions (co-IP) and p-STAT3(Y705) in cardiac lysates are shown (n=3 per time point). Data are mean ± SEM. Statistics: one-way ANOVA with Tukey's multiple-comparisons test (D).

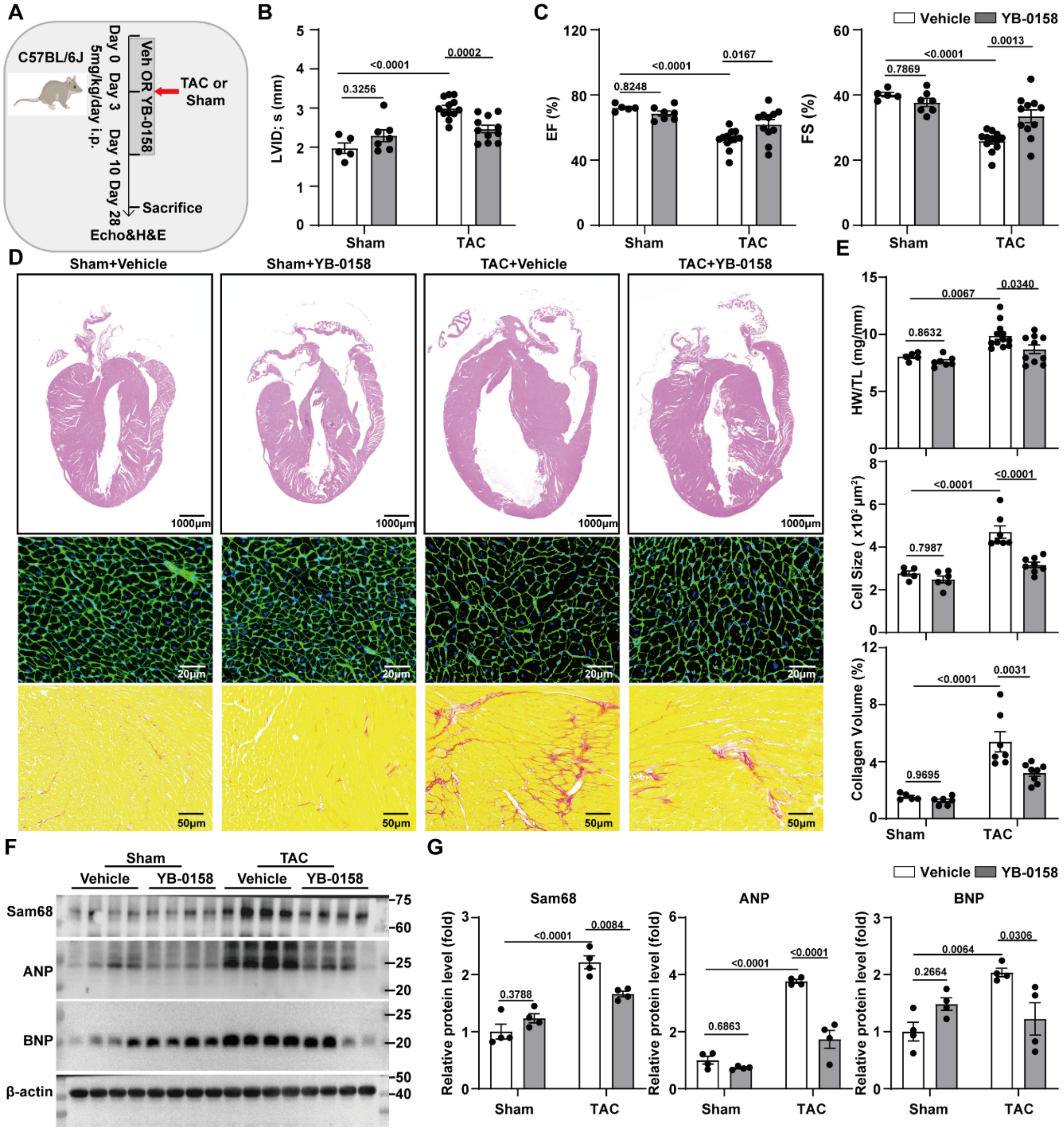

Figure S14. YB-0158 prevents TAC-induced hypertrophy. A, Preventive regimen: daily i.p. YB-0158 (5 mg/kg/day) or vehicle for 3 days before sham/TAC and for 7 days after surgery; endpoints assessed 4 weeks post-operation. **B and C,** Echocardiography: LVID;s (**B**) and EF/FS (**C**). **D,** Representative H&E, WGA, and picrosirius red staining. **E,** Quantification: HW/TL (sham+vehicle n=5, sham+YB-0158 n=7, TAC+vehicle n=12, TAC+YB-0158 n=11), cardiomyocyte cross-sectional area and collagen volume fraction (sham+vehicle n=5, sham+YB-0158 n=6, TAC+vehicle n=7, TAC+YB-0158 n=8). **F and G,** Sam68, ANP, and BNP (immunoblots and quantification; n=4/group). Data are mean ± SEM. Statistics: two-way ANOVA with Tukey’s multiple-comparisons test (B, C, E, G).

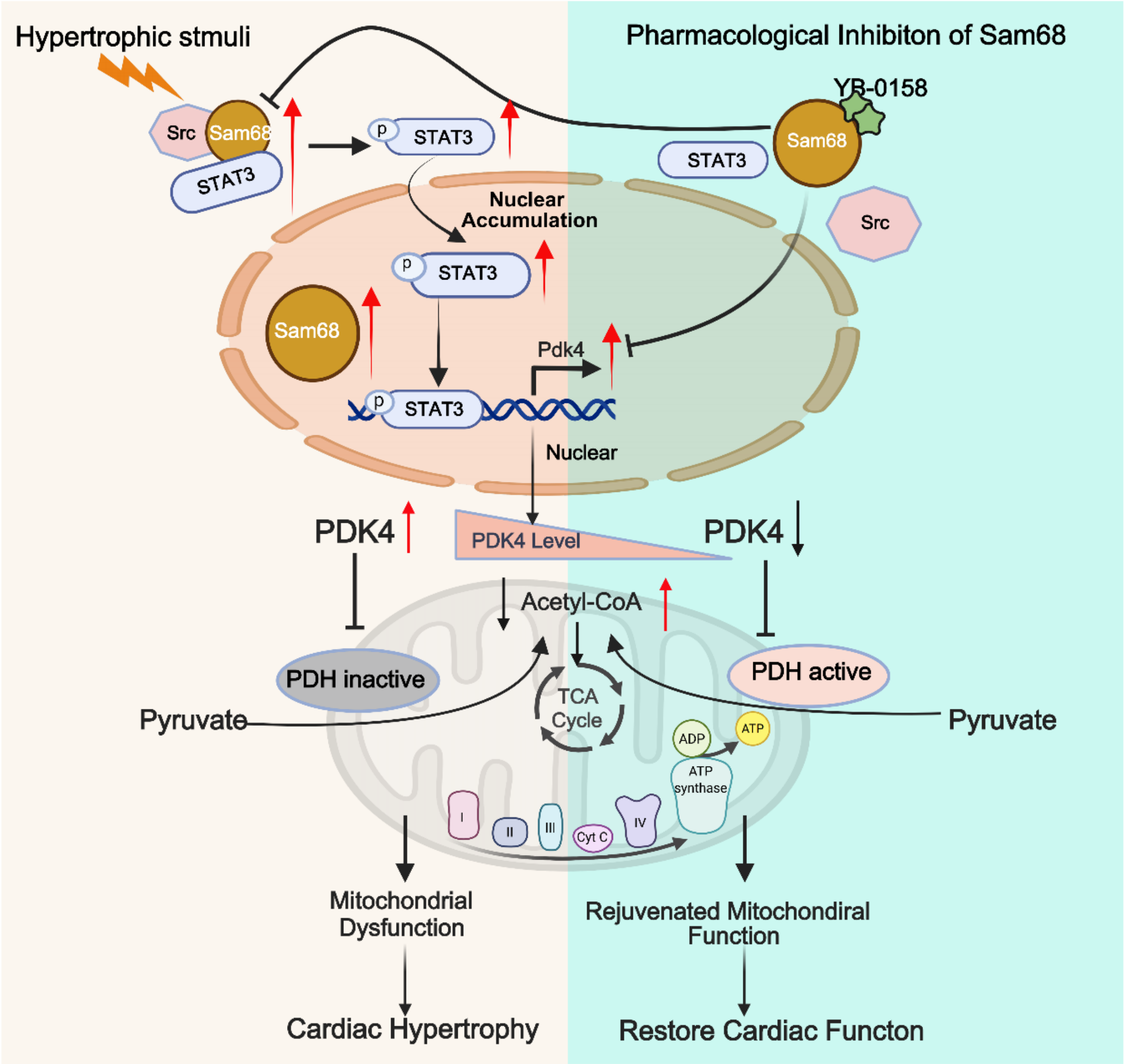

Figure S15. Graphical Abstract. Proposed model of Sam68-driven metabolic remodeling in pathological hypertrophy.

Hypertrophic stress induces cardiomyocyte Sam68, which scaffolds Src and STAT3 to promote STAT3 Tyr705 phosphorylation, nuclear accumulation, and transcriptional activation of Pdk4. Increased PDK4 inhibits PDH, suppressing pyruvate oxidation and uncoupling glycolysis from mitochondrial glucose oxidation, thereby exacerbating hypertrophic remodeling. Genetic deletion of Sam68 or pharmacologic disruption of the Src–Sam68–STAT3–PDK4 axis restores PDH-dependent glucose oxidation and improves cardiac remodeling and function.

Supplemental Excel File Legend

Excel File S1. Raw Metabolite Peak Areas and Isotopologue Distributions from *In Vivo* [U-^{13}C]-Glucose Tracing

This file provides raw mass spectrometry data from *in vivo* [U-^{13}C]-glucose metabolic flux analysis in mice subjected to transverse aortic constriction (TAC) at 3 days and 4 weeks post-surgery.

Sheet ("13C-Glucose Flux Total Ion"): Raw total ion peak areas for all detected metabolic intermediates.

Sheet ("Raw Table for Isotopologue"): Raw peak areas for individual mass isotopologues (M+0 to M+n) of each metabolite. These values were used to calculate fractional ^{13}C enrichment and metabolic flux ratios presented in the main figures.

Data are shown for cardiomyocyte-specific Sam68 knockout (Sam68cKO) and control (CTR) mice at both time points.
