# Supplementary material for: Sam68 Exacerbates Pathologic Cardiac Hypertrophy by Suppressing Cardiomyocyte Glucose Oxidation
Source: Circulation. 2026 May 22;153(25):2044–63. doi: 10.1161/CIRCULATIONAHA.125.077533 (PMC13286121; doi:10.1161/CIRCULATIONAHA.125.077533)

Full unedited gel for Figure 1F

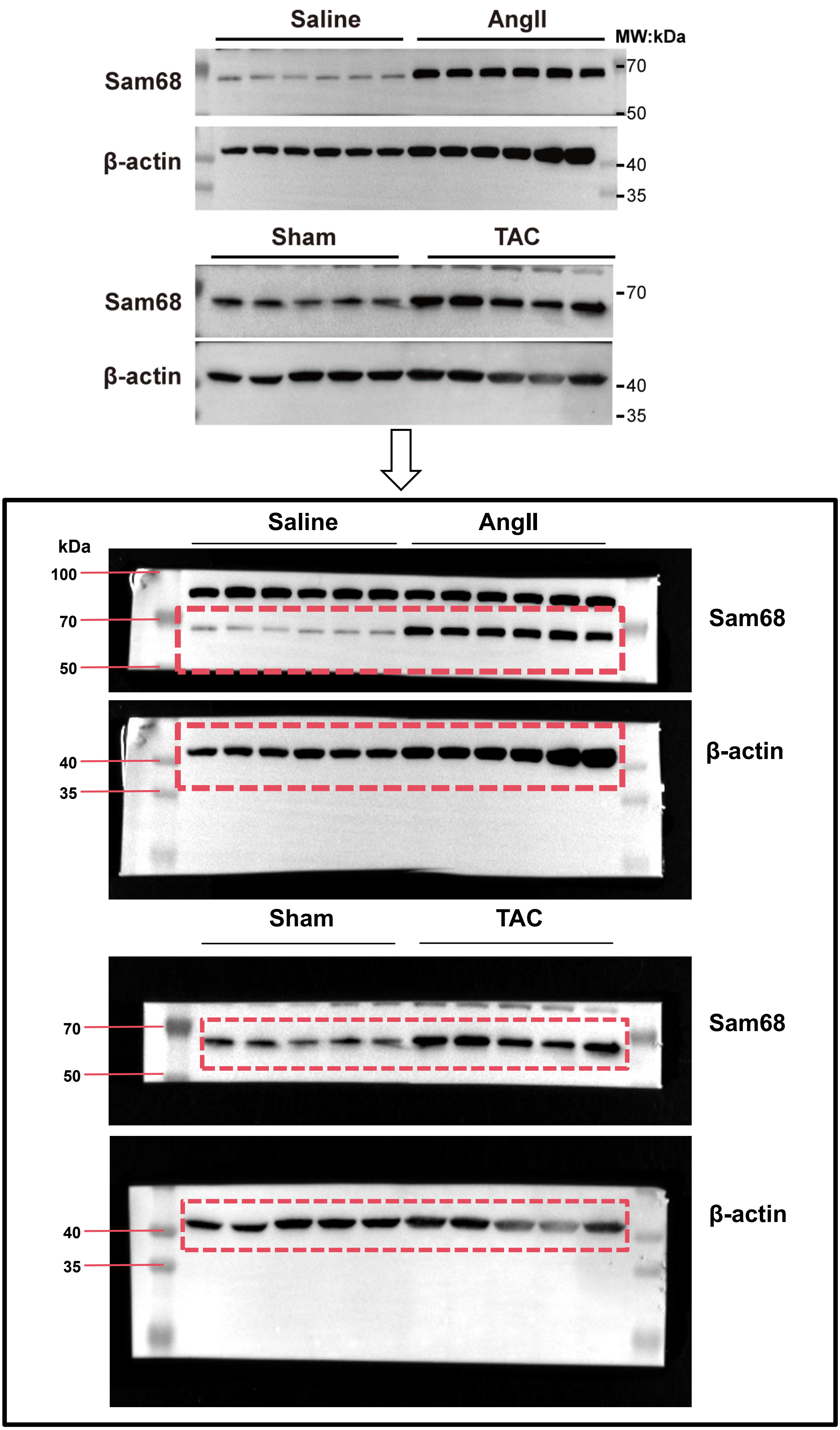

Full unedited gel for Figure 2G

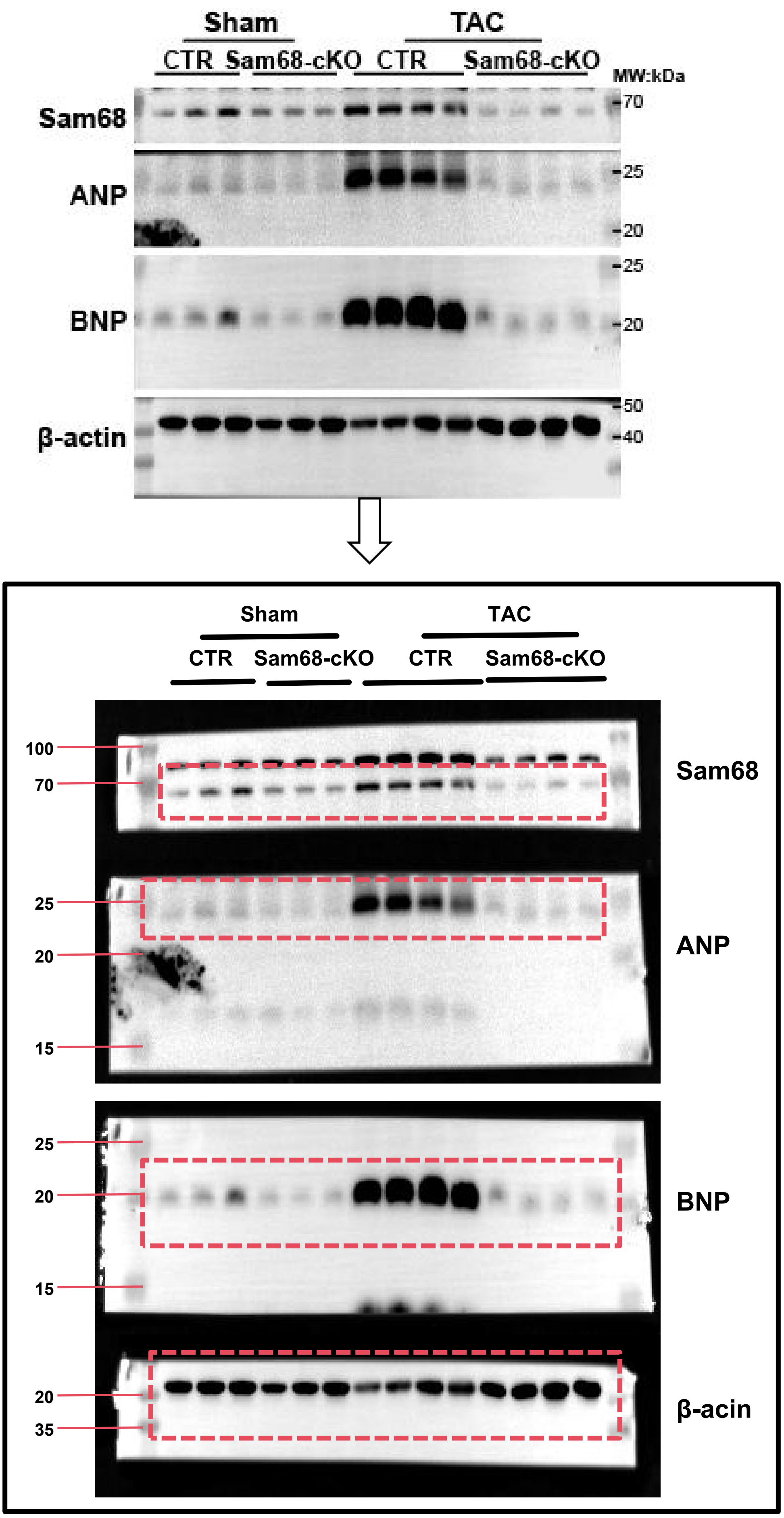

Full unedited gel for Figure 3H

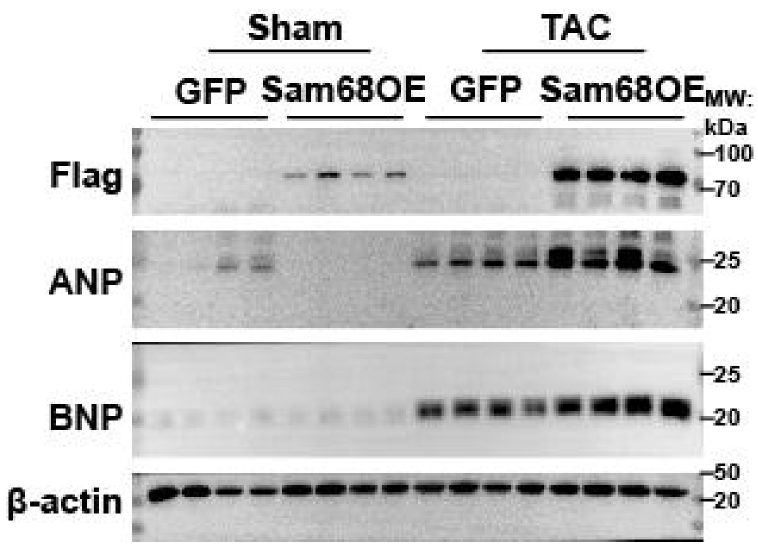

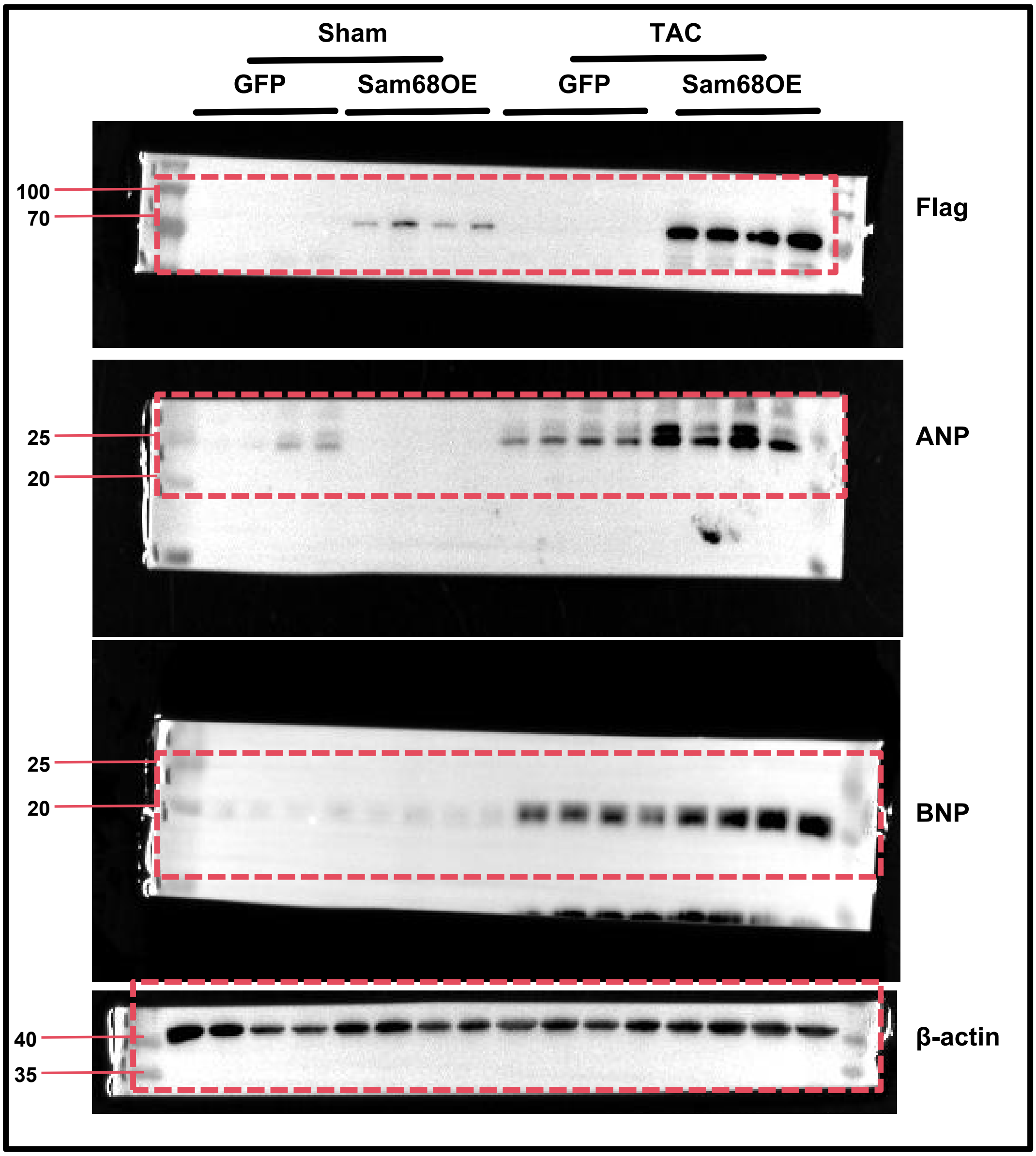

Full unedited gel for Figure 4F

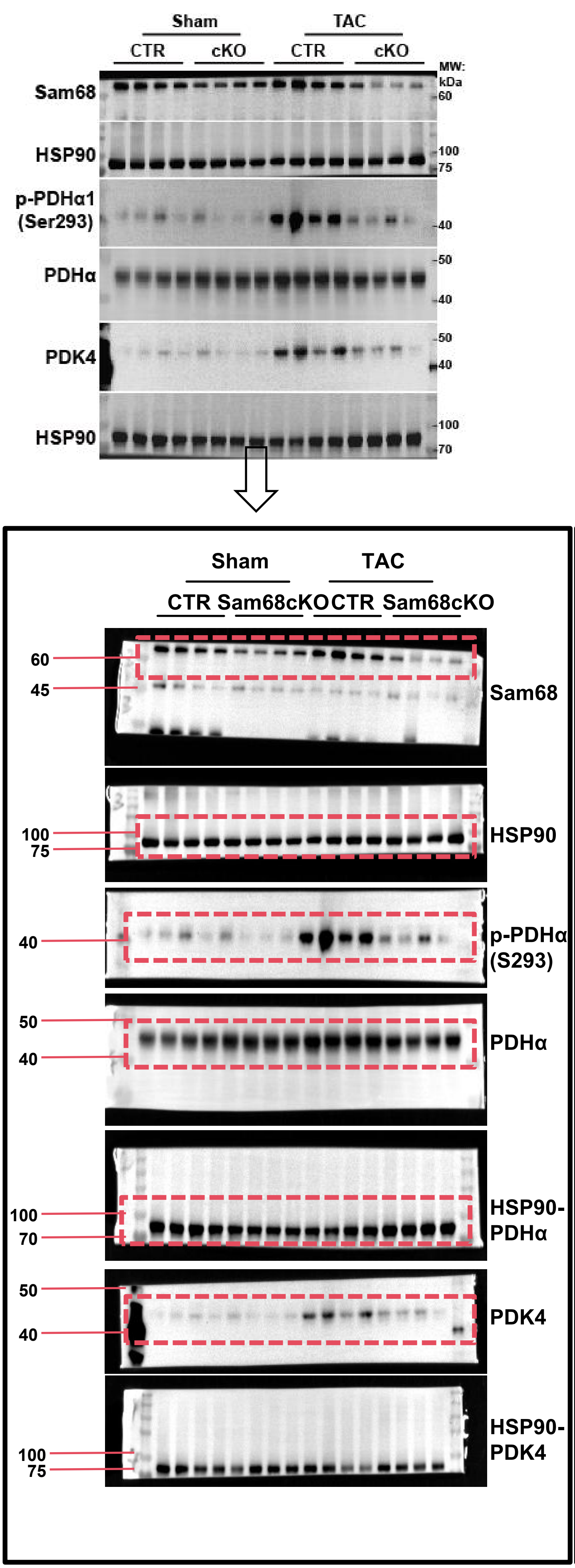

Full unedited gel for Figure 4G

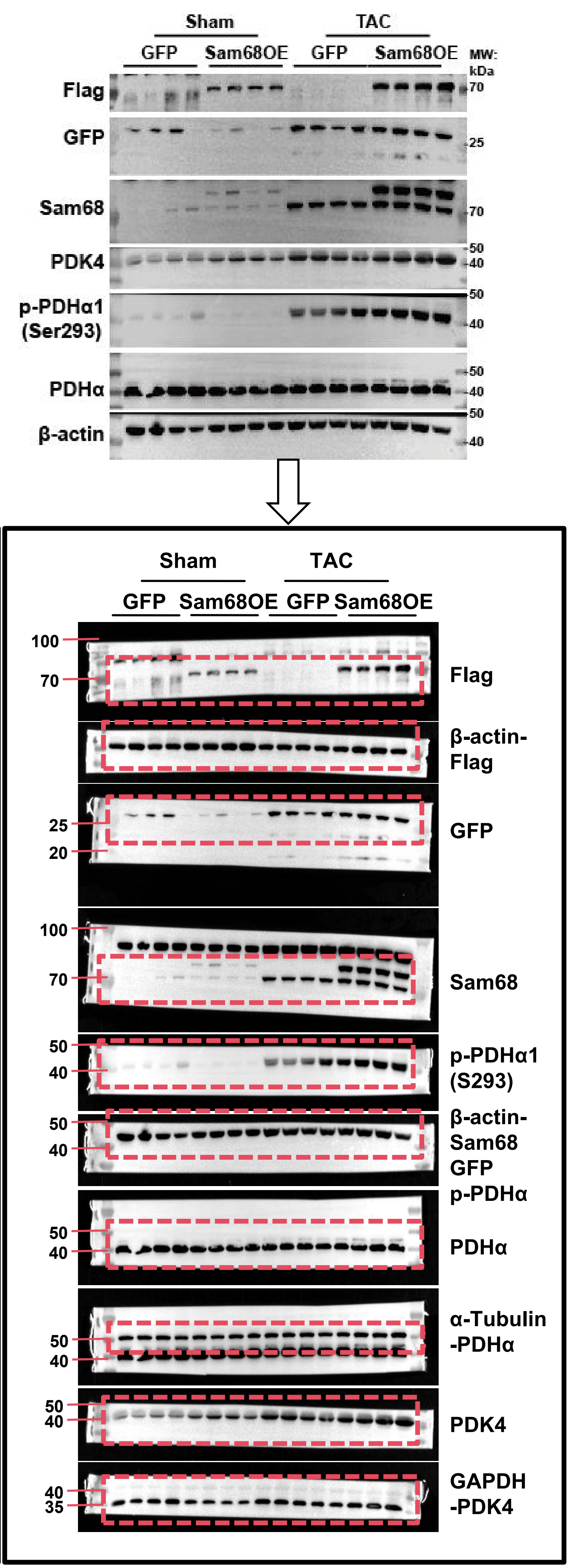

Full unedited gel for Figure 5H

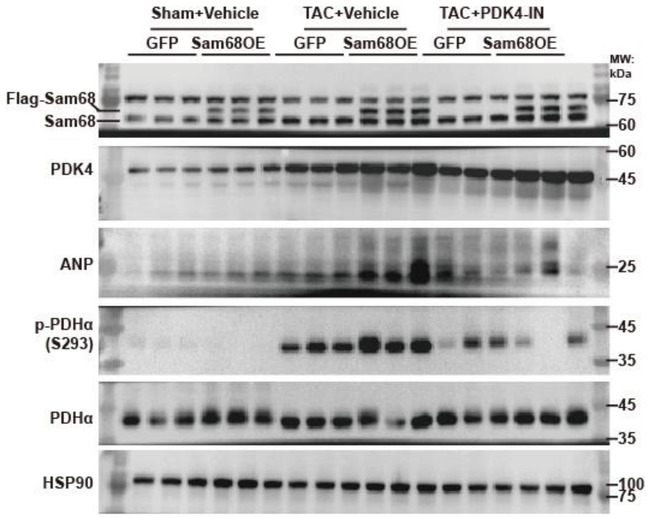

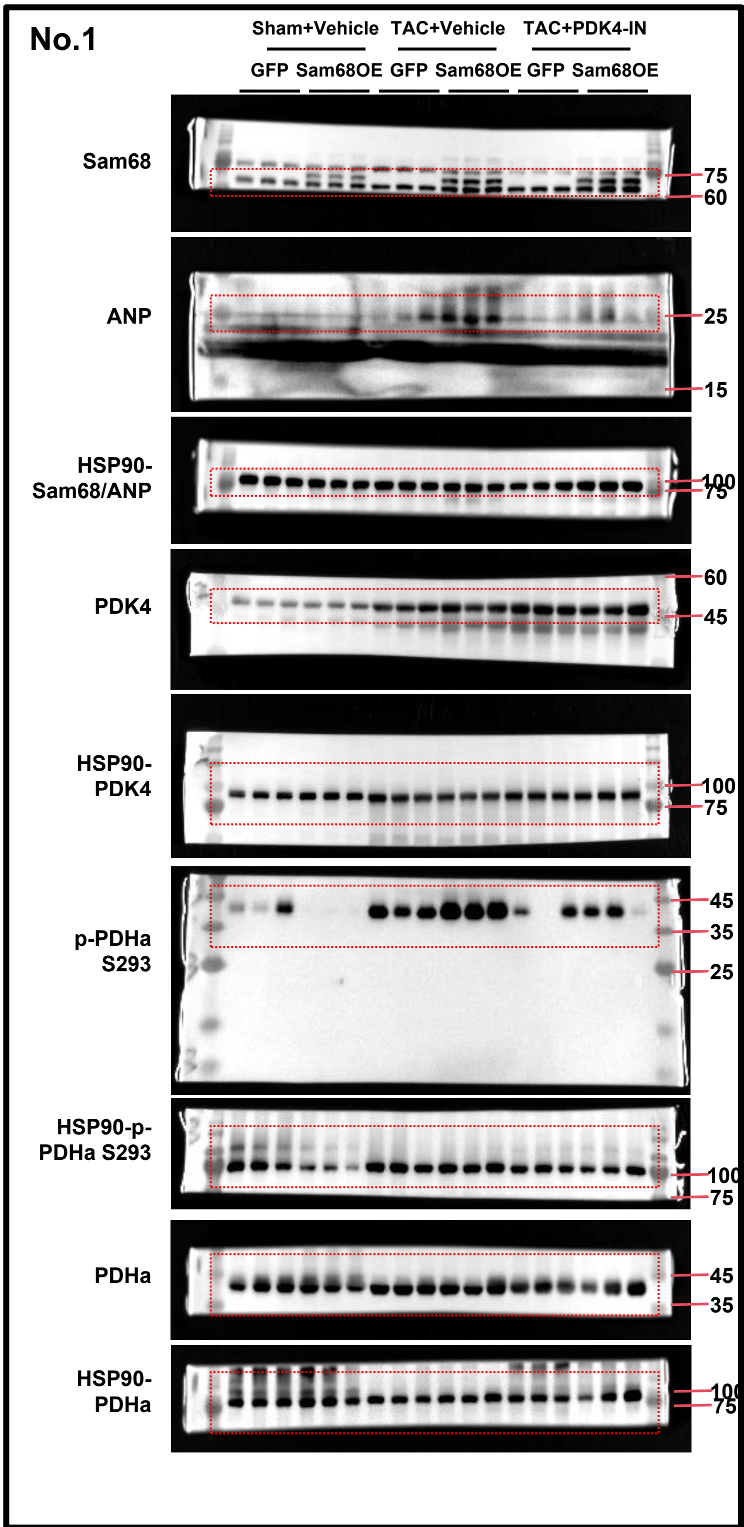

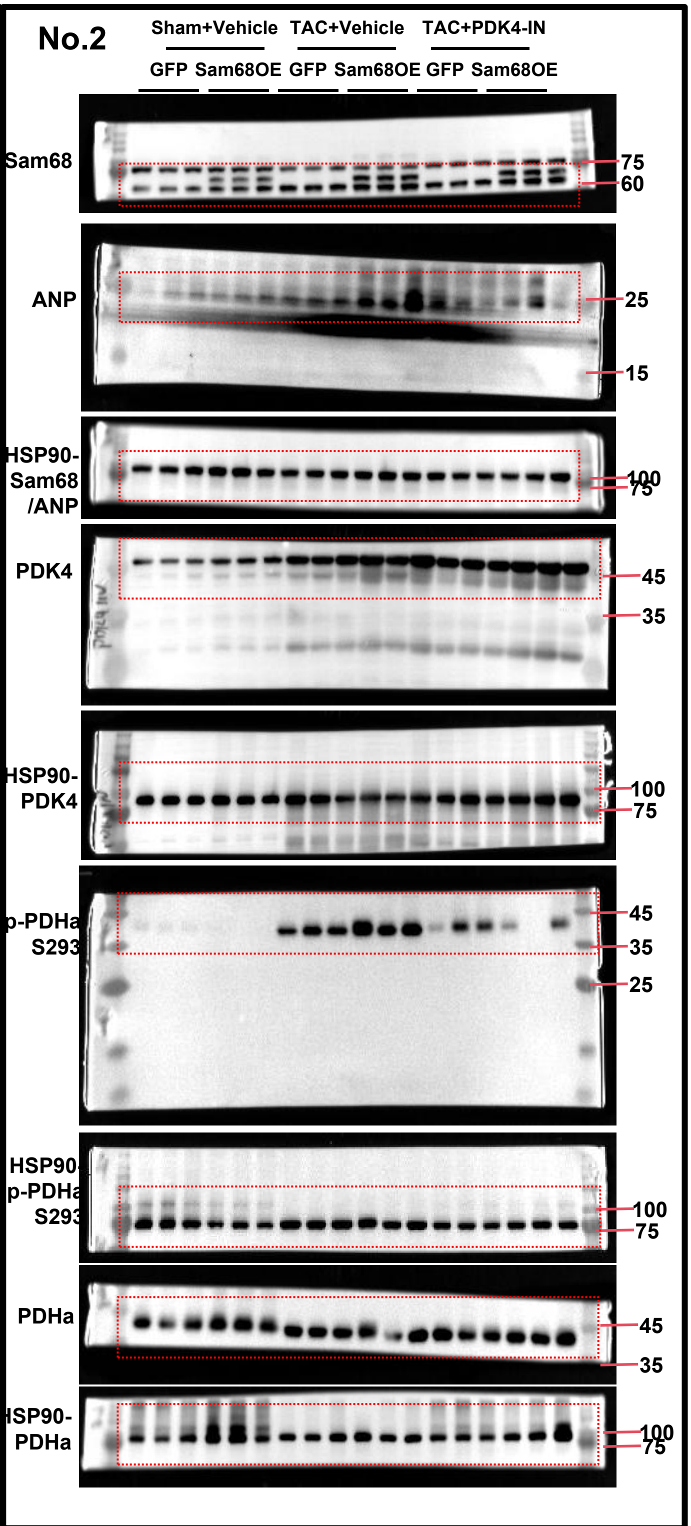

Full unedited gel for Figure 6A **Full unedited gel for Figure 6B**

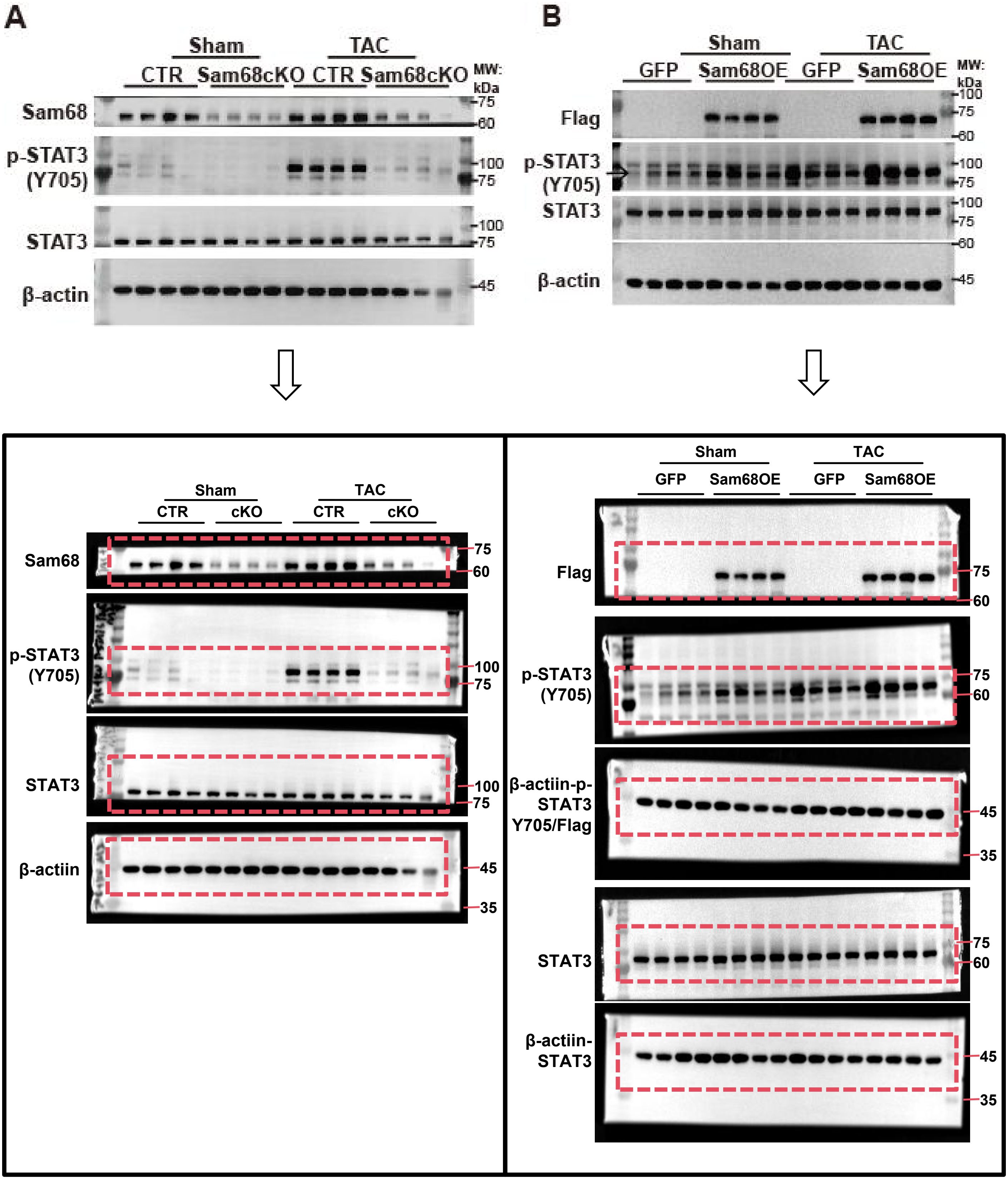

Full unedited gel for Figure 6C

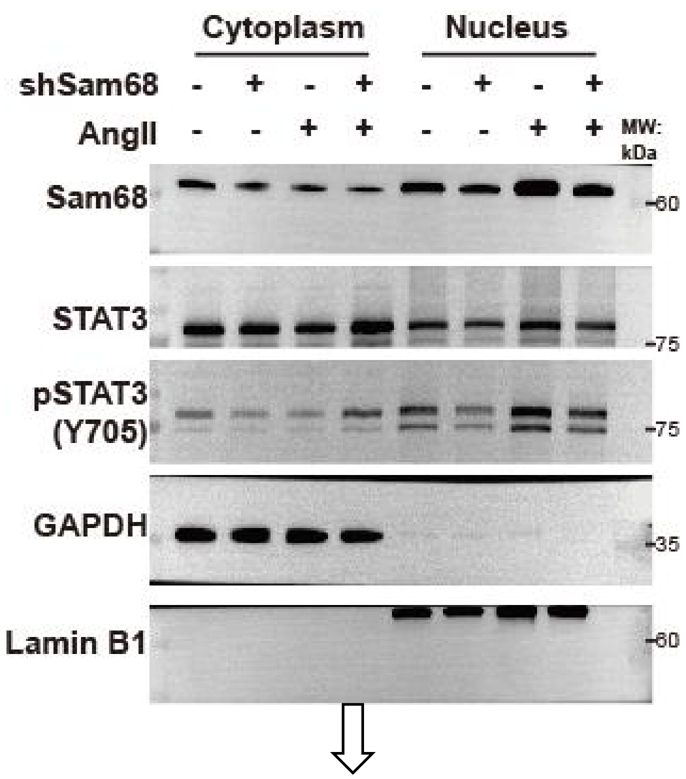

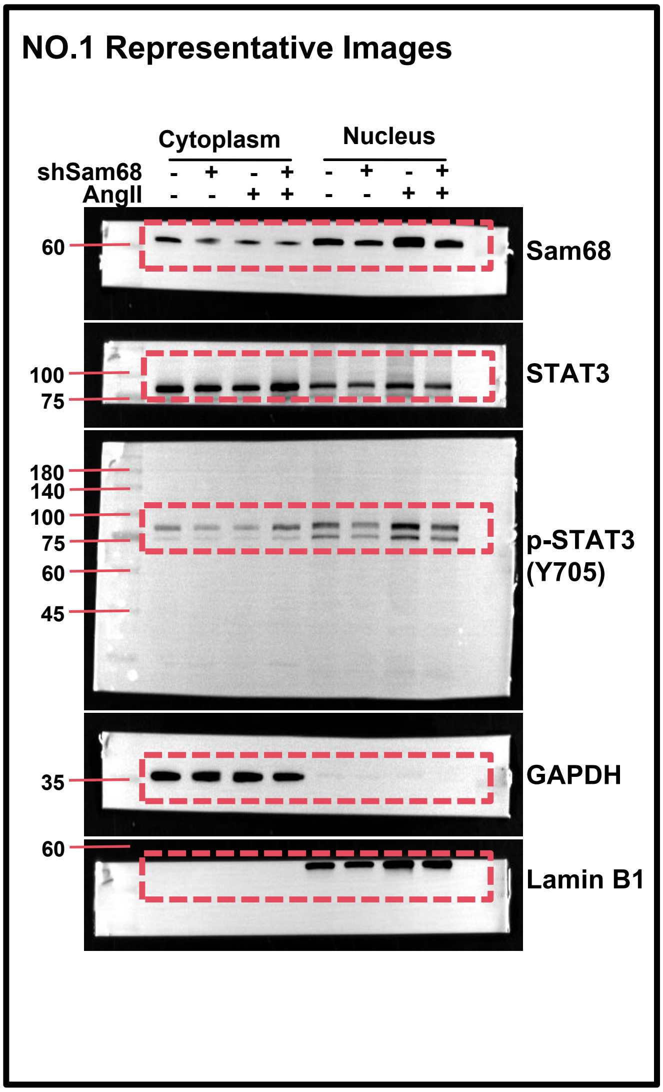

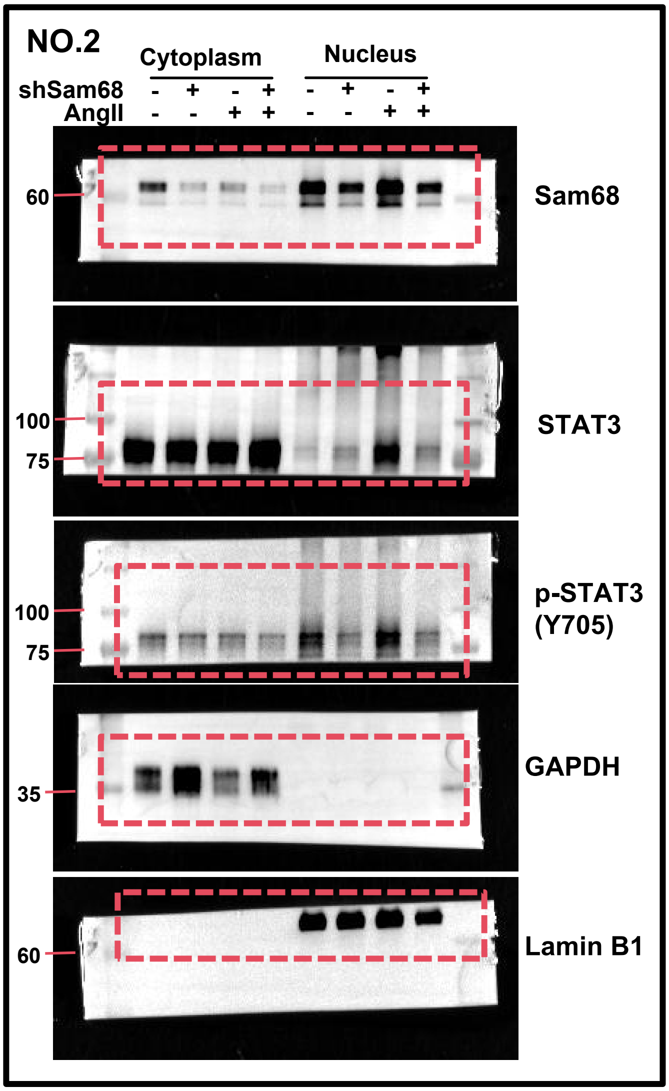

Full unedited gel for Figure 6C

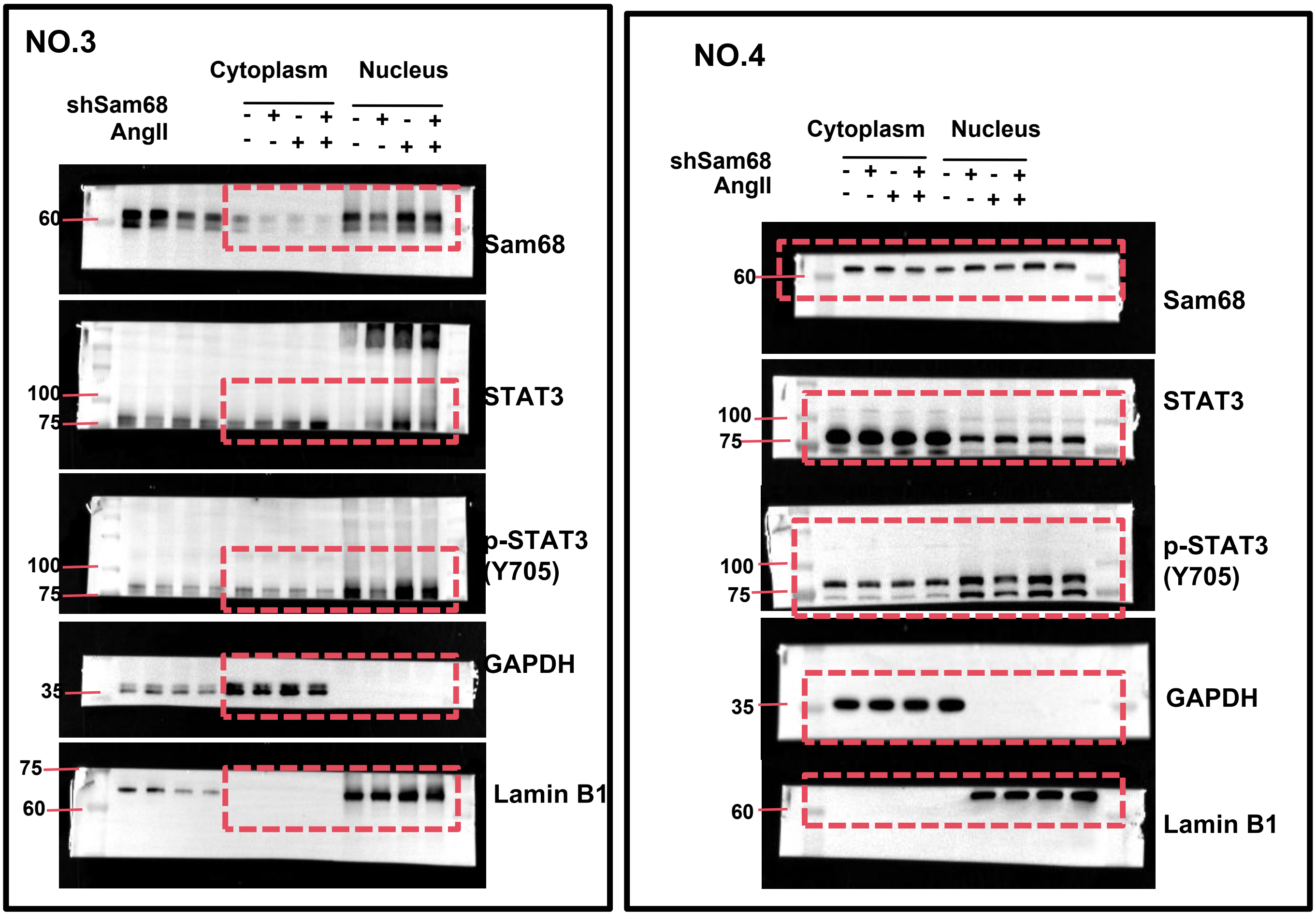

Full unedited gel for Figure 6D

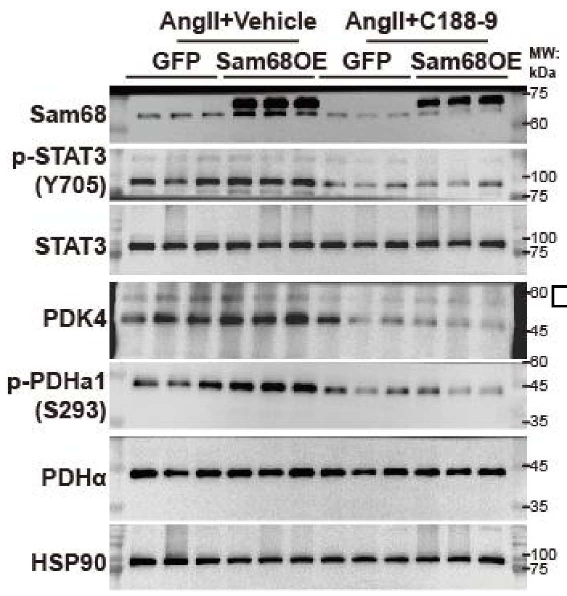

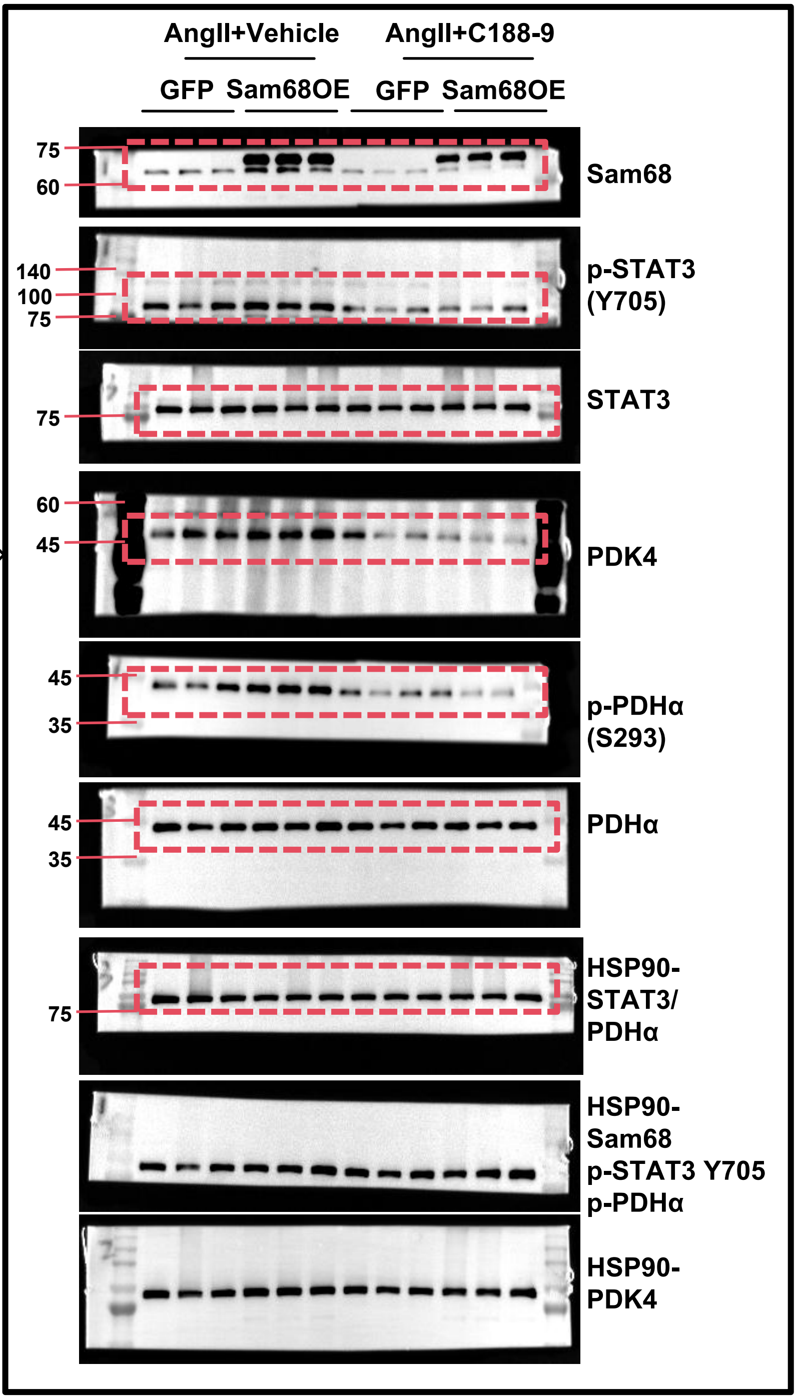

Full unedited gel for Figure 6F

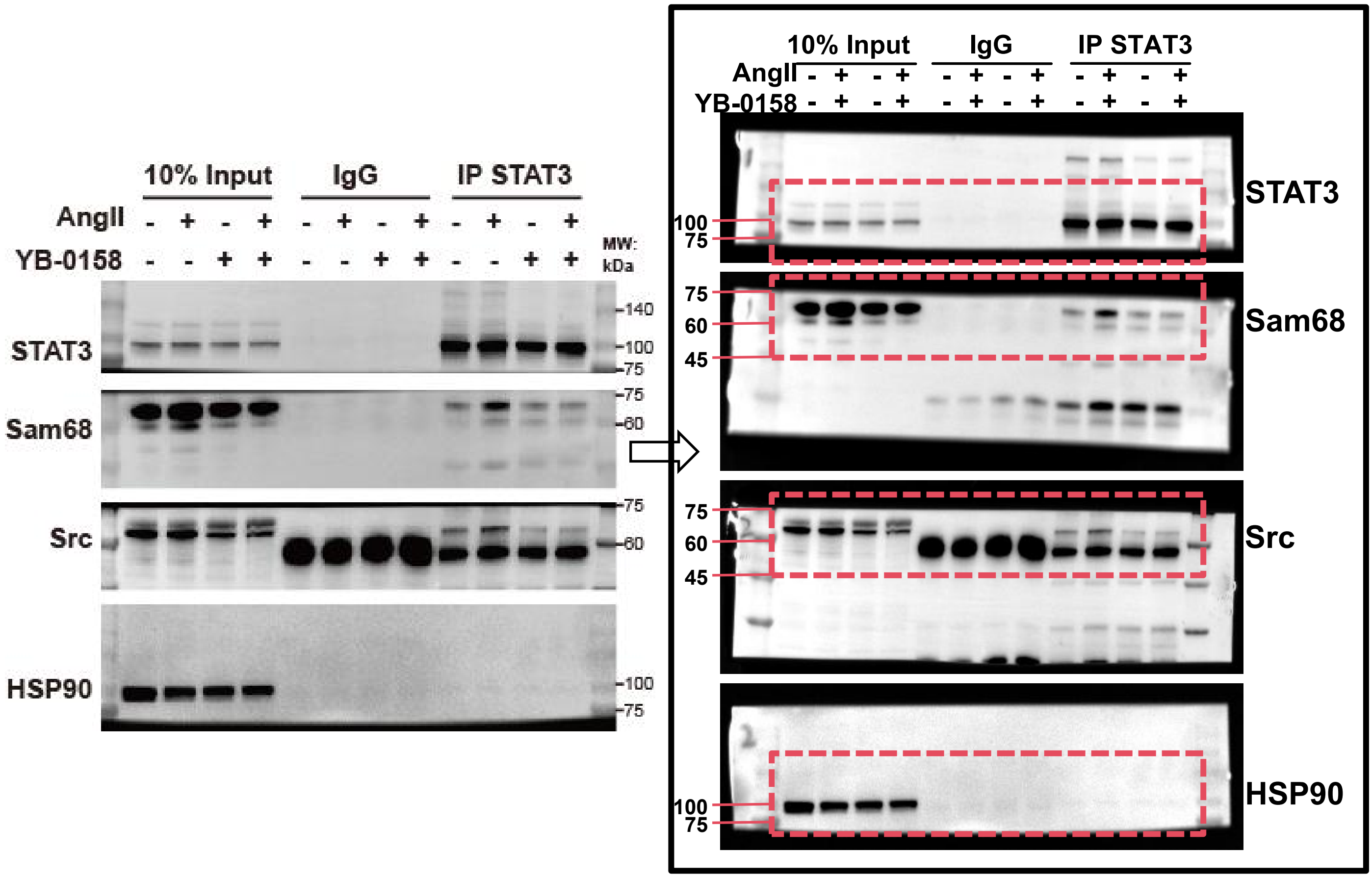

Full unedited gel for Figure 6G

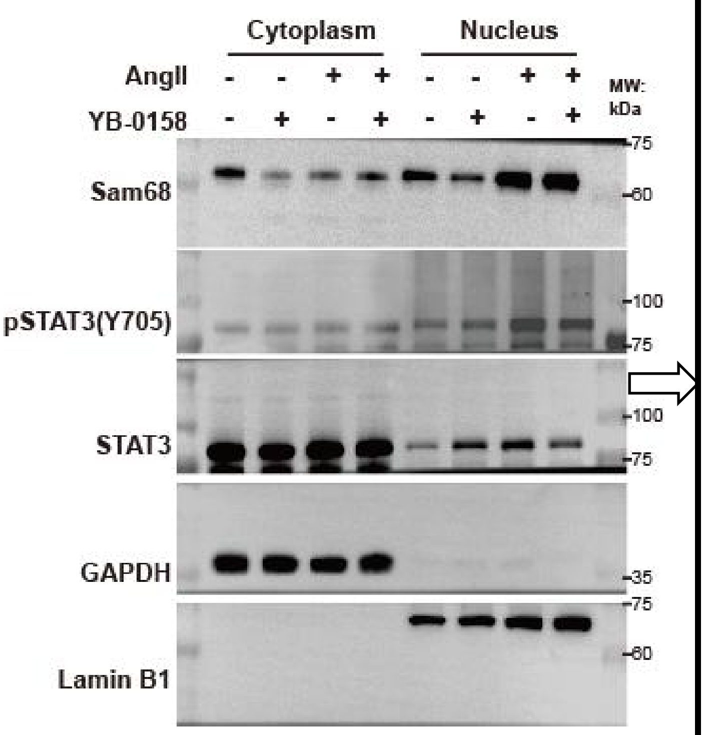

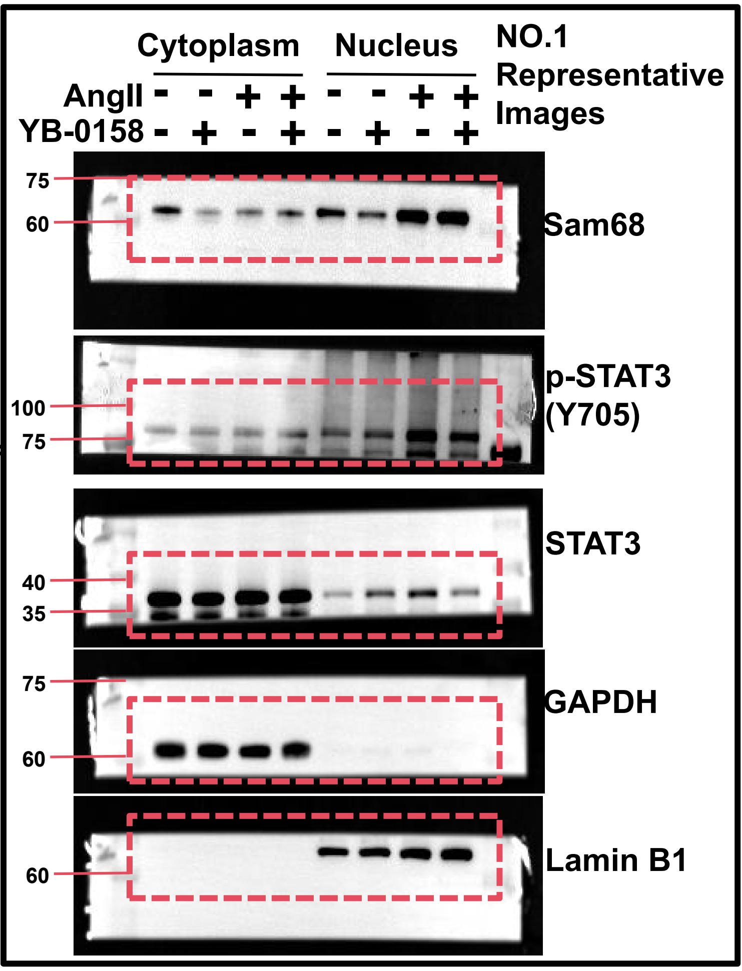

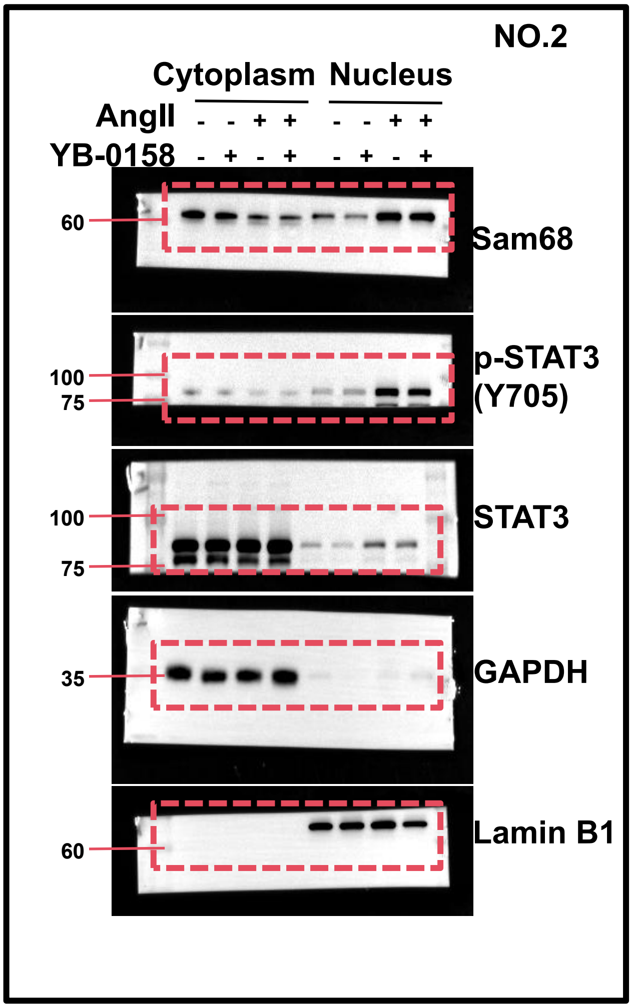

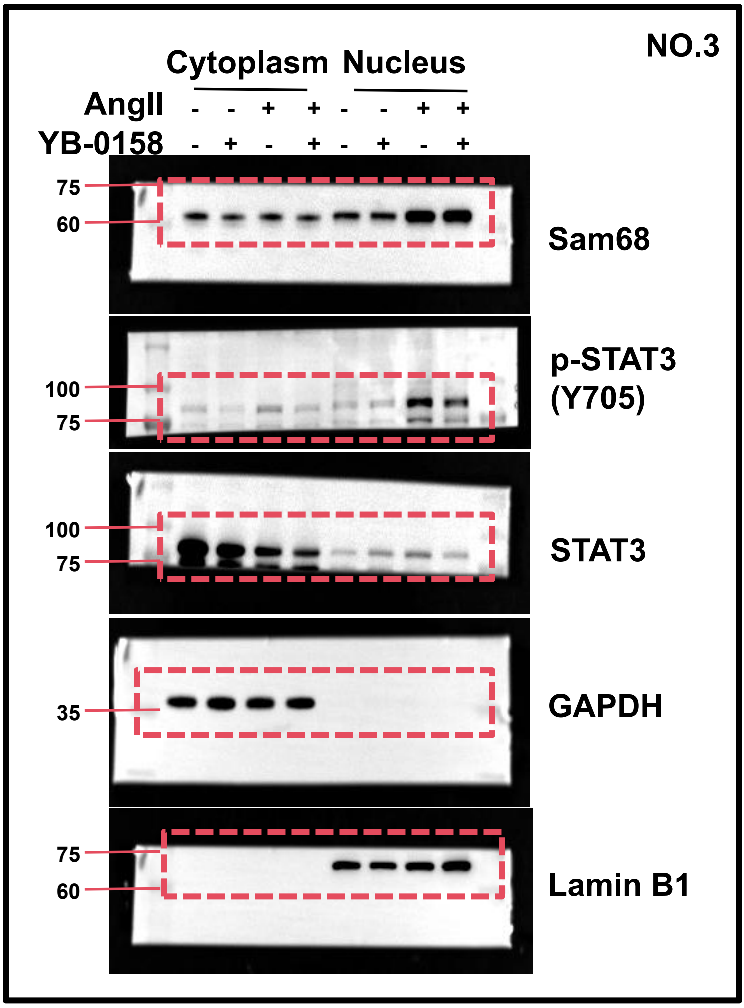

Full unedited gel for Figure 7H

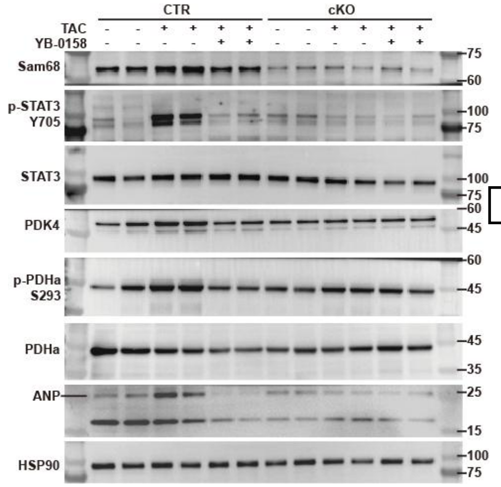

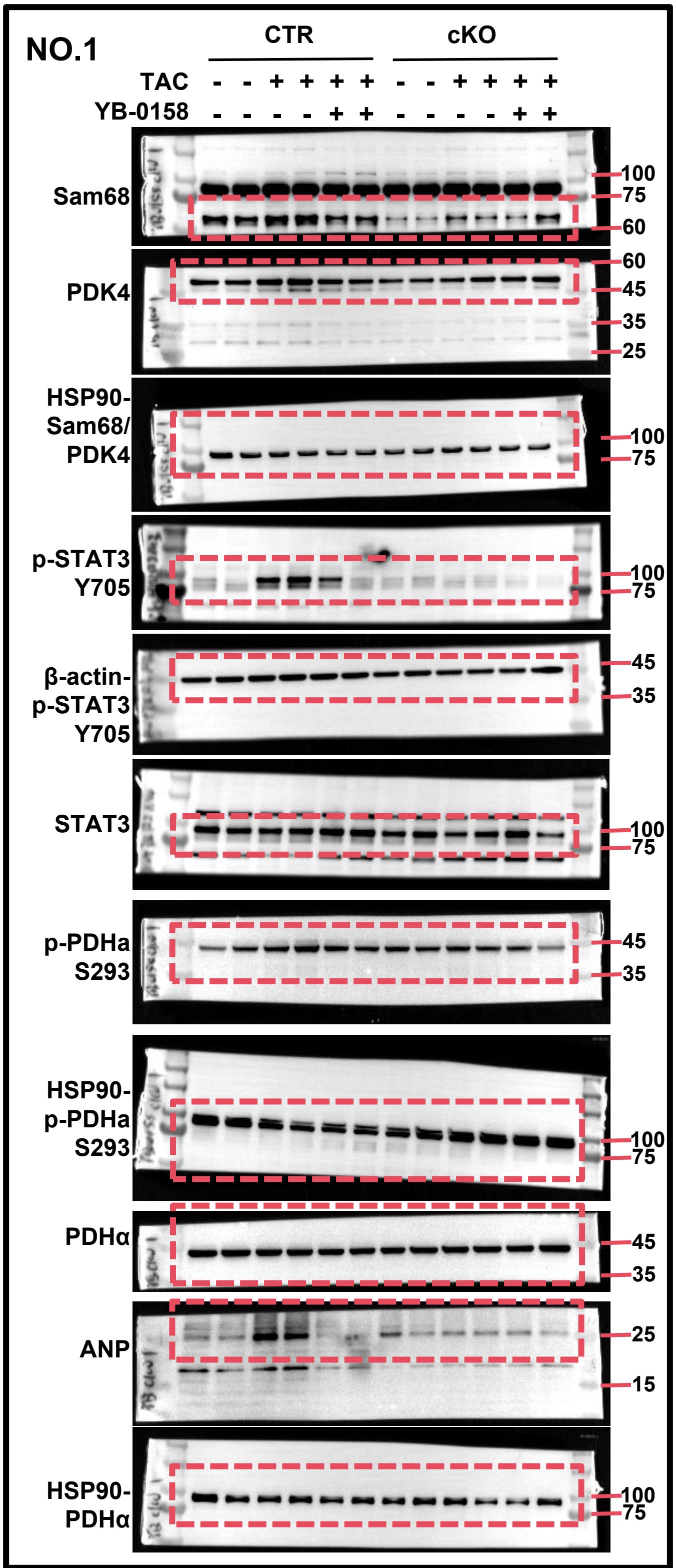

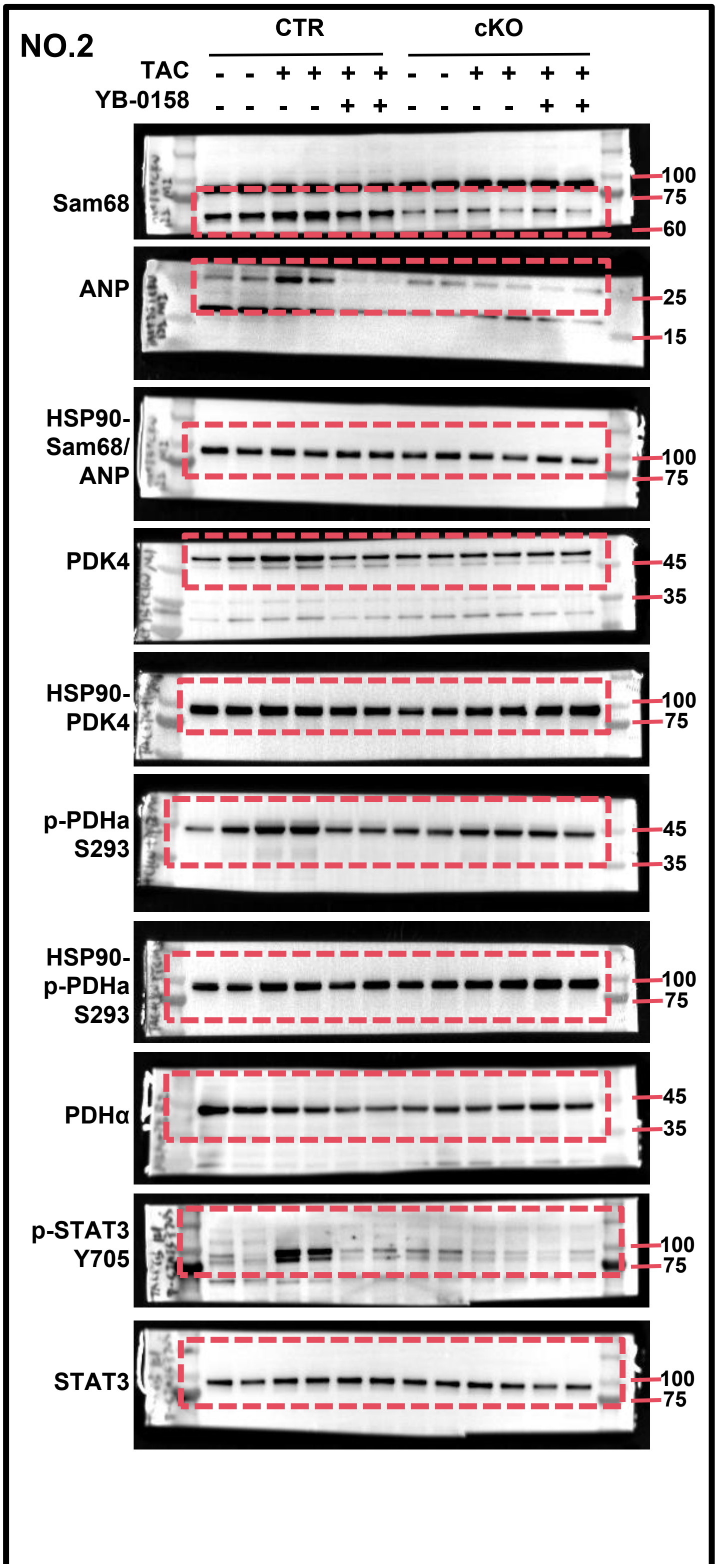

Full unedited gel for Figure 7J

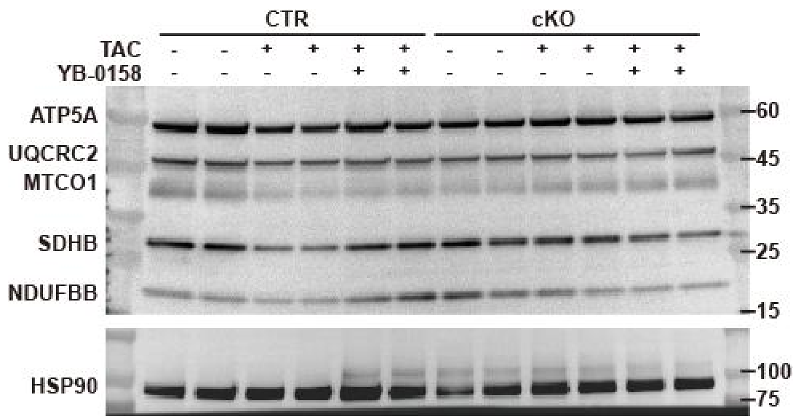

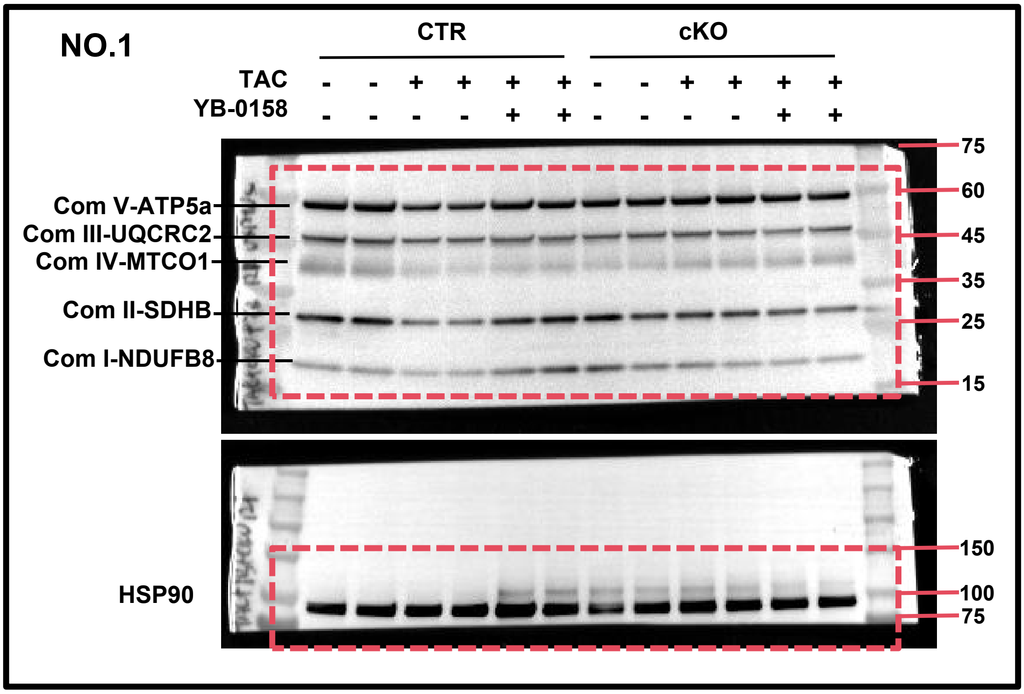

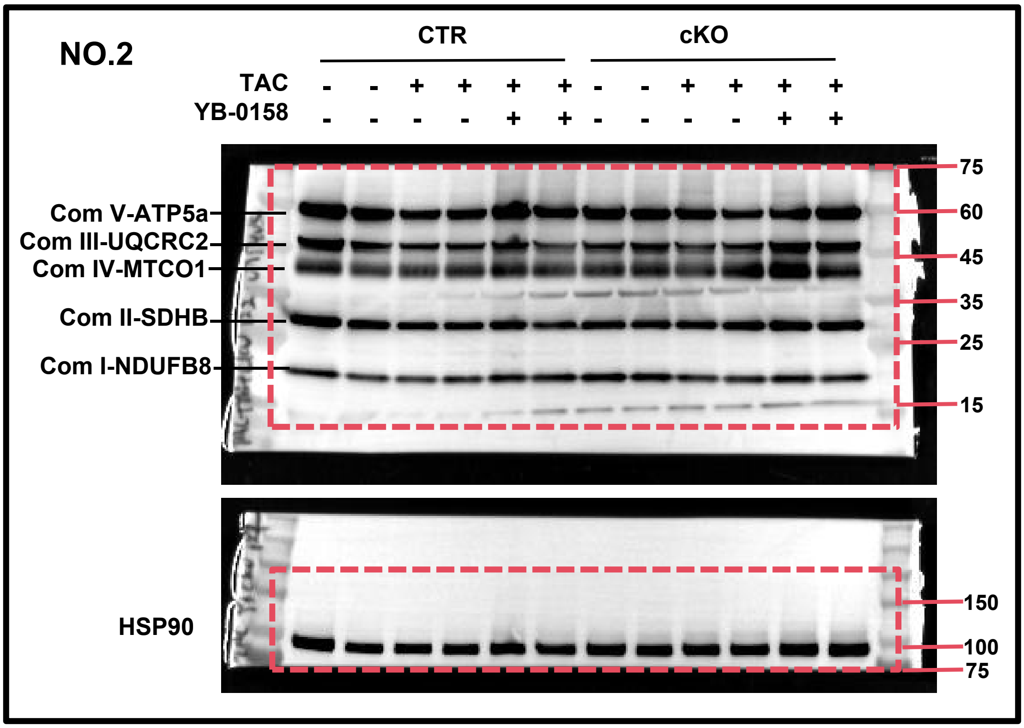

Full unedited gel for Figure 8A

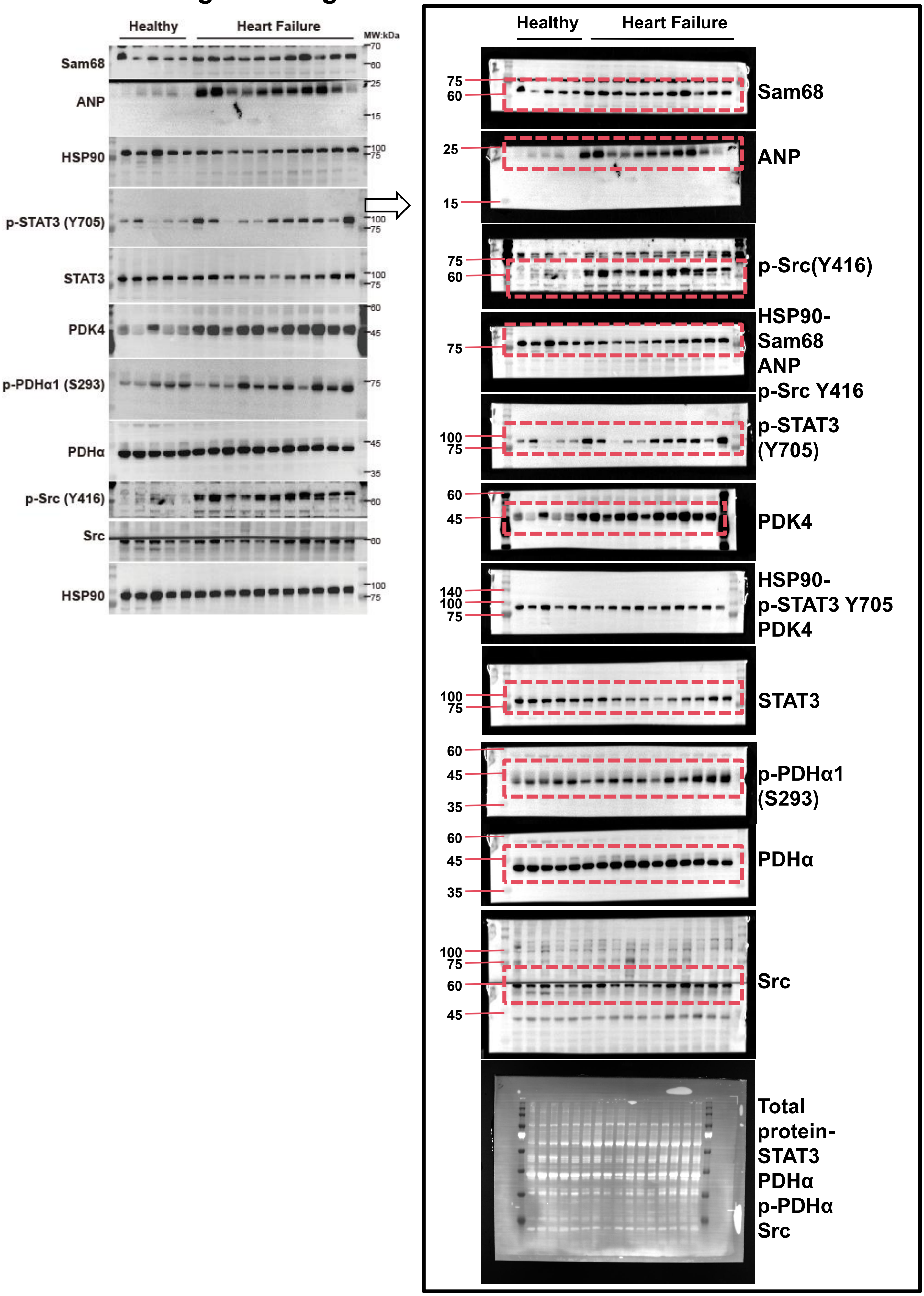

Full unedited gel for Figure S1D

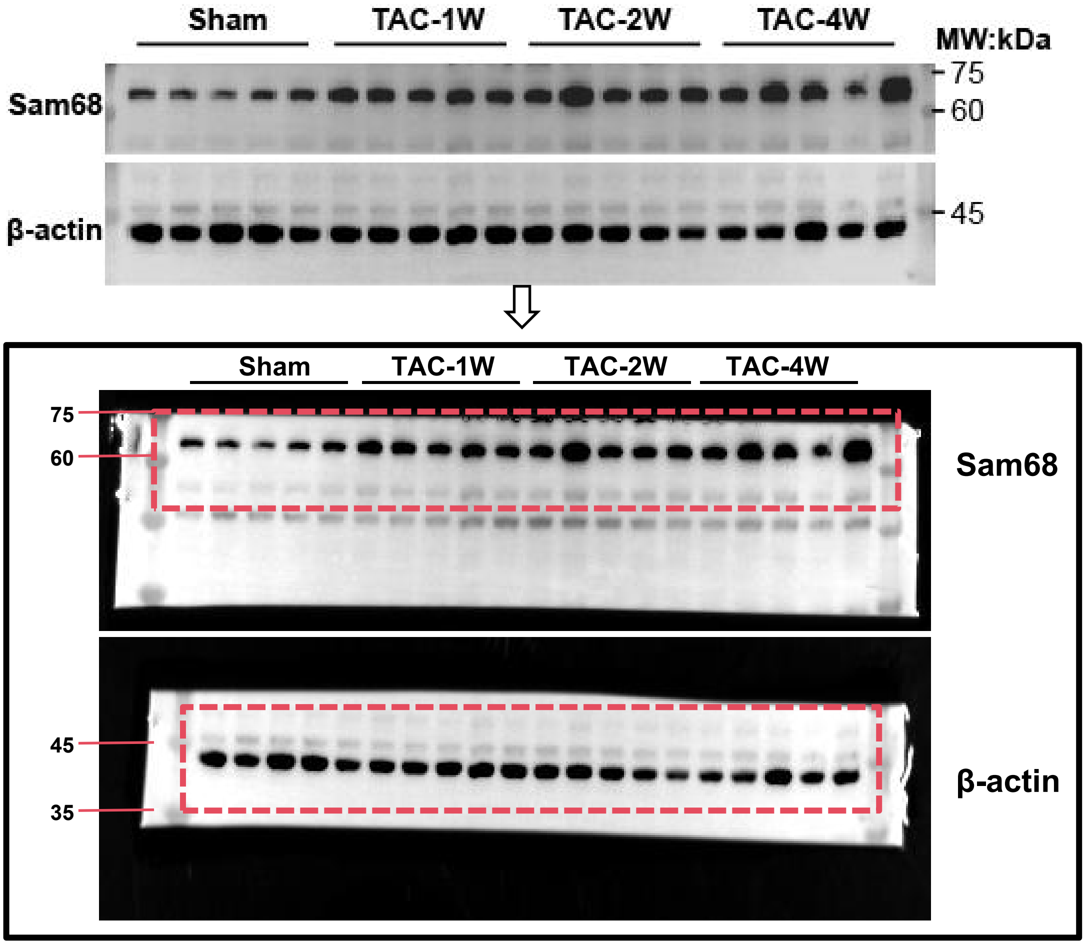

Full unedited gel for Figure S1E

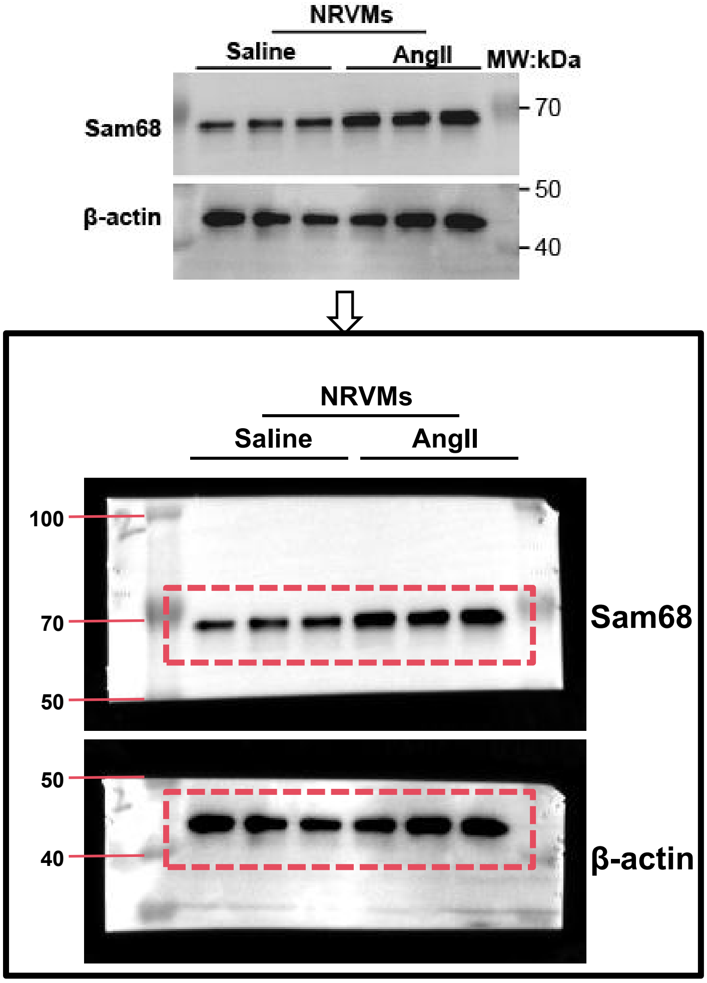

Full unedited gel for Figure S1F

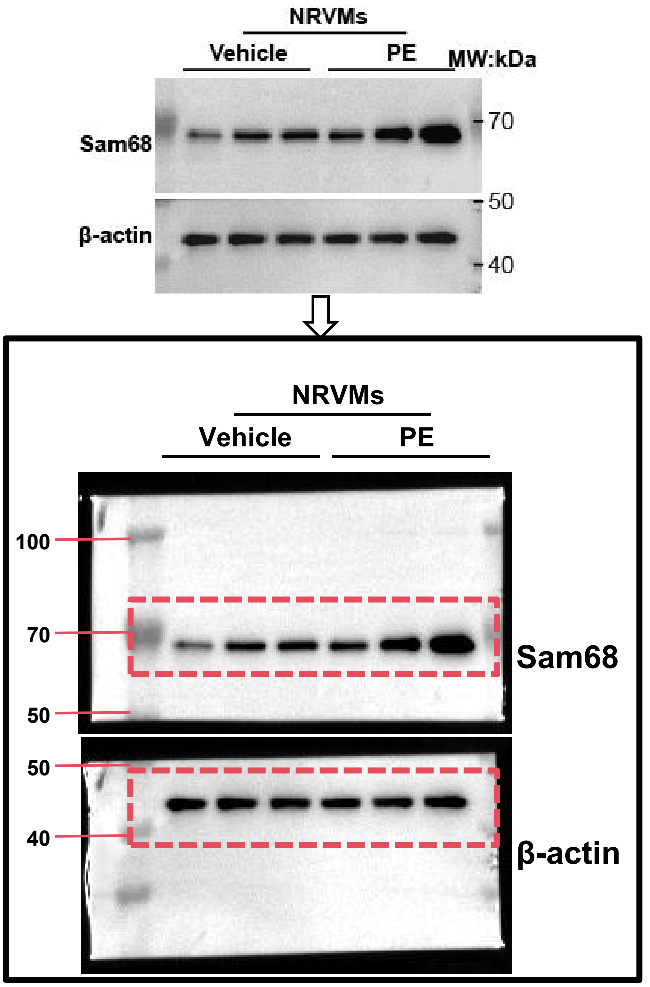

Full unedited gel for Figure S2B

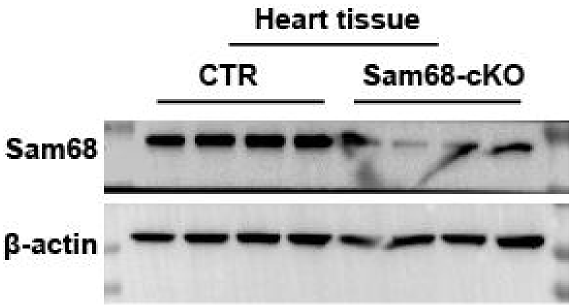

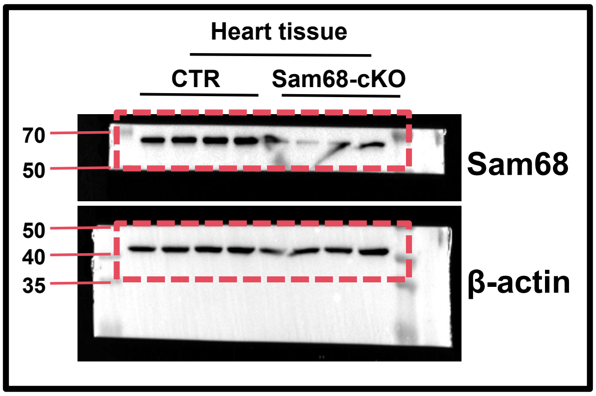

Full unedited gel for Figure S3G

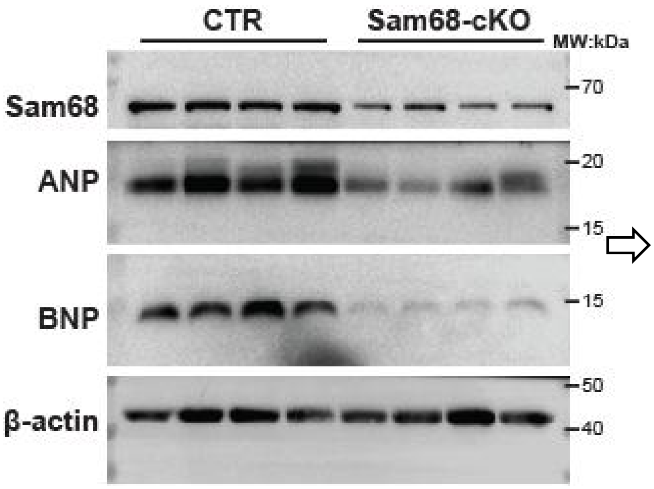

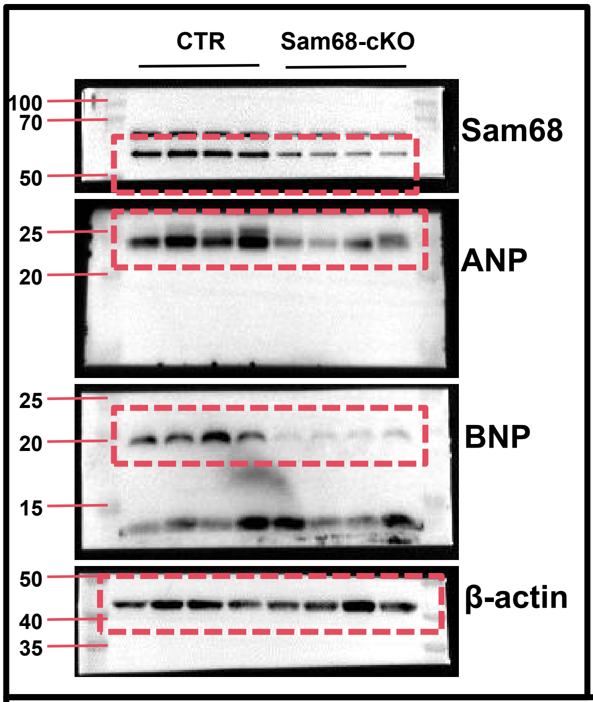

Full unedited gel for Figure S4B

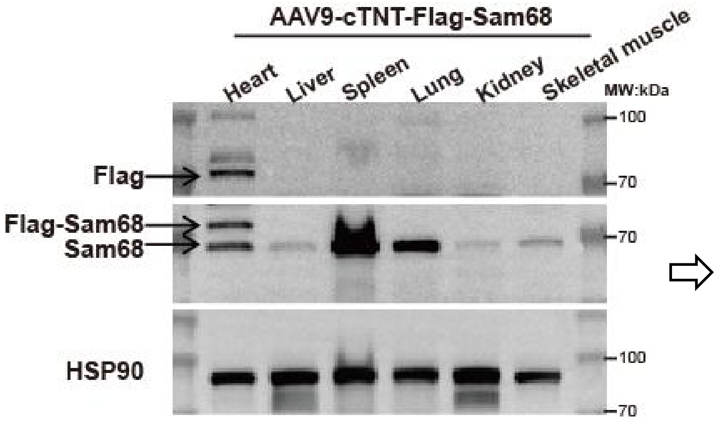

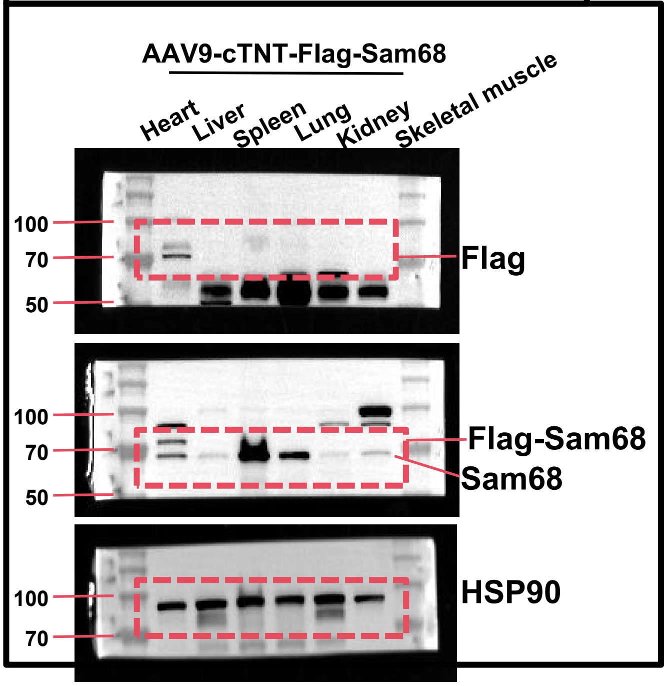

Full unedited gel for Figure S6A

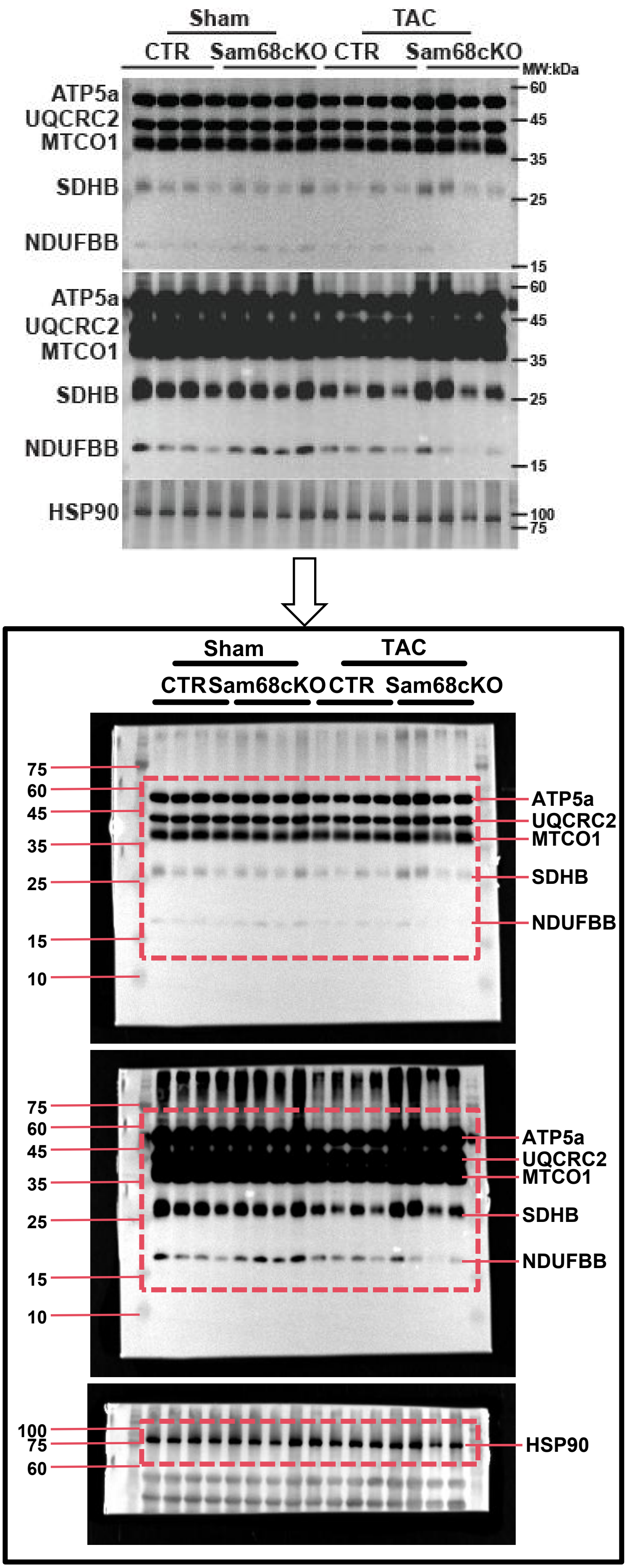

Full unedited gel for Figure S6C

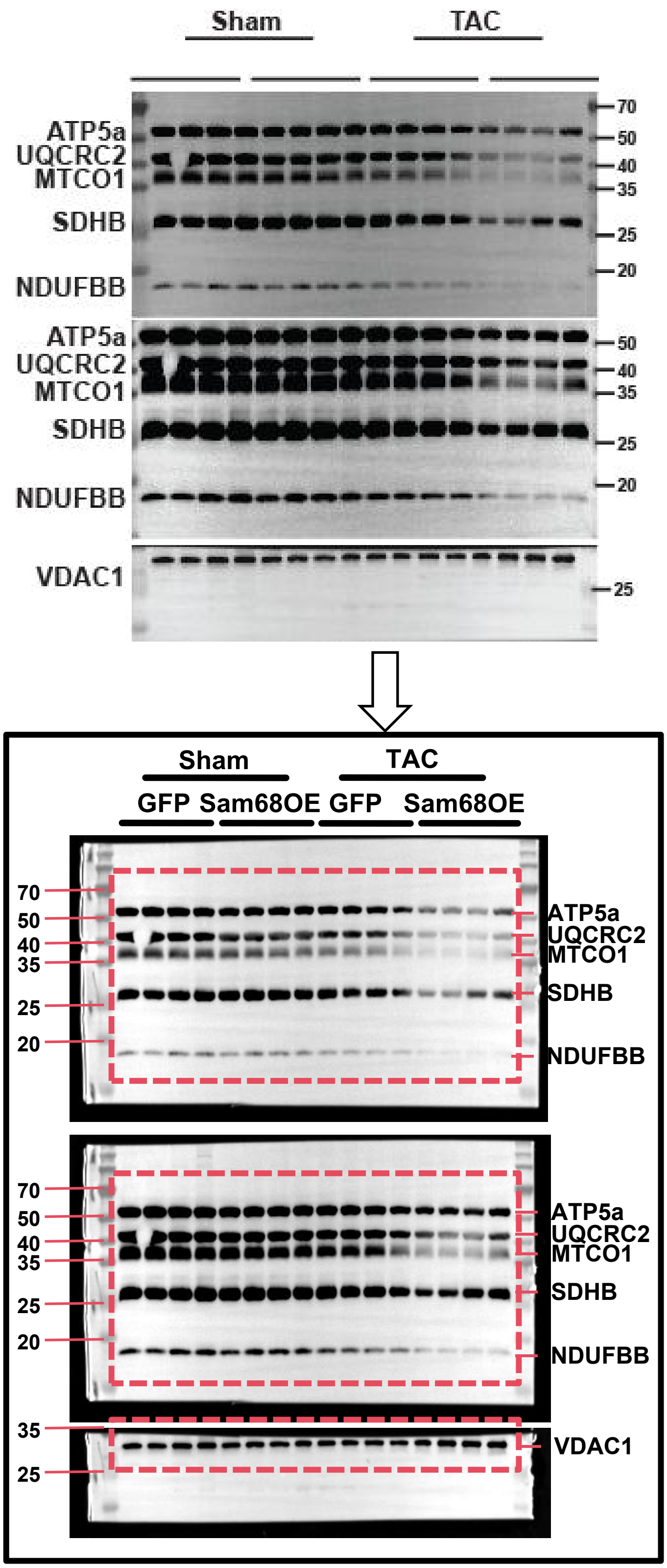

Full unedited gel for Figure S8G

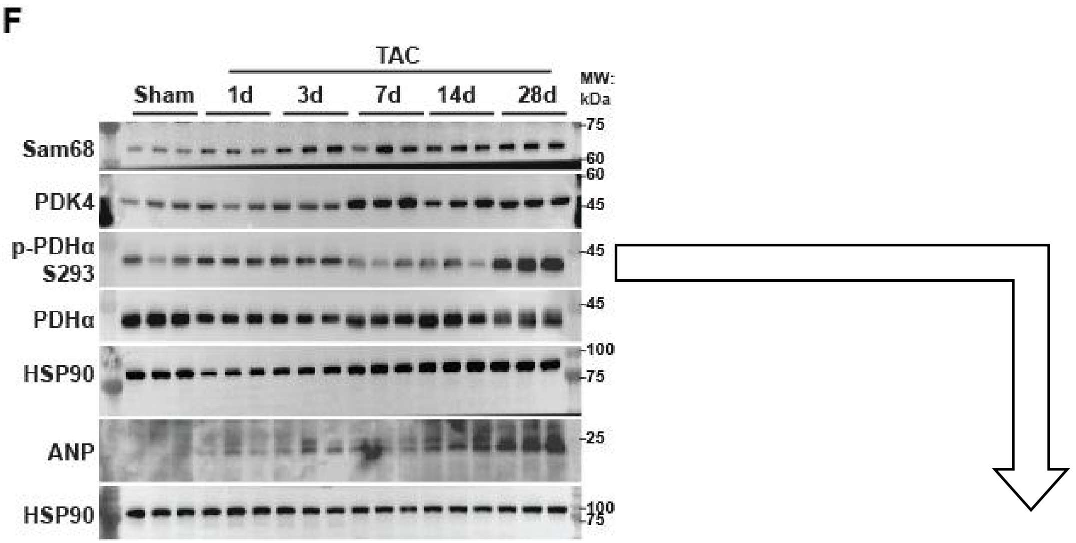

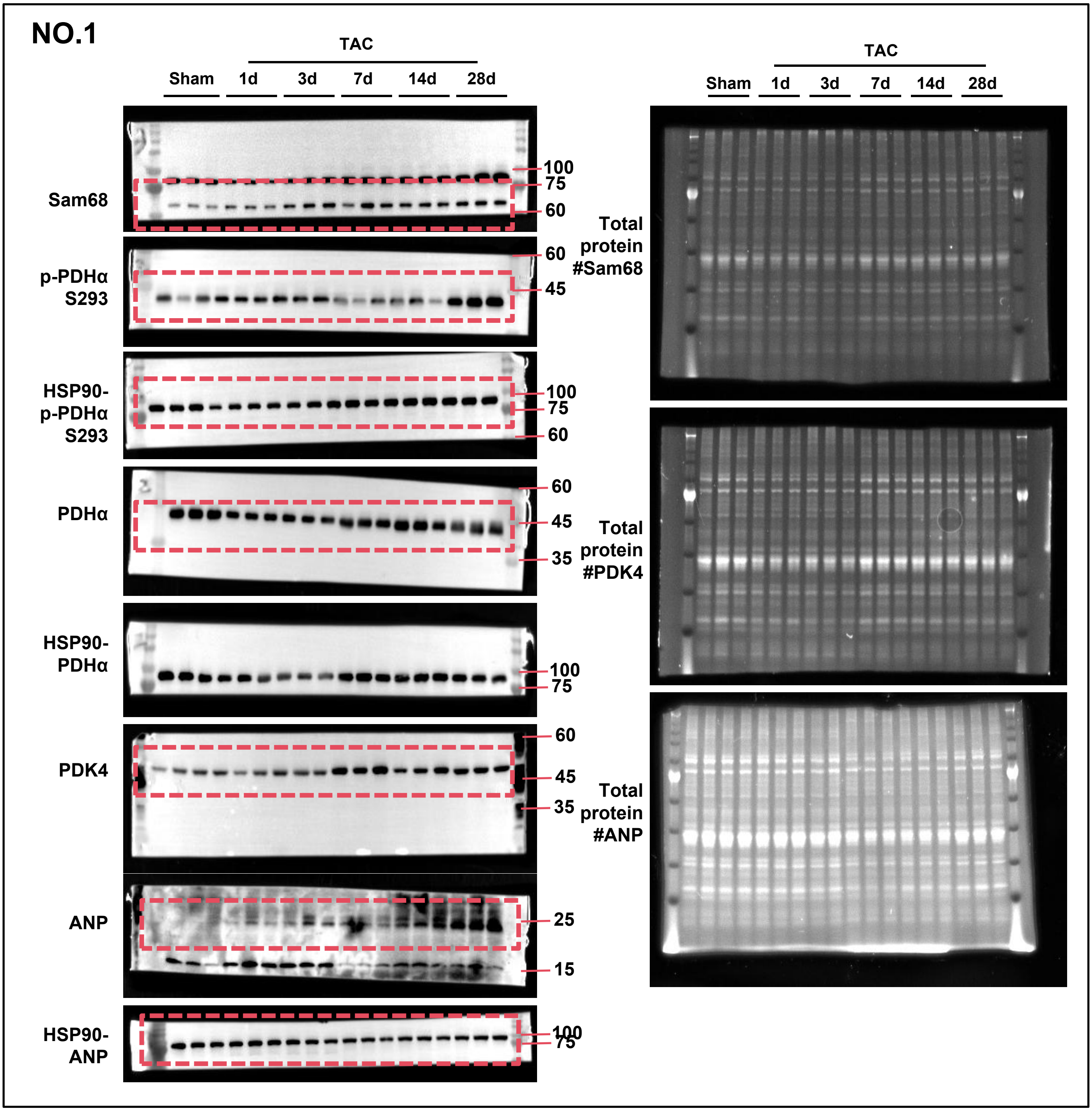

Full unedited gel for Figure S8G

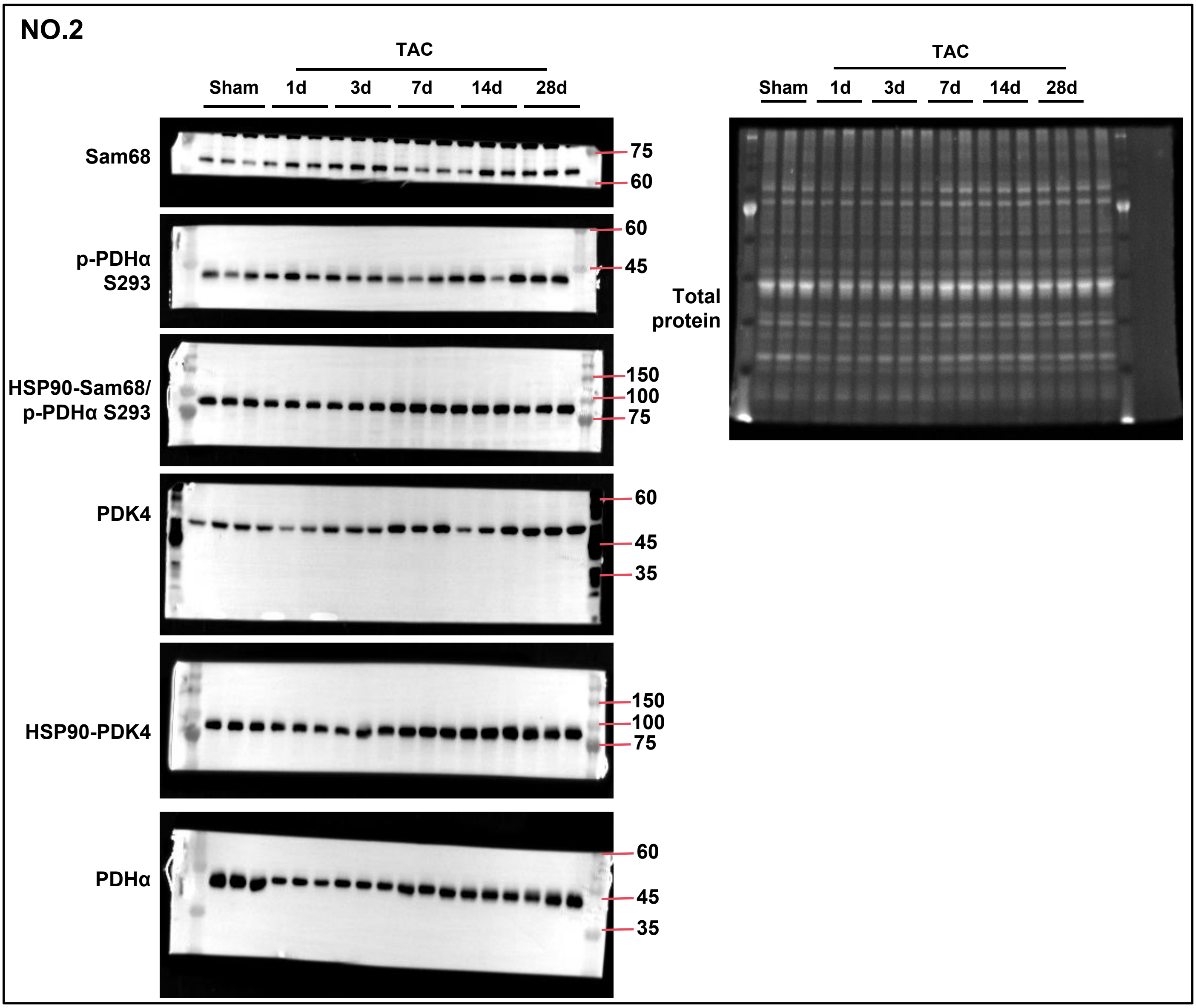

Full unedited gel for Figure S9A

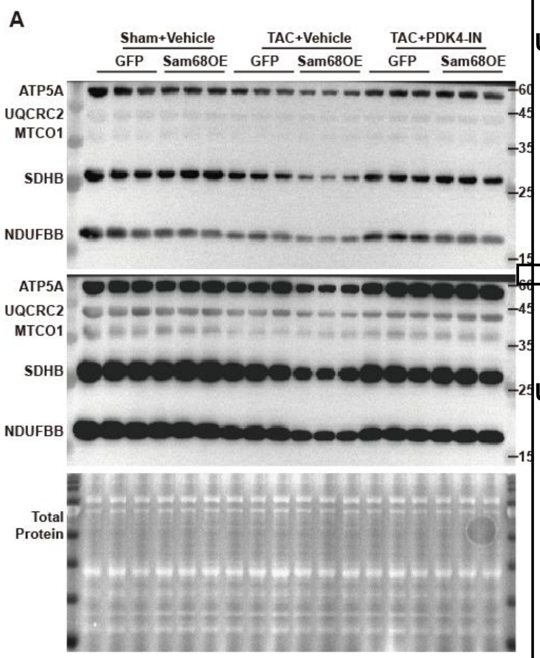

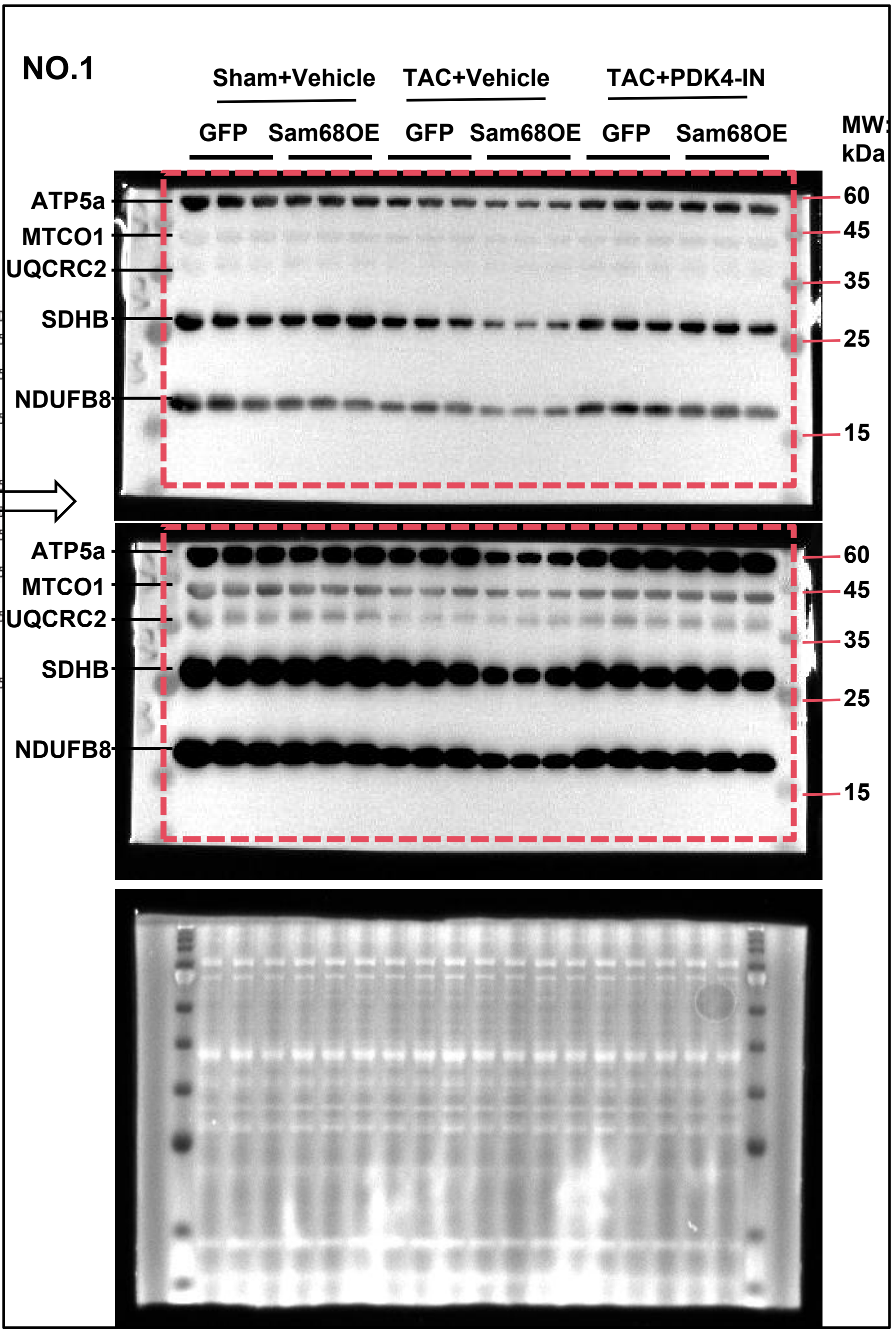

Full unedited gel for Figure S9A

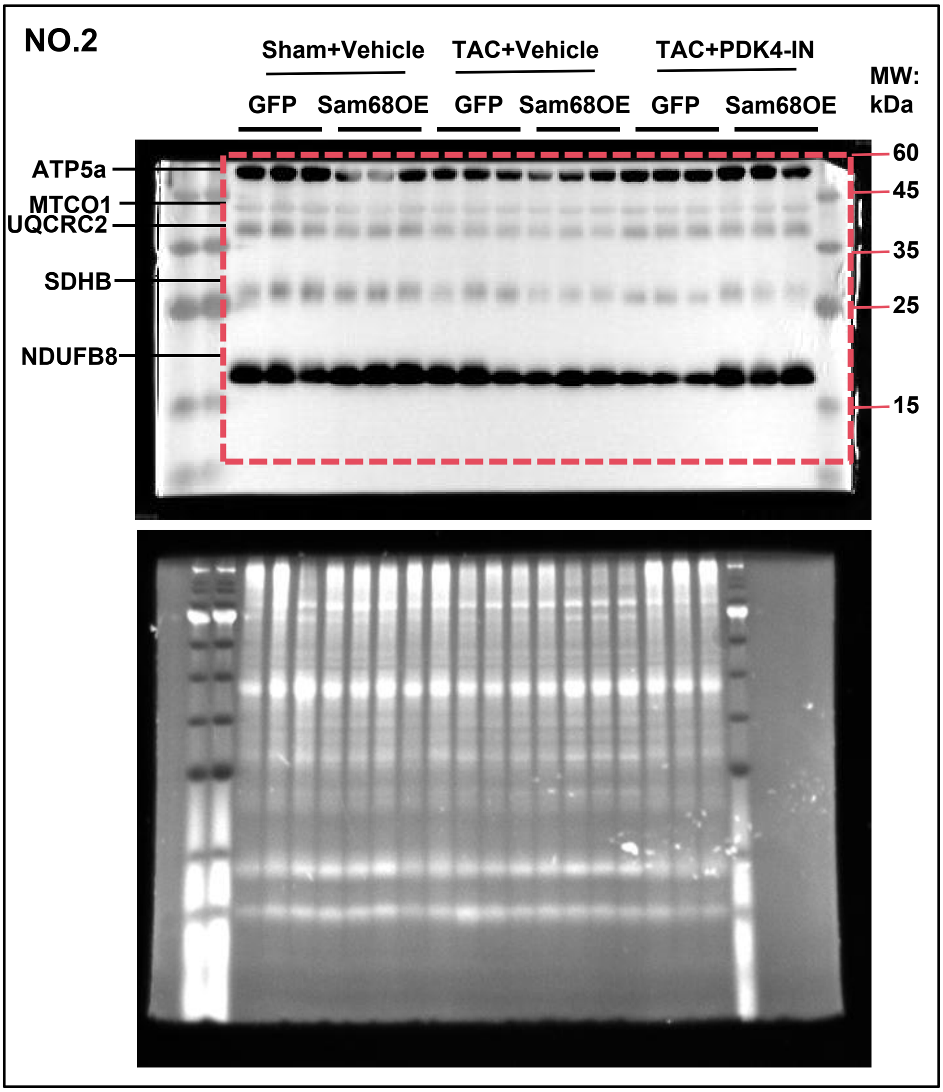

Full unedited gel for Figure S10C

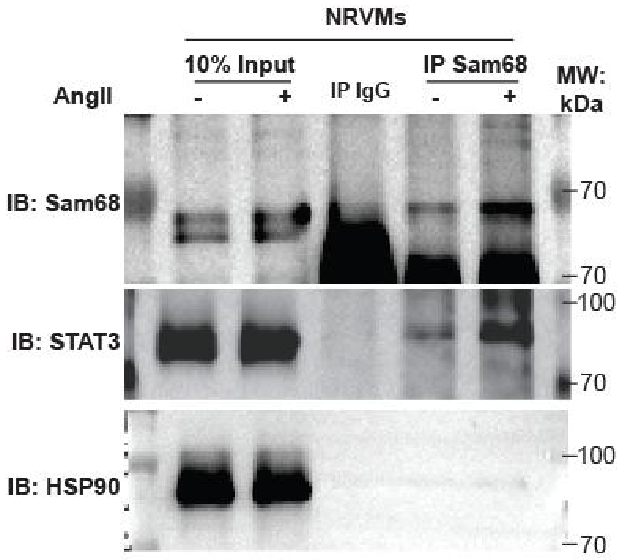

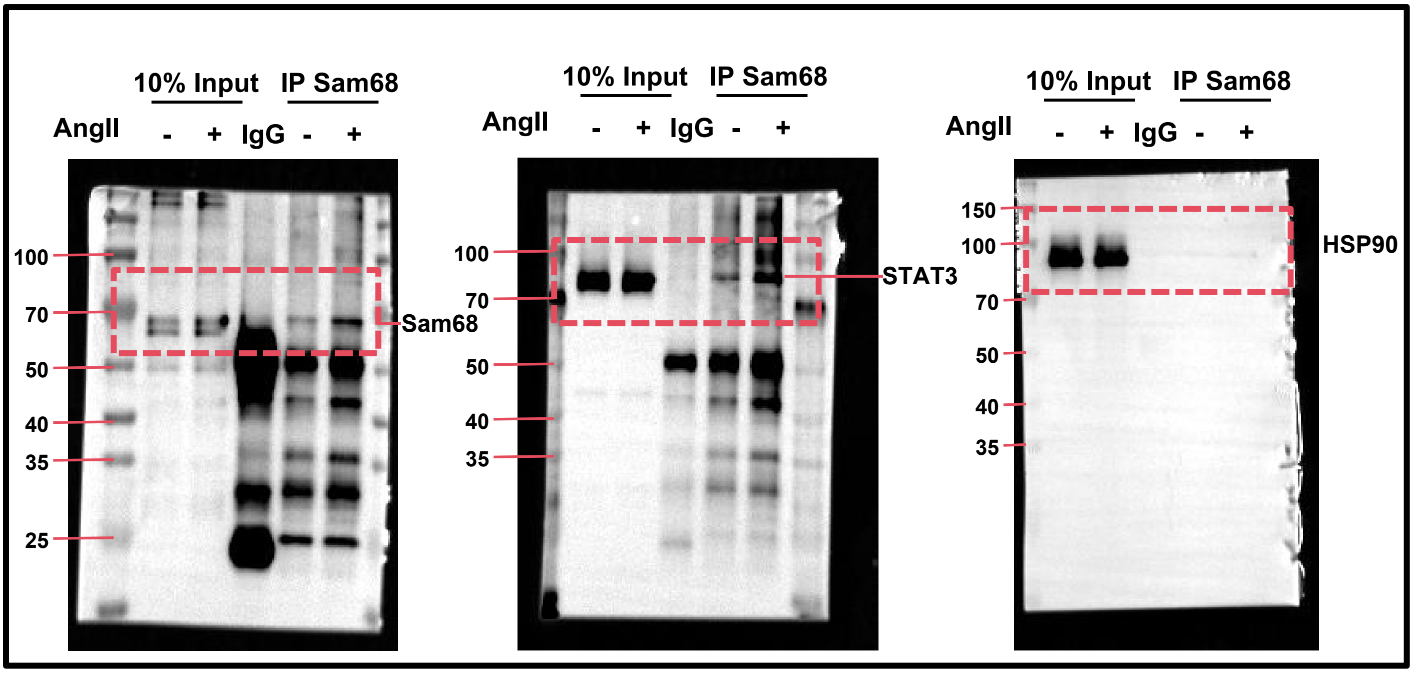

Full unedited gel for Figure S10D

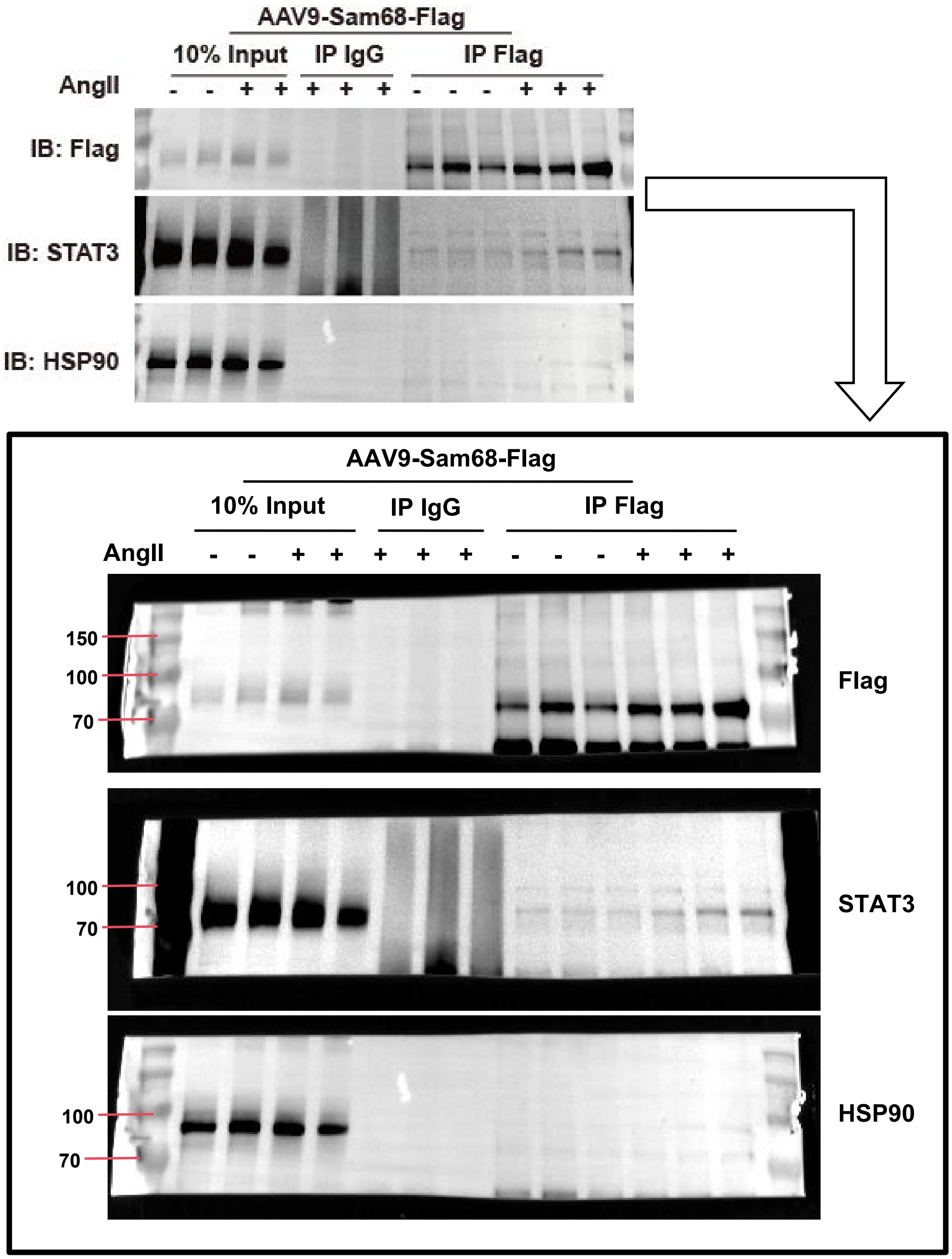

Full unedited gel for Figure S10F

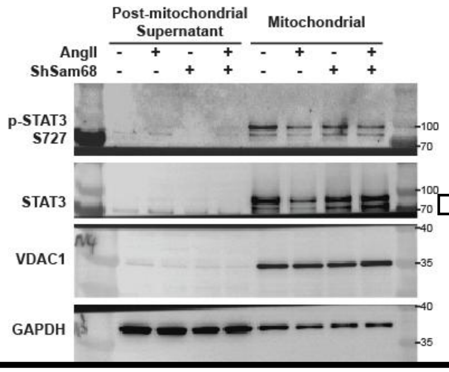

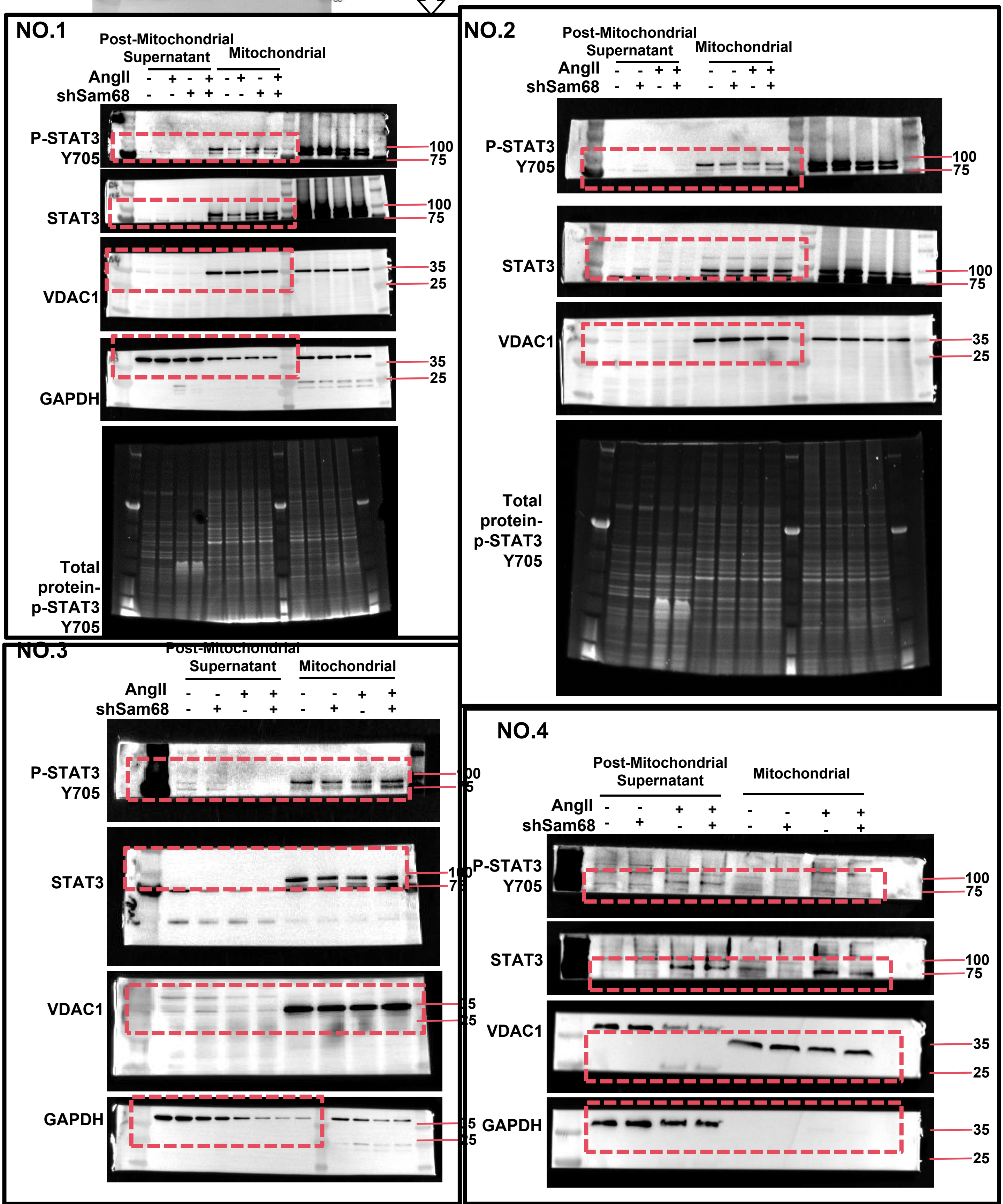

Full unedited gel for Figure S11A

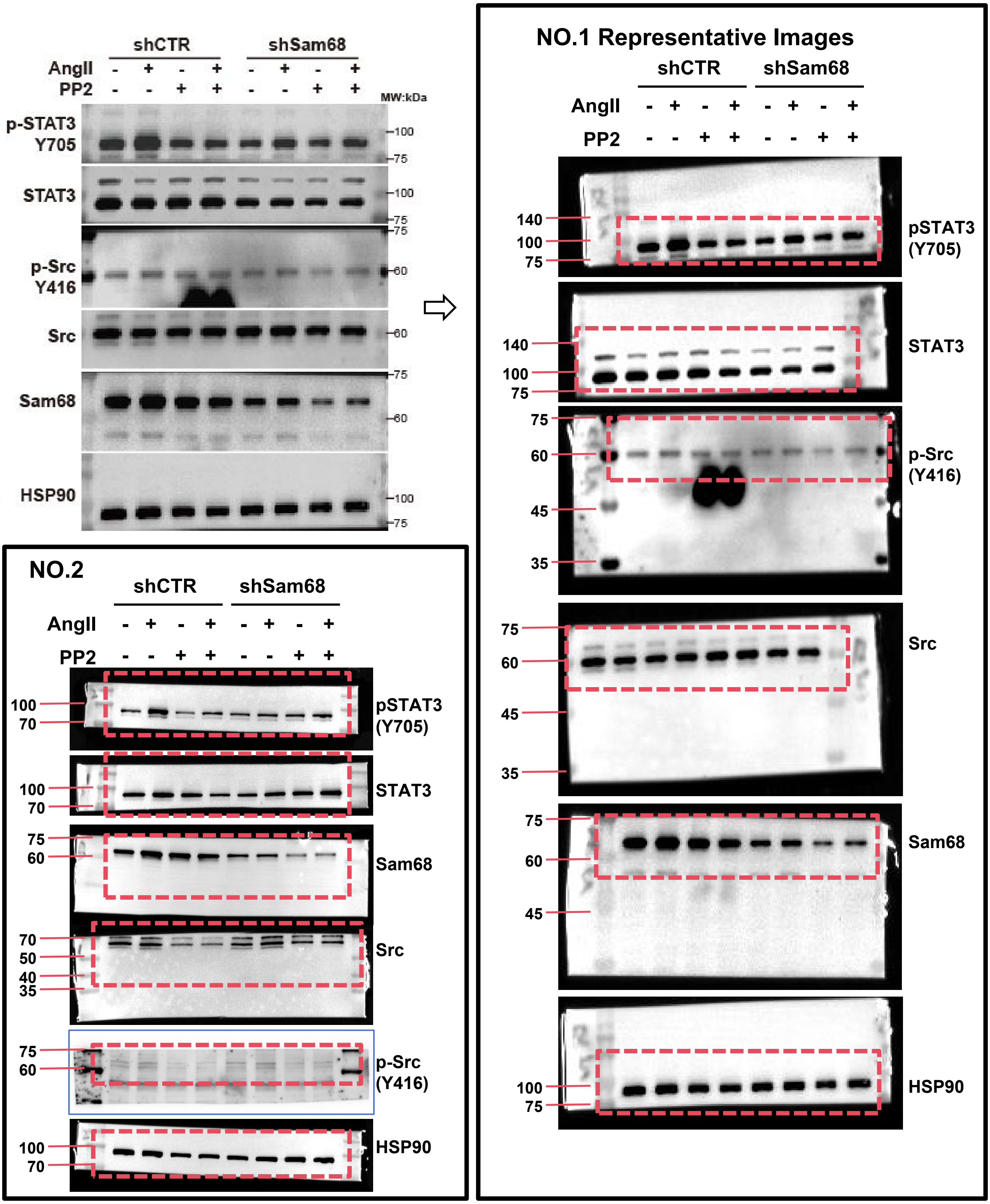

Full unedited gel for Figure S11A

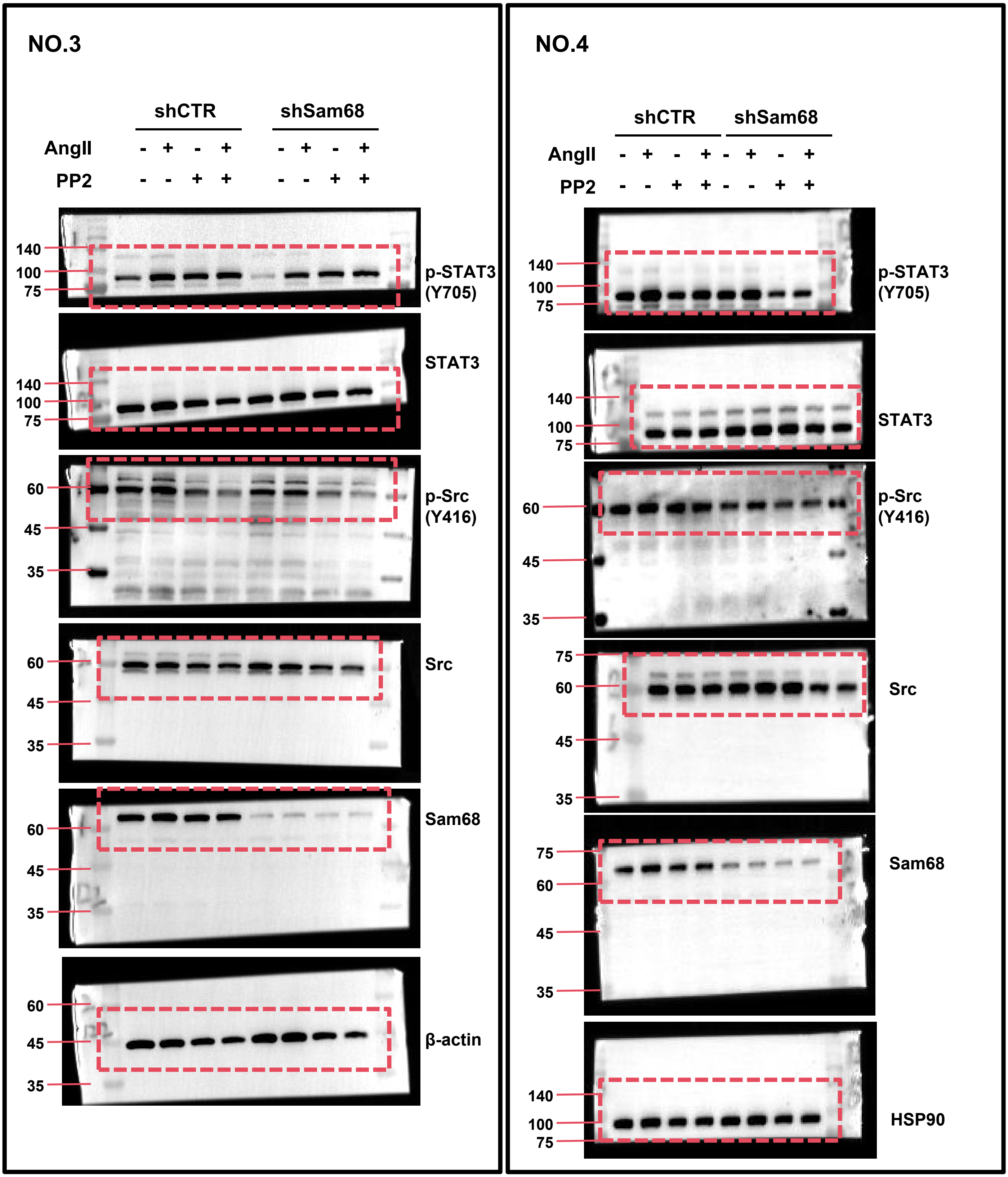

Full unedited gel for Figure S11B

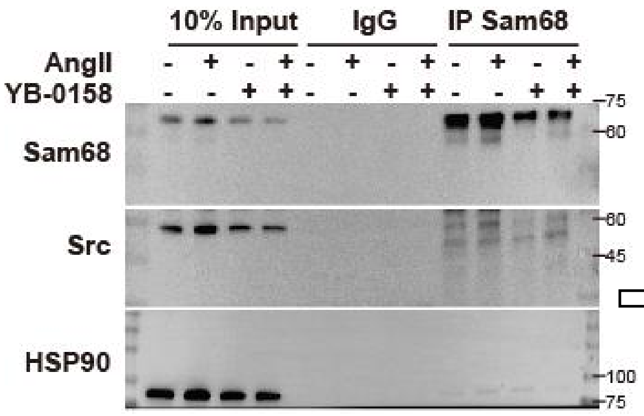

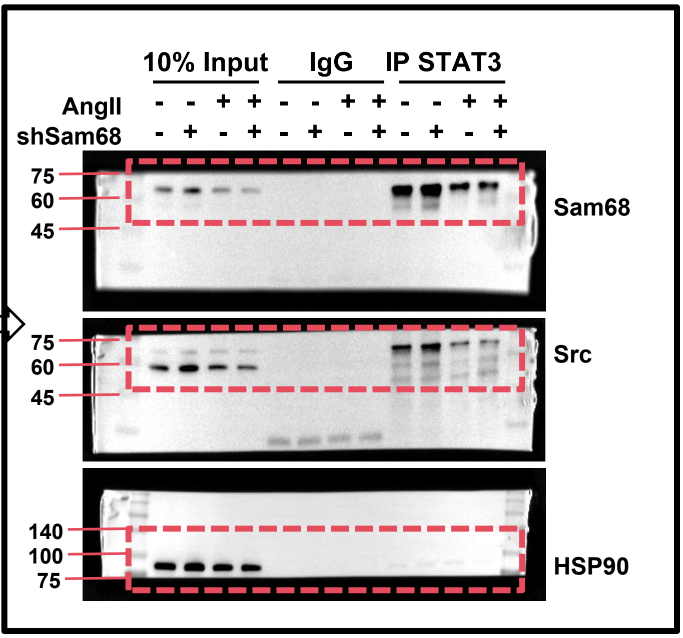

Full unedited gel for Figure S12E

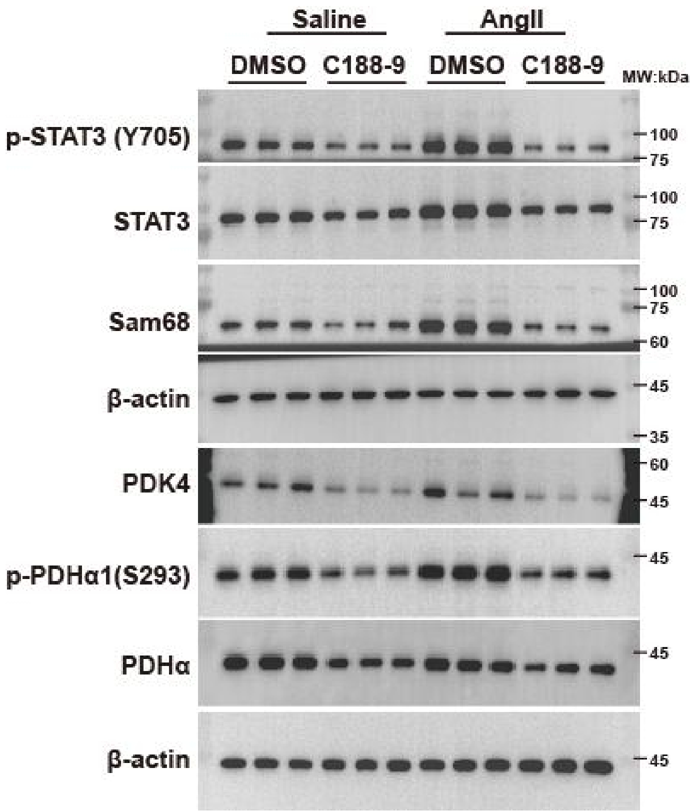

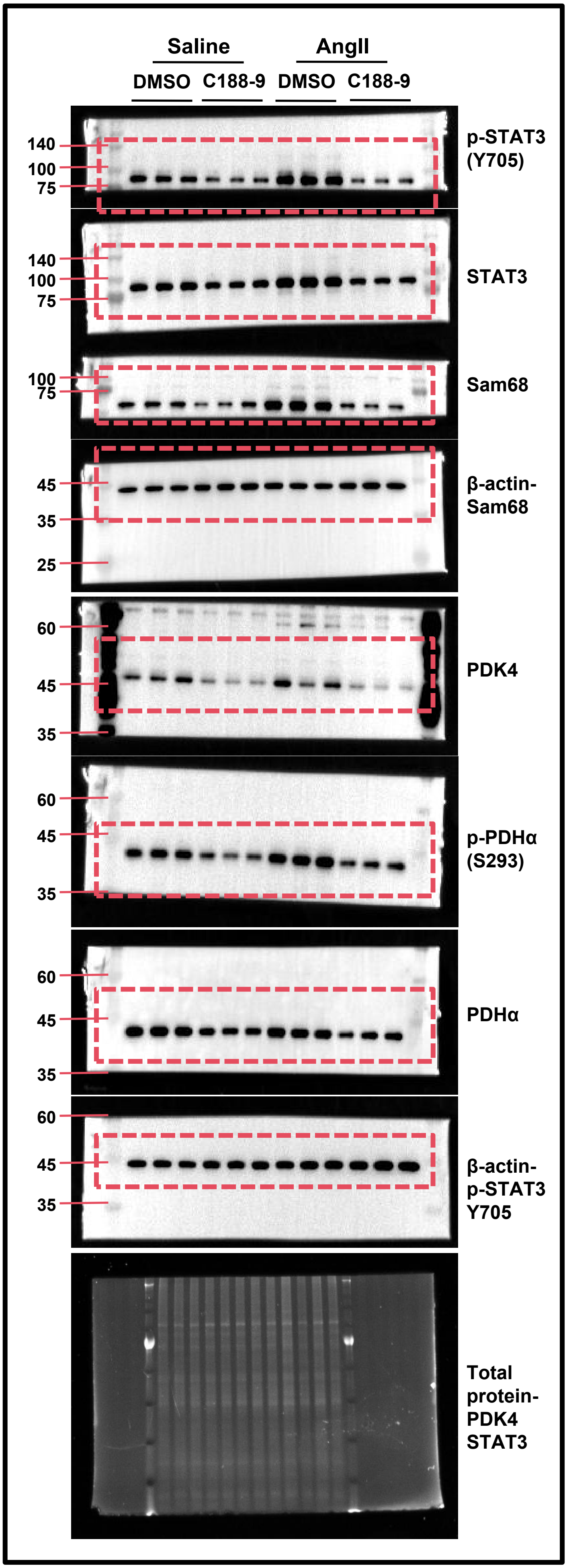

Full unedited gel for Figure S13C

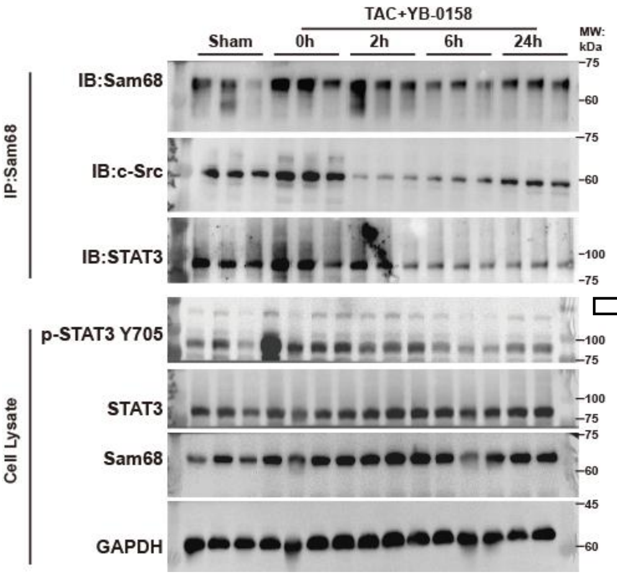

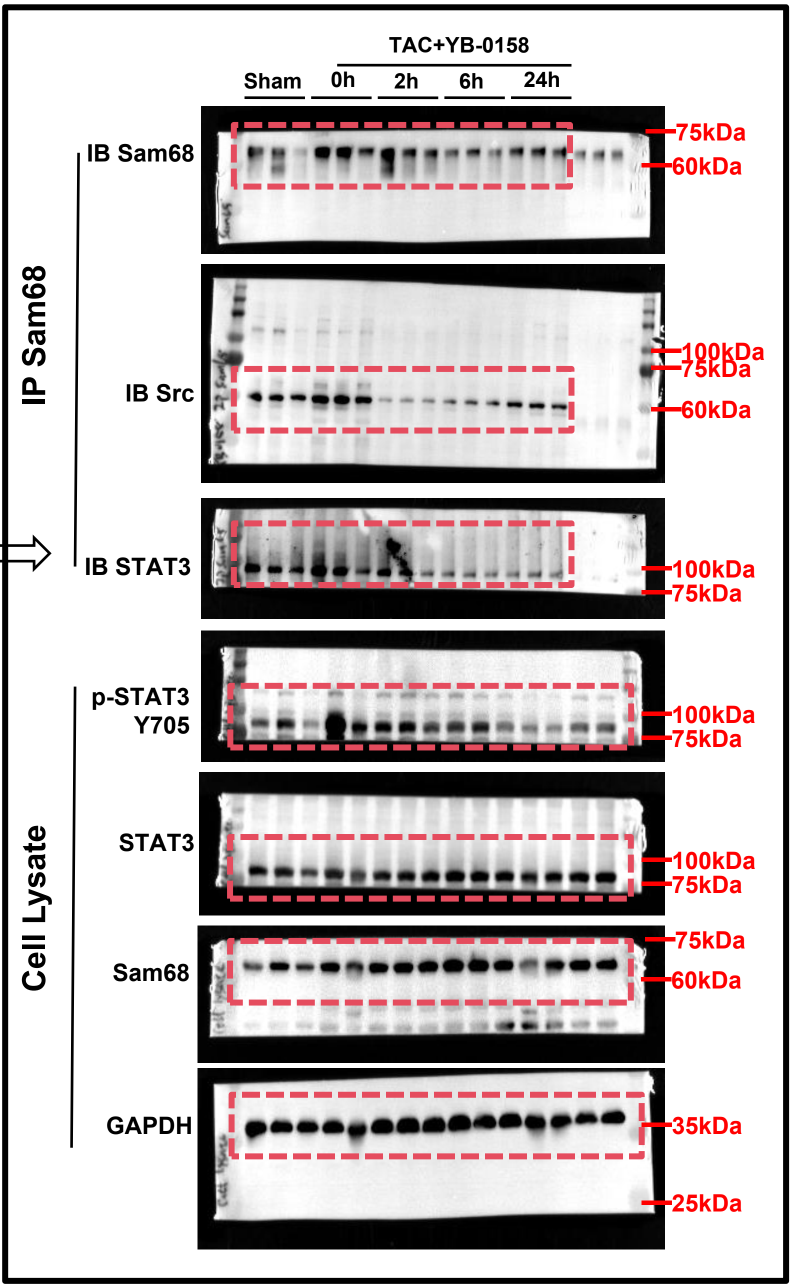

Full unedited gel for Figure S14F

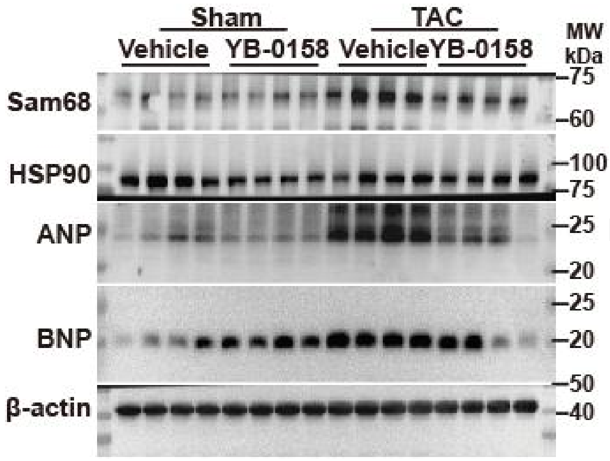

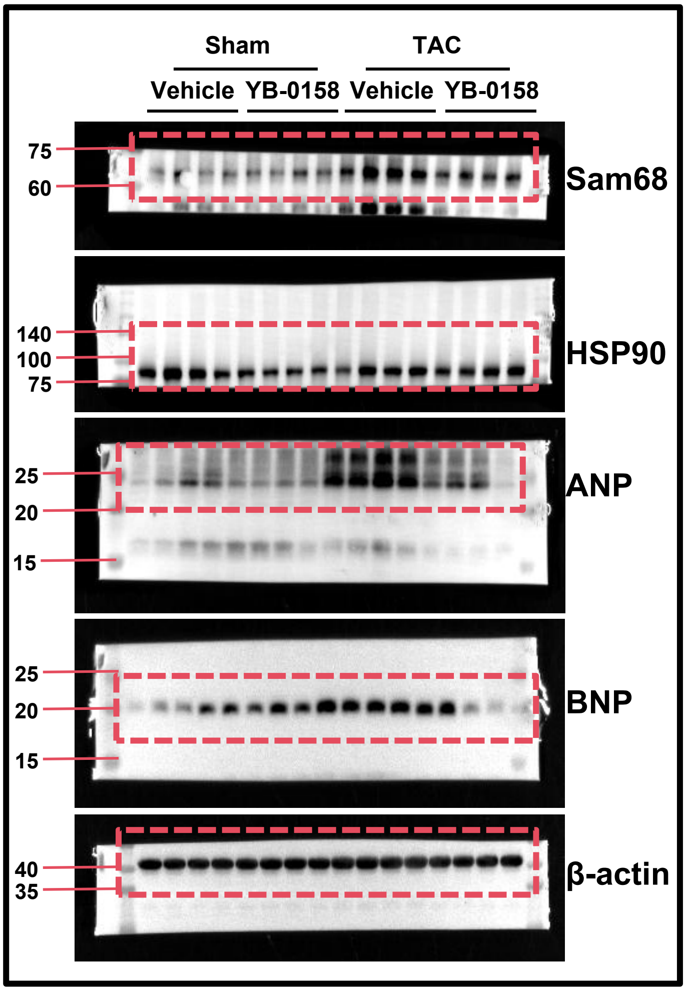

Supplement: Supplementary file 4 [file cir-153-2044-s004.pdf]
